# Programmed cell death-1 receptor-mediated regulation of Tbet^+^NK1.1^−^ innate lymphoid cells within the tumor microenvironment

**DOI:** 10.1073/pnas.2216587120

**Published:** 2023-04-25

**Authors:** Jing Xuan Lim, Chester Y. Lai, Grace E. Mallett, David McDonald, Gillian Hulme, Stephanie Laba, Andrew Shapanis, Megan Payne, Warren Patterson, Michael Alexander, Jonathan Coxhead, Andrew Filby, Ruth Plummer, Penny E. Lovat, Giuseppe Sciume, Eugene Healy, Shoba Amarnath

**Affiliations:** ^a^Newcastle University Biosciences Institute, Medical School, Newcastle University, Newcastle upon Tyne NE2 4HH, United Kingdom; ^b^Newcastle University Centre for Cancer, Medical School, Newcastle University, Newcastle upon Tyne NE2 4HH, United Kingdom; ^c^Dermatopharmacology, Clinical and Experimental Sciences, Faculty of Medicine, University of Southampton, Southampton SO16 6YD, United Kingdom; ^d^Dermatology, University Hospital Southampton NHS Foundation Trust, Southampton SO16 6YD, United Kingdom; ^e^Newcastle University Translational and Clinical Research Institute, Medical School, Newcastle University, Newcastle upon Tyne NE2 4HH, United Kingdom; ^f^Department of Molecular Medicine, Sapienza University of Rome Laboratory affiliation to Institute Pasteur Italia-Fondazione Cenci Bolognetti, Rome 00161, Italy

**Keywords:** ILC, melanoma, PD-1, cSCC

## Abstract

Innate lymphoid cells (ILCs) play a key role in tissue-mediated immunity and can be controlled by coreceptor signaling. Here, we define a subset of ILCs that are Tbet^+^NK1.1^−^ and are present within the tumor microenvironment (TME). We show programmed death-1 receptor (PD-1) expression on ILCs within TME is found in Tbet^+^NK1.1^−^ ILCs. PD-1 significantly controlled the proliferation and function of Tbet^+^NK1.1^−^ ILCs in multiple murine and human tumors. We found tumor-derived lactate enhanced PD-1 expression on Tbet^+^NK1.1^−^ ILCs within the TME, which resulted in dampened the mammalian target of rapamycin (mTOR) signaling along with increased fatty acid uptake. In line with these metabolic changes, PD-1-deficient Tbet^+^NK1.1^−^ ILCs expressed significantly increased IFNγ and granzyme B and K. Furthermore, PD-1-deficient Tbet^+^NK1.1^−^ ILCs contributed toward diminished tumor growth in an experimental murine model of melanoma. These data demonstrate that PD-1 can regulate antitumor responses of Tbet^+^NK1.1^−^ ILCs within the TME.

## Significance

This work identifies an innate lymphoid cell subset (Tbet^+^NK1.1^−^) which possesses antitumor function in both experimental murine models of cancer and in human cancers. This immune arm can be harnessed for enhancing checkpoint inhibitor therapy in patients with solid cancers. The biggest challenge in solid cancers is to identify patients who benefit from checkpoint therapies, and we are still unable to define this population. Tbet^+^NK1.1^−^ innate lymphoid cells (ILCs) within cancers is the **missing link **that can define checkpoint therapy success in cancers such as melanoma and cutaneous squamous cell carcinoma.

Innate lymphoid cells (ILCs) are counterparts of CD4^+^ helper T (Th) cells with diverse phenotypes and functions that mirror their respective Th effector subsets (1). ILCs are classified into three groups, with natural killer (NK) cells and lymphoid tissue-inducing cells being the first to be identified. In recent years, this family of ILCs has expanded and recent nomenclature has classified them into three groups that mirror respective T effector cell lineages (1): Group 1 ILCs include NK cells and non-NK cells, constitutively expressing the transcription factor T-bet and respond to type 1 cytokines, namely IL-12, IL-15, and IL-18 by producing IFNγ and TNFα (2–4). The expression of Eomes within group 1 ILCs further enables the differentiation between NK cells and helper-like ILC-1s (5, 6). Mostly, ILC-1s are defined as Lin^-^NKp46^+^NK1.1^+^CD49a^+^CD49b^−^ cells while NK cells are identified as subsets that are CD49b^+^. However, significant redundancies occur within ILC-1s which renders their classification difficult within biological systems in mice and humans. Unlike Group 1 ILC, Group 2 ILCs are marked by the transcription factor GATA3 (7–10). Group 3 ILCs express the transcription factor RORγt and produce cytokines IL-17 and/or IL-22 and can be further divided based on NKp46 expression (11–18).

The role of NK cells in eradicating tumors has been well established, but the function of helper like ILC-1s within the tumor microenvironment (TME) is yet to be fully understood, with implications in pro-tumorigenic effects (19). The function of group 2 ILCs in pro- and anti-tumorigenic responses has also been explored by a number of reports (20–25). Similarly, IL-22-expressing ILC-3s have been implicated in protumor responses in bacteria-induced colon cancer (26, 27). Conversely, antitumor effects of ILC-3s have been demonstrated in a model of malignant melanoma (28). This antitumor function in melanoma is driven by IL-12, which in turn induces T-bet expression in ILC-3s, resulting in the development of T-bet^+^ “ex-ILC-3s” that resemble ILC-1s in phenotype. Despite inconsistencies, emerging literature suggests that the function of ILCs within the TME is dependent on the tissue microenvironment and cytokine availability (29).

While cytokine-mediated regulation of ILCs has been widely investigated, the role of coreceptors in regulating ILC function has been limited. The expressions of several coreceptors on ILCs have been reported but in the absence of an antigen receptor; several questions remain with respect to the functional consequence of coreceptor expression within ILC biology. Despite this lack of knowledge, the expression of these coreceptors provides an avenue for boosting ILC responses in cancer (30). The myriad of coreceptors expressed on ILC include inducible T cell costimulatory (ICOS) and the ligand (ICOS-L) (31), programmed cell death-1 receptor (PD-1) (32) and its ligand PD-L1 (33), glucocorticoid-induced tumor-necrosis-factor-receptor-related protein (GITR) and its ligand GITR-L, Killer cell lectic-like receptor G1 (KLRG1), T-cell immunoglobulin domain and mucin domain 3, lymphocyte-activation gene 3, B and T lymphocyte attenuator, cytotoxic T-lymphocyte antigen-4, and OX40 ligand. The expression pattern of these coreceptors on ILCs within the TME has been comprehensively reviewed in refs. 34 and 35.

Recent literature has identified a role for PD-1 in ILC-2 regulation, in the presence of exogenous IL-33, within the TME (25, 36) but whether PD-1 has an intrinsic function in the absence of exogenous IL-33 on ILC subsets within the TME is not clear. In murine tumors, PD-1 expression is marginally up-regulated (5%) in ILC-1s (19) and a similar expression pattern is noted in infiltrating breast and gastrointestinal (GI) tumors (30). In human hepatocellular carcinomas, (37) PD-1 expression is increased in all ILC subsets, but a functional role was not identified. Hence, while the expression of PD-1 on tumor ILC-1s has been extensively reported, several questions remain; namely, whether tumor alarmins drive PD-1 expression and whether PD-1 intrinsically inhibits antitumor function of ILCs within the TME in the absence of exogenous IL-33. Similarly, expression of coinhibitory receptors such as CTLA4, TIGIT, LAG3, and TIM3 on ILCs is reported within the TME, but a functional role for these inhibitory receptors on ILC function is yet to be elucidated (19).

In this study, we sought to investigate whether PD-1 regulated ILC subsets in the TME, in the absence of additional exogenous IL-33. Using an unbiased scRNA-seq approach, we found that there was an increase in NK1.1^−^Tbet^+^ cells within the TME that was controlled by PD-1. This population, despite expressing type 1 signature of ILCs, did not express NKp46 or NK1.1 proteins as measured by AbSeq antibodies. We found that the NK1.1^−^Tbet^+^ population could be identified in both WT and PD-1-deficient mice in steady state. In tumors, PD-1 deficiency resulted in a significant increase in the frequency of NK1.1^−^Tbet^+^ cells. We found this subset up-regulated PD-1 in the presence of tumor-derived lactate and PD-1 upregulation significantly down-regulated the mammalian target of rapamycin (mTOR)-mediated proliferation of this subset within the TME. Taken together, this work defines a subset of NK1.1^−^Tbet^+^ ILCs within the TME that is regulated by PD-1.

## Results

### PD-1 Expression Is Noted within T-bet^+^ ILCs Subpopulations in Tumor.

**Fig. 1.**
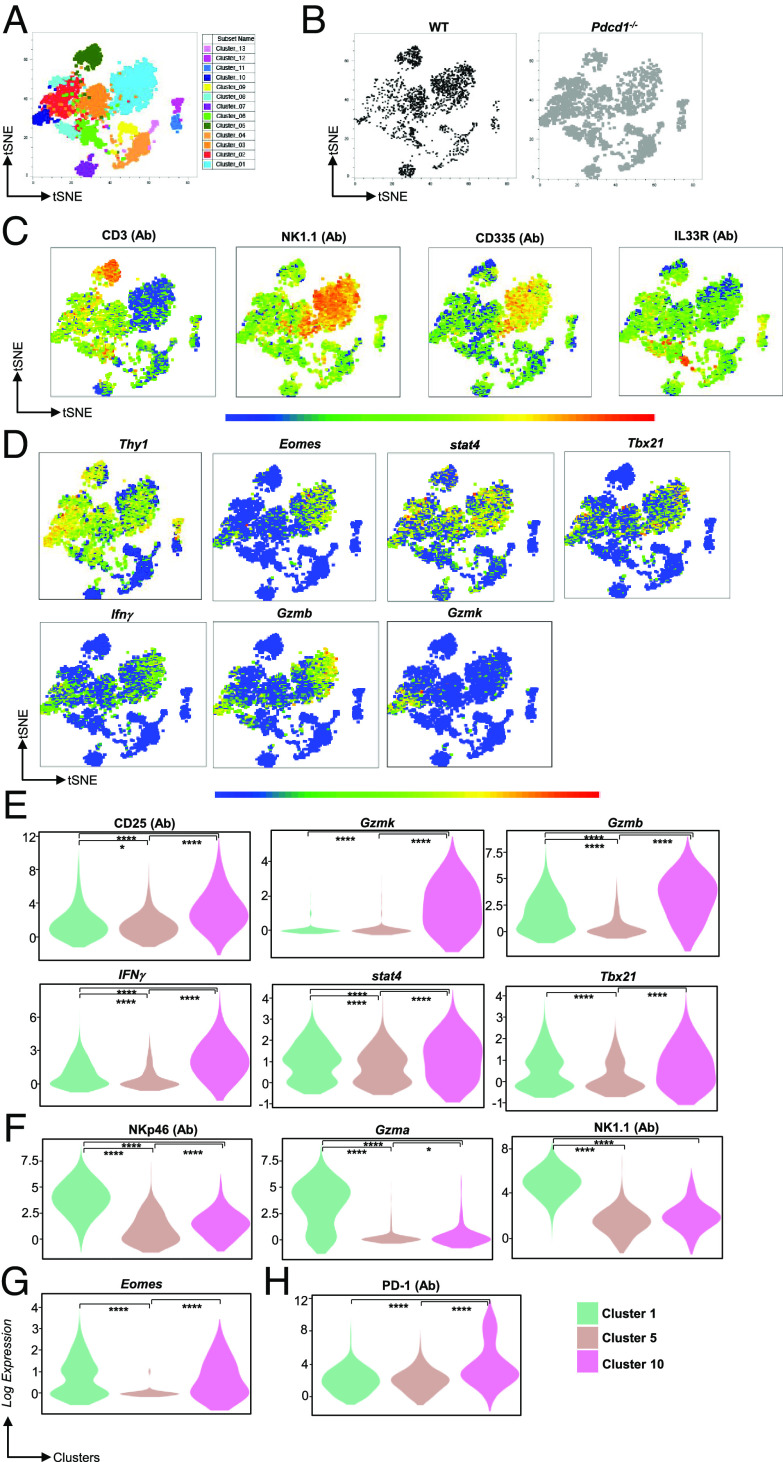
Single-cell sequencing analysis reveals PD-1 expression in Tbet^+^ ILCs within the tumor microenvironment. C57BL6 WT and *pdcd1^−/−^*mice were reconstituted with B16F10 melanoma cells via subcutaneous injection. Tumor-infiltrating lymphocytes were subjected to single-cell analysis. Analysis was performed with SeqGeq software with specific plugins used for gene expression analysis. Cluster analysis was performed using the phenograph plugin, and the various populations are highlighted *A*, both WT and PD-1 KO samples were then decoupled to show similar clustering between the samples *B*. tSNE plots of CD3, NK1.1, NKp46, and IL33R is shown *C*. tSNE analysis of type 1 gene transcripts within the various clusters is shown in *D*. Violin plots were generated using the violin plot plugin between T cell, NK cell, and cluster 10 cells and significant protein and gene transcript changes are shown in *E*–*H*. Data shown are form n = 5 WT and n = 5 KO mice. Statistical analysis within violin plots were Kruskal–Wallis test.

The role of PD-1 in exogenous IL-33-driven ILC-2 antitumor responses has been reported in models of pancreatic cancer (25) and in metastatic melanoma (36), but whether PD-1 function is relevant in the absence of the addition of exogenous IL-33 is unknown. Here, we sought to determine ILC subsets within TME that is controlled by PD-1. WT or *Pdcd1^−/−^* mice were engrafted with B16F10 melanoma cells (B16), and the tumor growth was monitored (*SI Appendix,* Fig. S1*A*). Tumors were harvested at day 12 post inoculation, and tumor-derived lymphocytes were isolated. Using an unbiased approach, we investigated the distribution of Lineage^-^Thy1^+^ subsets and their relative PD-1 expression within the TME of B16-BL6 murine melanomas. For this purpose, we combined PD-1 protein expression with transcriptomic analysis using the BD Rhapsody platform. We subjected enriched Lineage^−^Thy1^+^ ILCs from the TME (n = 5 WT and n = 5 KO mice) to single-cell analysis. Initial clustering analysis identified 13 clusters within the enriched TILs (Fig. 1*A*). First, we investigated whether the TILs clustered in a similar fashion in the WT and PD-1 knockout mice. On comparing WT and *Pdcd1^−/−^*TIL populations, both WT and *Pdcd1^−/−^* TIL populations clustered in a similar fashion but within the KO, an increase in cell numbers was noted within certain clusters (Fig. 1*B*). The differential gene expression patterns and pathway analysis of the 13 clusters was performed to identify immune populations that occupy the TME (Datasets S1 and S2 and *SI Appendix*, Figs. S2 and S3). Differential gene expression analysis identified cluster 5 and cluster 1 as predominantly expressing CD3 and NK1.1 protein (Abseq). Of note, cluster 10 possessed a type 1 phenotype but did not express NK1.1 (AbSeq) or NKp46 (AbSeq) protein on the surface (Fig. 1*C*). We next investigated the transcriptomic profile of cluster 10 and found that cluster 10 was enriched in type 1 genes such as *T-bet (Tbx21), Ifnγ, and Stat4* as compared to clusters 1 and 5. Of note, cluster 10 also showed cytotoxic potential identified through *Gzmb* and *Gzmk* gene transcripts (Fig. 1*D*). Finally, we performed quantitative analysis of proteins and transcript expression between clusters 1, 5, and 10 which identified a significant increase in the expression of CD25 (Abseq), *Gzmk*, *Tbx21*, *Stat4*, *Ifnγ*, and *Gzmb*, in cluster 10 as compared to cluster 1 (NK1.1^+^ cluster; Fig. 1*E*) and/or cluster 5 (CD3^+^ cluster). Cluster 10 expressed significantly low CD335 (Nkp46), *Gzma*, and NK1.1 (Fig. 1*F*). No difference in *Eomes* transcript expression was noted between clusters 1 and 10 (Fig. 1*G*). Of note, PD-1 protein expression was noted in cluster 10 suggesting that PD-1 may control this population of ILCs within the TME (Fig. 1*H*). Finally, we investigated whether the NK1.1^−^Tbet^+^ population of ILCs existed under normal homeostatic conditions or whether NK1.1 was down-regulated within TILs. Indeed, our single-cell Abseq demonstrated that NK1.1 was expressed on TILs and was not down-regulated. However, to confirm this finding, we determined the existence of this population in nontumor mice. We found that WT mice possessed a similar immune cell subset within the bone marrow, liver, and spleen. These cells did not express NK1.1 and were Tbet^+^RORγt^−^NKp46^−^ (*SI Appendix,* Fig. S1 *B*–*K*). On identifying this population in WT mice, we next investigated the frequency of these subsets under homeostatic conditions in PD1 knockout mice. Our data demonstrate that this population occurs both in WT and PD-1 knockout mice under steady state (*SI Appendix,* Fig. S1 *B*–*K*).

### PD-1 Deficiency Specifically Increases T-bet^+^NK1.1^−^ ILCs within the TME but Not in Other Target Tissues.

**Fig. 2.**
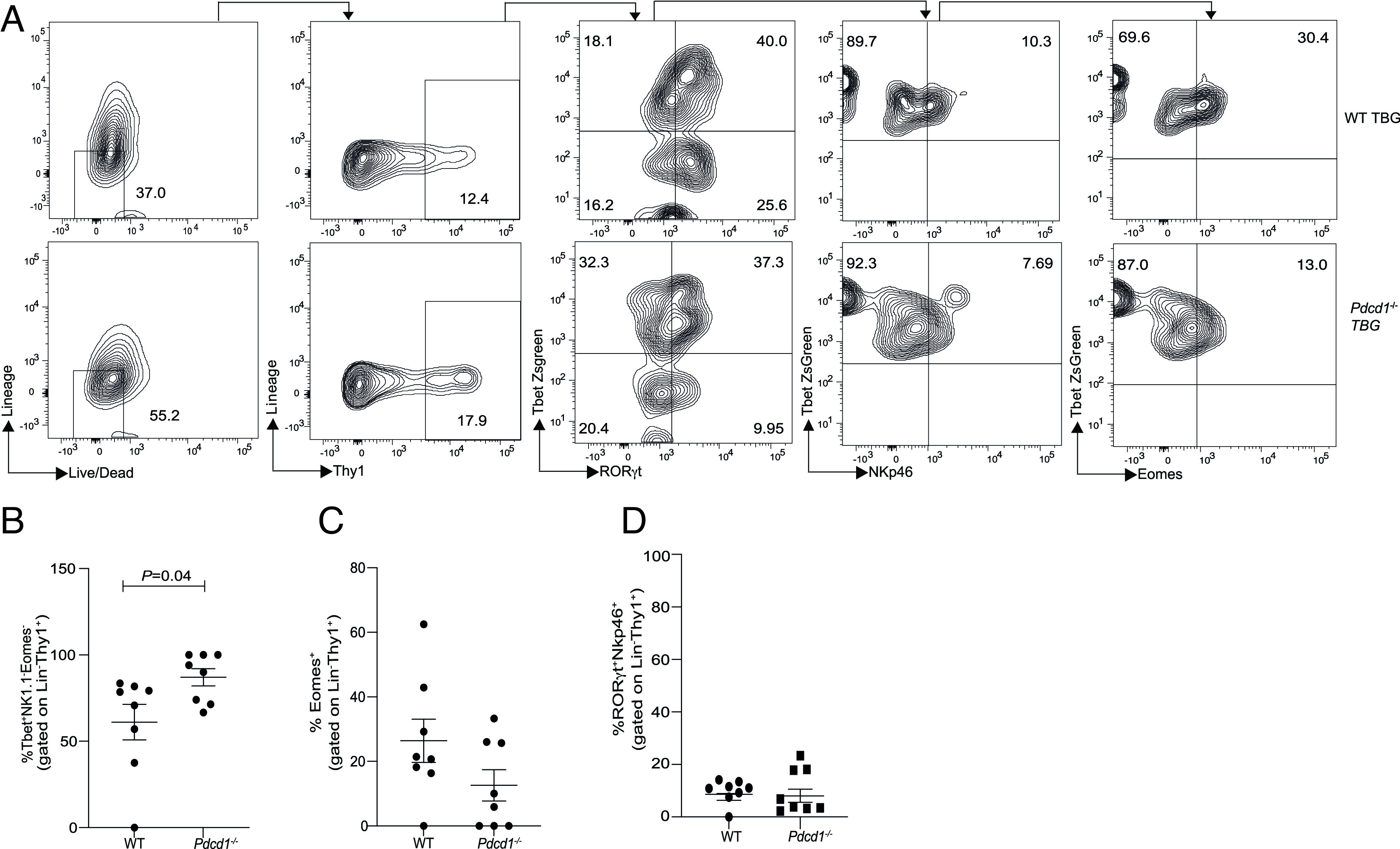
PD-1 deficiency significantly increases Tbet^+^RORγt^−^Eomes^−^NK1.1^−^ ILCs within the B16 TME. C57BL6 WT *TbetZsGreen* mice (WT TBG) or *Pdcd1^−/−^TbetZsGreen* mice (*Pdcd1*^−/−^TBG) were reconstituted with B16F10 melanoma cells via subcutaneous injection. Tumor-infiltrating lymphocytes were characterized by flow cytometry. Representative flow cytometry plot and gating strategy is shown in *A*. Summary of the frequency of NK1.1^−^Tbet^+^Eomes^−^ILCs in WT and *Pdcd1*^−/−^ mice within the tumor infiltrating lymphocytes is shown in *B*. Frequency of Eomes^+^ ILCs *C* and NKp46^+^ ILCs *D* is shown. Data shown are Mean ± SEM, each data point refers to the number of mice per cohort used per experiment, statistical significance was performed using an unpaired *t* test. In vivo experiments were repeated at least twice.

To further characterize the Tbet^+^ NK1.1^−^ ILCs subset, we generated *Pdcd1^−/−^Tbet-ZsGreen* mice. WT *Tbet-ZsGreen* and *Pdcd1^−/−^Tbet-ZsGreen* mice were reconstituted with B16 melanoma and tumor growth was monitored (*SI Appendix,* Fig. S4 *A*–*C*) which showed a significant decrease in tumor growth in the *Pdcd1^−/−^Tbet-ZsGreen* mice. In subsequent experiments, tumors were resected at day 14, and then, ILC subsets were determined within the TME. The expression of PD-1 in Tbet^+^NK1.1^−^ ILCs and ILC-2s were first measured by flow cytometry within TILs (*SI Appendix,* Fig. S4 *D* and *E*). In line with previous observation, PD-1 expression was noted in ILC-2s. Of significance was the expression of PD-1 on Tbet^+^NK1.1^−^ ILCs. Therefore, we investigated which Tbet^+^NK1.1^−^ cells (Eomes^+/−^) were controlled by PD-1 within the TME. In the absence of PD-1, a substantial increase in T-bet^+^ cell frequency was noted in the NK1.1^−^RORγt^−^ ILC subset within the TME (Fig. 2*A*). On cumulative analysis, we found that PD-1 specifically regulated the frequency of Tbet^+^Eomes^−^RORγt^−^ (i.e., Tbet^+^NK1.1^−^ ILC) subset within the TME (Fig. 2 *B*–*D*) but such regulation was not apparent in the small intestine and skin but a significant difference was noted in the lungs (*SI Appendix,* Fig. S4 *F*–*H*). Within the secondary lymphoid organs, again PD-1 deficiency was associated with no significant increase in the T-bet^+^ subset in the spleen but a significant increase was observed within the tumor-draining lymph nodes (*SI Appendix,* Fig. S4 *I* and *J*). These data suggest that PD-1 specifically regulates T-bet^+^ ILCs within the melanoma TME and the associated draining lymph nodes.

### PD-1 Controls IFNγ Production in Tbet^+^NK1.1^−^ ILCs within the TME.

**Fig. 3.**
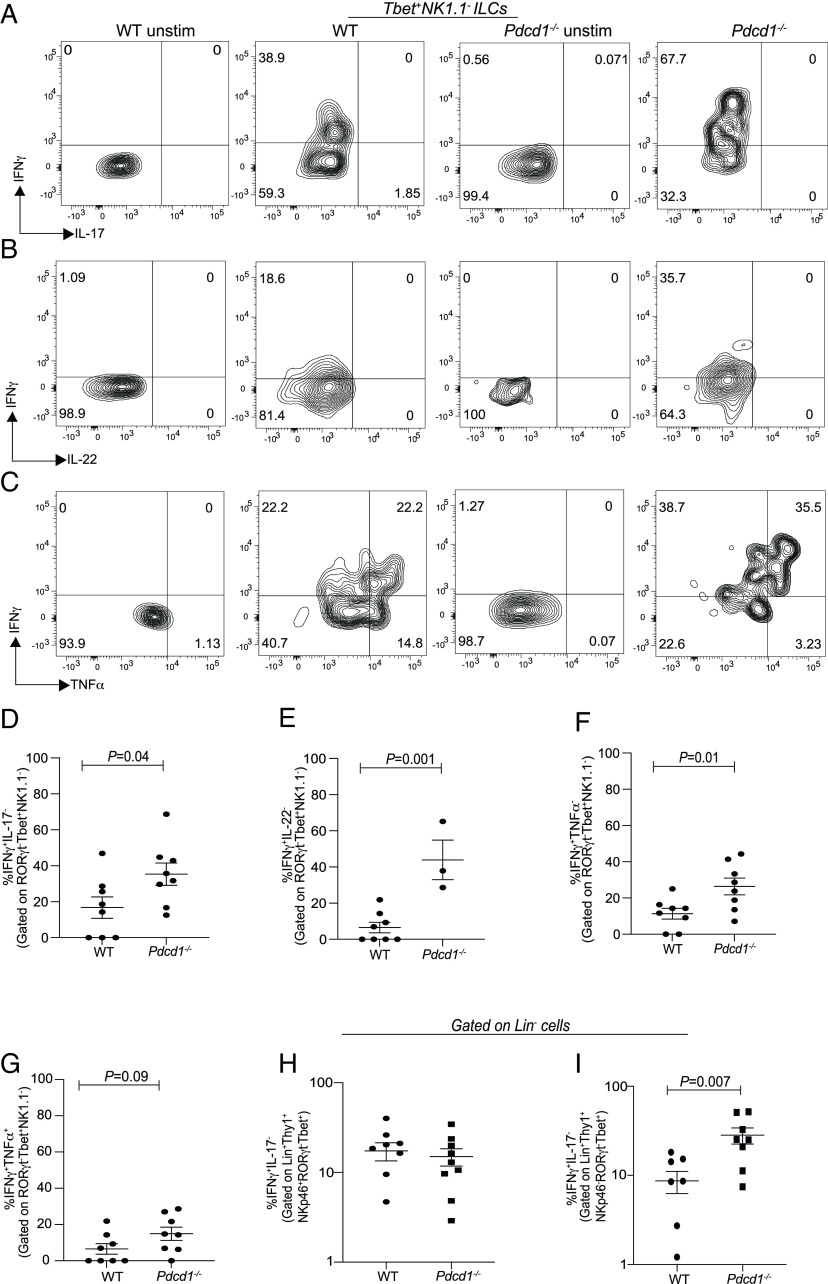
PD-1 deficiency significantly increases IFNγ^+^Tbet^+^ILCs within the B16 melanoma TME. C57BL6 WT TBG or *Pdcd1*^−/−^TBG were reconstituted with B16F10 melanoma cells via subcutaneous injection. Tumor infiltrating lymphocytes were isolated, stimulated for 4 h in the presence of cytokine stimulation cocktail and then characterized by flow cytometry. ILCs were characterized as Lineage^−^Thy1^+^. Representative flow plots and summary data for IFNγ and IL-17 expression in WT TBG and *Pdcd1*^−/−^TBG (*A* and *D*). Representative flow plots and summary data for IFNγ and IL-22 expression in WT TBG and *Pdcd1*^−/−^TBG (*B* and *E*). Representative flow plots and summary data for IFNγ and TNFa expression in WT TBG and *Pdcd1*^−/−^TBG (*C*, *F*, and *G*). The expression of IFNγ in Lineage^+^NKp46^+^ TILs (*H*) and Lineage^+^NKp46^−^ TILs are shown *I*. Data shown are Mean ± SEM, each data point refers to the number of mice per cohort used per experiment, and statistical significance was performed using a unpaired *t* test. In vivo experiments were repeated at least twice.

Previously, we found that PD-1 controlled ILC-2 cytokine secretion by regulating STAT5 (32). We sought to determine whether PD-1 had a similar function in tumor-derived T-bet^+^ ILCs. For these experiments, since an increase in Th-bet^+^ ILCs was noted, the cytokine potential of these cells within the TME was tested. Flow cytometry data suggested that in the absence of PD-1, a significant increase in IFNγ production was noted within the T-bet^+^ ILCs (Fig. 3 *A*–*D*), but these cells did not show increased production of IL-17 or IL-22 or TNF-a (Fig. 3 *A*, *B*, and *E*–*G*). Next, it was determined whether the changes seen in ILC-driven IFNγ production were also reproduced within the Lineage^+^NKp46^+^ population within the TME. Since the lineage panel included NK1.1 and CD49b, any NKp46^+^ population may have reflected the regulation of NK1.1^+^ cells within the lineage gate. No difference was noted in IFNγ production within the NKp46^+^ Lineage^+^ gate (Fig. 3*H*). However, significant IFNγ production was noted within the Lineage^+^ gate originating from cells that are NKp46^−^ which may include T cells within the TME (Fig. 3*I*). Taken together, the data suggest that within the ILCs in the TME, PD-1 regulates cytokine production by Tbet^+^ ILC populations that possess a helper-1 like phenotype.

### PD-1 Controls Proliferation of Tbet^+^NK1.1^−^ ILCs within the TME.

**Fig. 4.**
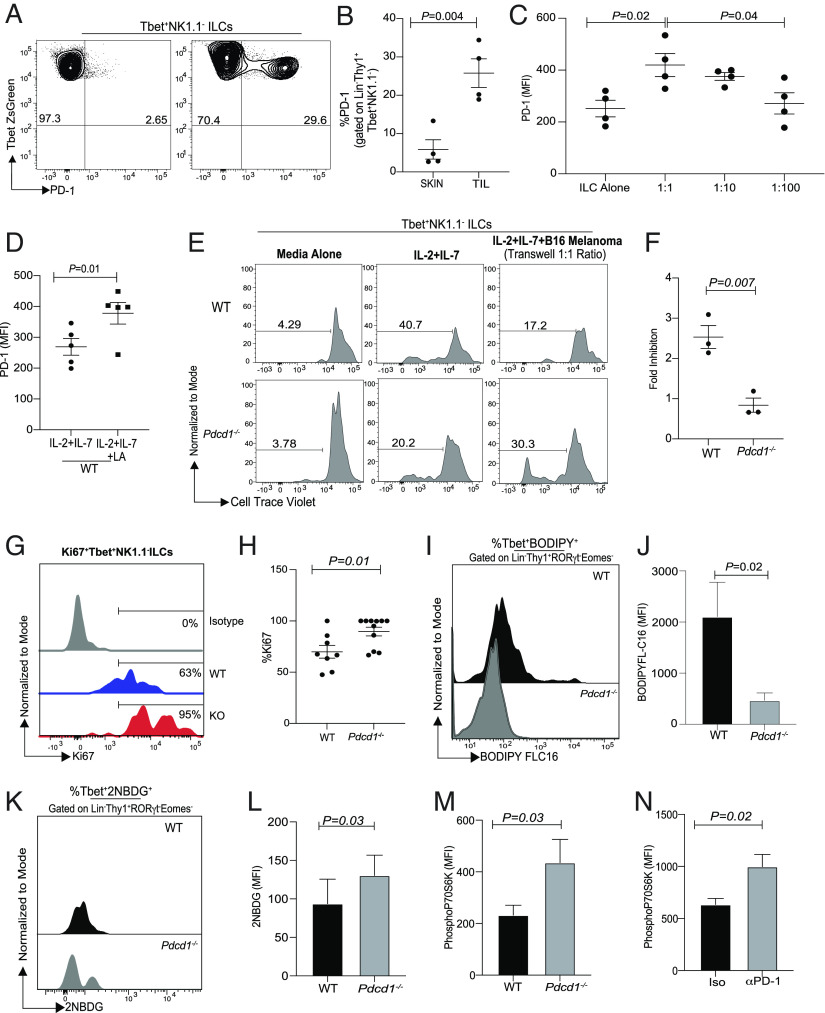
PD-1 inhibits the proliferation of Tbet^+^NK1.1^−^ ILCs within the B16 TME. C57BL6 WT TBG or *Pdcd1*^−/−^TBG were reconstituted with B16F10 melanoma cells via subcutaneous injection. At day 12, Tbet^+^NK1.1^−^ ILCs were characterized by flow cytometry. PD-1 expression was measured in skin-derived and tumor-derived Tbet^+^RORγt^−^ NK1.1^−^ ILCs (*A* and *B*). Next, PD-1 expression was monitored in cultures stimulated with either cytokines alone (IL-2, IL-7) or in the presence of B16 melanoma cell line at 1:1, 1:10 (1 melanoma cell:10 lymphocytes), and 1:100 ratio after 6 h (*C*). Splenocytes were incubated for 24 h with IL-2 and IL-7 alone or with lactic acid and then PD-1 expression on Tbet^+^ILCs were measured (*D*). Representative flow plots showing proliferation of Tbet^+^RORγt^−^NK1.1^−^ ILCs from WT and *Pdcd1*^−/−^ cultures in transwell experiments are shown *E*. Fold inhibition of proliferation in the presence of melanoma supernatant in WT and *Pdcd1*^−/−^Tbet^+^NK1.1^−^ ILCs is shown *F*. Fold Inhibition was measured as follows. WT ILC proliferation in response to IL-2 and IL-7 was measured and then the rate of proliferation with B16 melanoma supernatant was measured. Fold inhibition between the two culture conditions was calculated and then plotted for WT and KO. Representative flow cytometry showing Ki67 expression in Tbet^+^RORγt^−^NK1.1^−^ tumor-derived ILCs from WT and *Pdcd1*^−/−^ cohorts (*G*) and cumulative data shown in *H*. BODIPY (4,4-difluoro-5,7-dimethyl-4-bora-3a,4a-diaza-s-indacene-3-hexadecanoic acid) MFI is shown in *I* and *J*, (n = 5, paired one-tailed *t* test); 2NBDG uptake is shown in *K* and *L*, (n = 4, paired one-tailed *t* test). TILs were harvested and stimulated with IL-2 and IL-7 for 15 min and then phosphoP70S6Kinase was measured (*M*), (n = 4, paired one-tailed student *t* test). WT TILs were stimulated for 3 d with either isotype control or anti-PD-1 antibody. At day 3, TILs were stimulated for 15 min with IL-2 and IL-7, and then phosphoP70S6Kinase was measured (*N*) (n = 3, paired one-tailed student *t* test). Data shown are Mean ± SEM, each data point refers to the number of mice per cohort used per experiment. In vivo experiments were repeated twice with n = 4 to 5 animals per cohort; in-vitro experiments were repeated three times. An unpaired *t* test was performed for comparison of two groups and an ANOVA was performed for comparison of three or more groups.

The molecular mechanism by which PD-1 regulated the frequency of Tbet^+^NK1.1^−^ ILCs within the TME was next determined. First, the PD-1 expression pattern in T-bet^+^ ILCs within the TME and normal surrounding skin was evaluated. T-bet^+^ ILCs within the TME significantly up-regulated PD-1 expression when compared to their counterparts within normal skin (Fig. 4 *A* and *B*). This suggested that PD-1 was selectively enhanced within the TME Tbet^+^NK1.1^−^ ILCs. Next, the ability of tumor cells to induce PD-1 on T-bet^+^ ILCs was evaluated. In transwell experiments, B16 melanoma tumor cells cocultured in transwell plates with ILCs induced PD-1 with an increase in frequency in PD-1^+^ ILCs observed at the 1:1 ratio at 6 h, with a trend noted at 1:10 ratio which was completely abrogated at a 1:100 ratio (Fig. 4*C* and *SI Appendix,* Fig. S5*A*). Since tumor-derived lactate can suppress proliferation of ILC-2s (24), it was next investigated whether lactic acid was produced by B16 tumors and whether lactic acid induced PD-1 on Tbet^+^ILCs. In in vitro experiments, tumor supernatants contained significant amounts of lactic acid (*SI Appendix,* Fig. S5*B*) and lactic acid addition significantly increased PD-1 expression on Tbet^+^ILCs (Fig. 4*D* and *SI Appendix,* Fig. S5 *C* and *D*).

We next investigated whether tumor cell supernatant affected the proliferative capacity of ILCs through PD-1. We measured the ability of WT Tbet^+^NK1.1^−^ ILC proliferation in the presence of tumor cell supernatant and then compared the relative proliferation of *Pdcd1^−/−^* T-bet^+^ ILCs. We found that B16 tumor supernatant significantly inhibited proliferation of WT Tbet^+^NK1.1^−^ ILC as compared to PD-1-deficient cells (Fig. 4 *E* and *F*). In fresh cell culture media, *Pdcd1^−/−^*T-bet^+^ ILCs had reduced proliferation as compared to WT cells in response to IL-2 and IL-7. Furthermore, within the B16 TME in vivo, a significant increase in the proliferative potential of T-bet^+^ ILCs (as measured by Ki67 staining) was noted in the *Pdcd1^−/−^*cohorts (Fig. 4 *G* and *H*). Subsequently, we tested the molecular mechanism by which PD-1 suppressed proliferation within ILC subsets. Recent studies have explored the role of PD-1 in altering the metabolic phenotype of T cells (38) and myeloid cells (39). Specifically, metabolic regulation of myeloid cells within the TME by PD-1 has been shown to enhance antitumor responses. PD-1 has also been implicated in regulating ILC-2 metabolism in allergic inflammation (40). In line with previous observations (40), PD-1 significantly regulated fatty acid metabolism and glycolysis in WT mice (Fig. 4 *I*–*L*). We next investigated whether a change in metabolic signatures could be identified at a single-cell resolution within the WT and PD-1 KO ILC populations within the TME. We found that the PD-1 KO type I ILCs from cluster 10 had significantly decreased *apoe* gene transcript as compared to the WT cohorts (*SI Appendix,* Fig. S5*E*). In addition to analyzing the transcript of *apoe* from WT and KO samples from cluster 10, we also performed an additional analysis on PD-1^+^ and PD-1^−^ cells from cluster 10. In this analysis, we again found that *apo*e expression was limited to PD-1^+^ cells (*SI Appendix,* Fig. S5*F*). In keeping with increased glycolysis, PD-1 deficiency also increased mTOR signaling in T-bet^+^ ILC TILs within B16 TME as identified by phosphorylated p70S6K (Fig. 4*M* and *SI Appendix,* Fig. S5*G*). The molecular mechanism was confirmed in WT T-bet^+^ ILC TILs whereby PD-1 blockade enhanced mTOR signaling and p70S6K phosphorylation (Fig. 4*N* and *SI Appendix,* Fig. S5*H*). Taken together, we propose the following molecular mechanism by which PD-1 regulates ILCs within the TME. Depending on the active alarmins within the TME, PD-1 expression is increased in the corresponding ILC subset (exogenous IL-33 in the case of ILC-2s, and melanoma tumor cell-derived lactate in case of T-bet^+^ ILCs). Blocking PD-1 enhances the proliferative capacity of the ILC subsets within the TME by enhancing glycolysis and up-regulating the mTOR signaling pathway.

### PD-1 Regulates Tbet^+^NK1.1^−^ ILCs within the TME in AOM-dextran sodium sulphate (DSS)-Induced Colorectal Cancer (CRC).

**Fig. 5.**
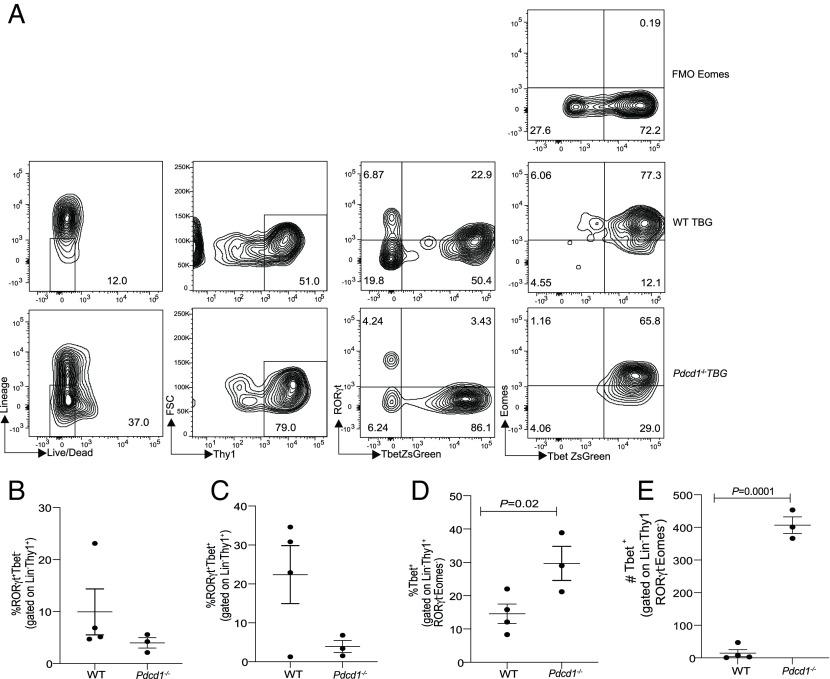
PD-1 regulates Tbet^+^RORγt^−^ ILCs within the colorectal TME. C57BL6 WT TBG or *Pdcd1*^−/−^TBG were treated with one dose of AOM followed by three cycles of DSS. Tumors were resected, and ILC frequency was measured by flow cytometry. Representative flow cytometry is shown in *A* and cumulative frequency and absolute numbers are shown in *B*–*E*. Data shown are Mean + SEM, each data point refers to the number of mice per cohort used per experiment. In vivo experiments were repeated twice with n = 4 to 5 animals per cohort. An unpaired *t* test was performed.

To test the reproducibility of our observation within the orthotopic melanoma model, we next investigated whether PD-1 controlled ILCs within the TME in an inducible model of cancer within a different tissue. A model of CRC was chosen since it has been shown that ILCs support CRC growth via IL-22 and we wondered whether within this model, PD-1 can increase Tbet^+^NK1.1^−^ ILCs with a type 1 phenotype. In AOM-DSS, PD-1 mice were significantly more susceptible to DSS-mediated weight loss during the first treatment cycle, but susceptibility was reduced in the third treatment cycle as compared to WT cohorts (*SI Appendix,* Fig. S6*A*). In addition, there was significantly lower number of tumors noted within the intestine of *Pdcd1^−/−^* mice (*SI Appendix,* Fig. S6*B*) along with an increase in inflammation within the intestine (as measured by the intestinal length, with reduced length associated with greater inflammation; *SI Appendix,* Fig. S6*C*). We next harvested the tumors to determine the frequency of ILCs. Consistent with our melanoma data, we found a significant increase in Tbet^+^NK1.1^−^ ILCs frequency and absolute numbers within the tumors (Fig. 5 *A*–*E*). These data suggest that PD-1 deficiency can enhance Tbet^+^NK1.1^−^ ILCs within CRC.

### Blocking Antibodies to PD-1 Significantly Increases Tbet^+^NK1.1^-^ ILCs in Subcutaneous and Metastatic B16 Melanoma.

**Fig. 6.**
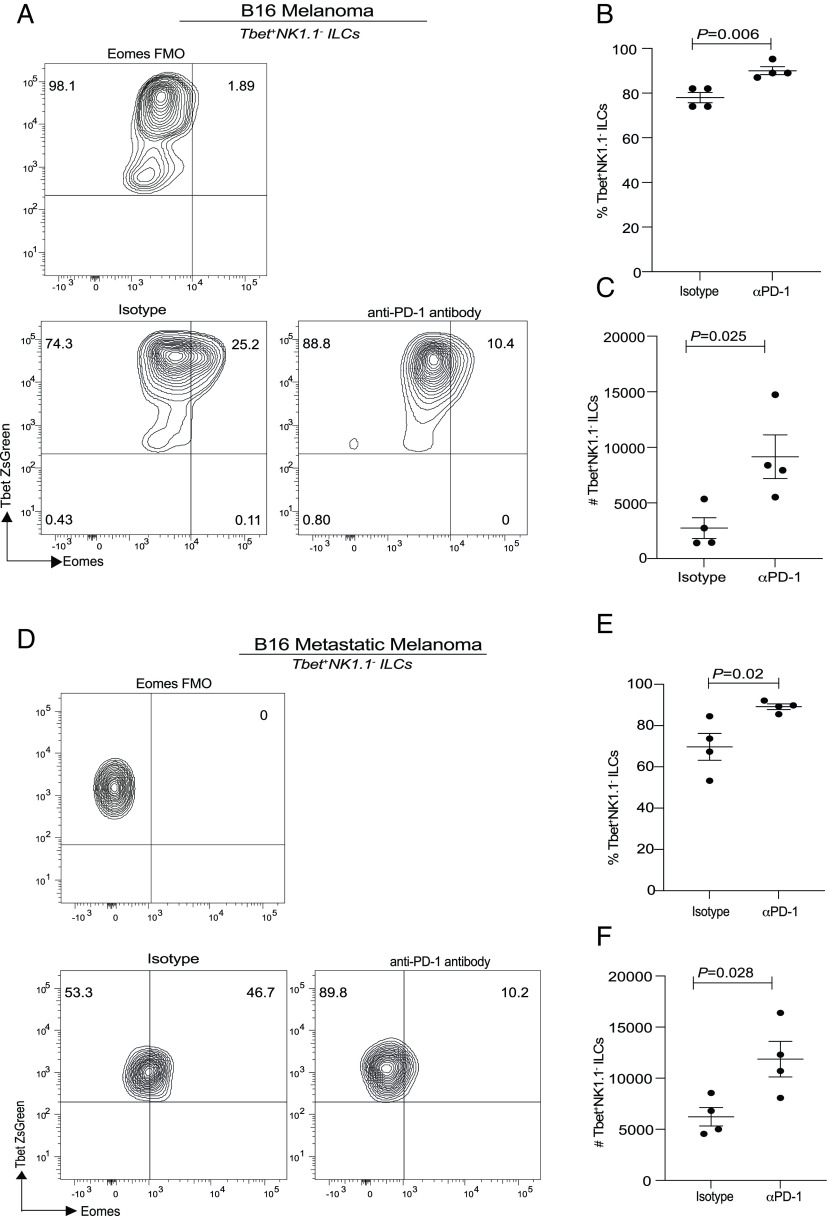
PD-1 blocking antibodies significantly increases tumor-derived Tbet^+^RORγt^−^ ILCs ILCs in subcutaneous melanoma and metastatic melanoma murine models. C57BL6 WT TBG mice were reconstituted with B16F10 melanoma cells via subcutaneous injection. Tbet^+^ILCs were characterized as Lineage^−^Thy1^+^RORγt^−^NKp46^−^ cells. Representative flow plot depicting Tbet^+^ ILCs (*A*) and cumulative data from B16 melanoma TILs (*B* and *C*). C57BL6 TbetZsGreen mice were reconstituted with B16F10 melanoma cells via intravenous injection. Data from metastatic melanoma TILs are shown in *D*–*F*. Data shown are Mean ± SEM, each data point refers to the number of mice per cohort used per experiment. In vivo experiments were repeated twice with n = 4 to 5 animals per cohort. An unpaired *t* test was performed.

We next tested whether the phenomenon in the PD-1 KO mice can be reproduced in a therapeutic context. Since an increase in Tbet^+^NK1.1^−^ ILCs was noted both in the TME and lungs (*SI Appendix,* Fig. S4*F*), we tested whether these ILC subsets were increased in both subcutaneous and metastatic melanoma model. WT mice were subcutaneously reconstituted with B16 cells, and then tumors were allowed to engraft. Mice were treated with either isotype or anti-PD-1 at days 7, 9, 11, and 12, and the tumor growth curve was monitored (*SI Appendix,* Fig. S6*D*). Mice were killed on day 14, and then, ILC subset frequency was analyzed. A significant increase in Tbet^+^NK1.1^−^ ILCs frequency and absolute numbers was noted in the anti-PD-1-treated cohorts as compared to the control isotype treatment cohorts (Fig. 6 *A*–*C*). We next tested the frequency of these cells in a metastatic model of melanoma and found a similar increase in frequency and absolute numbers of Tbet^+^NK1.1^−^ ILCs (Fig. 6 *D*–*F*). These data suggest that anti-PD-1 can significantly increase Tbet^+^NK1.1^−^ ILCs both within the subcutaneous and metastatic TME.

### PD-1 Blockade Partially Protects Murine Recipients from Tumor Burden by Enhancing Helper ILCs in the Absence of NK Cells and Adaptive Immune Cells.

**Fig. 7.**
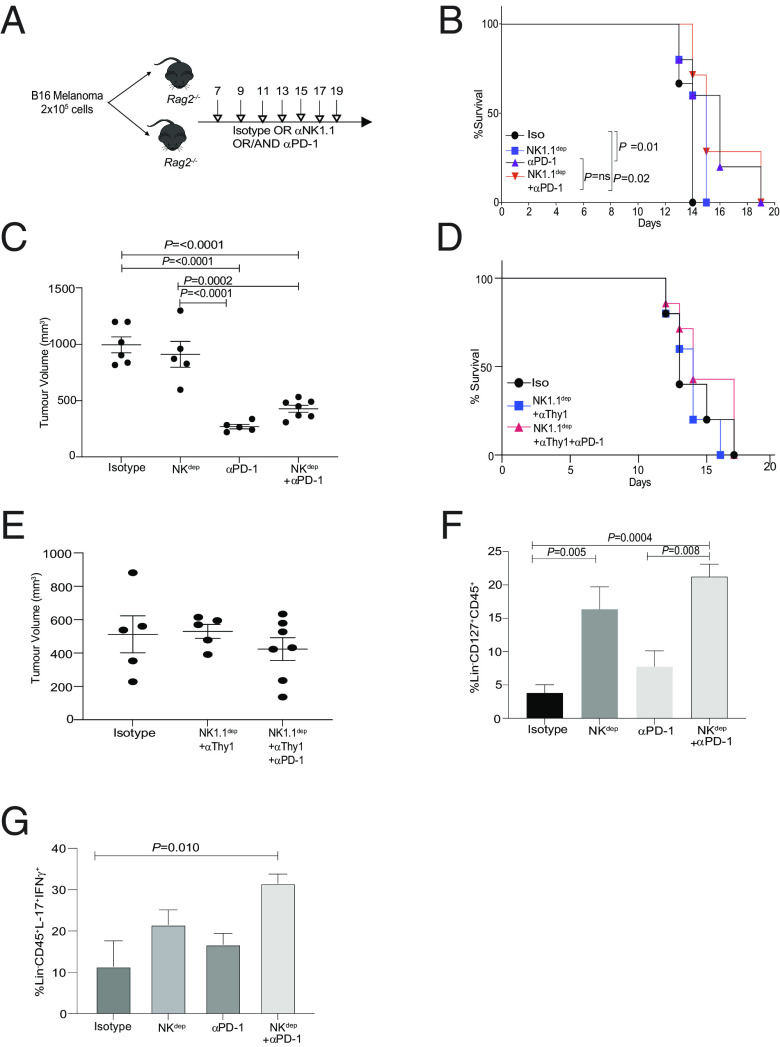
PD-1 blockade enhances the survival of tumor bearing *Rag* mice. B6 *Rag*^−/−^ mice were reconstituted with B16F10 cells via subcutaneous injection. Mice were treated with either isotype control, NK depleting antibody (NK1.1) alone, anti-PD-1 alone, or in combination with anti-PD-1 antibody (*A*). Survival of mice in the different cohorts were monitored over a period of 20 d (*B*). The experiment was repeated in mice and tumor volume was shown at day 14 in *C*. B6 *Rag*^−/−^ mice were reconstituted with B16F10 cells via subcutaneous injection. Mice were then treated with either isotype control, NK-depleting antibody (NK1.1), and ILC-depleting antibody (Thy1) alone or in combination with anti-PD-1 antibody. Survival of mice in the different cohorts were monitored (*D*) and tumor volume was shown at day 14 (*E*). Experimental plan in *A* was repeated. At day 12, tumors were resected and frequency of helper ILCs in shown in *F*. TILs were stimulated with cytokine stimulation cocktail for 4 h. Cells were then subjected to intracellular flow cytometry to measure effector cytokines. Frequency of IFNγ and IL-17-positive ILC TILs (*G*). Data shown are Mean ± SEM, each data point refers to the number of mice per cohort used per experiment. In vivo experiments were repeated twice with n = 4 to 5 animals per cohort. A one-way ANOVA was performed to measured differences between the various cohorts. A Kaplan–Meier survival curve with a log rank test was performed for significance between cohorts.

Recent reports have shown that PD-1 blockade can enhance antitumor immunity by specifically targeting the innate immune cells such as macrophages in the TME (39). Therefore, we investigated the contribution of ILCs versus macrophages and NK1.1^+^ cells in eliciting antitumor responses in the presence of PD-1 blockade. NK1.1^+^ cell-depleted *Rag^−/−^* host mice were injected with B16 melanoma cells. On tumor establishment, animals were repeatedly injected with either isotype control or NK1.1^+^ cell-depleting antibodies with or without anti-PD-1 or anti-PD1 alone (Fig. 7*A* and *SI Appendix,* Fig. S7*A*). Within this experimental setup, we found that blocking PD-1 partly enhanced the survival of mice, both within the NK1.1^+^ depleted and NK1.1^+^ replete animal cohorts (Fig. 7*B*). In addition to increased survival, a significant decrease in tumor volume was also noted in the murine recipients that received PD-1 immunotherapy (Fig. 7*C* and *SI Appendix,* Fig. S7*A*). We found that the treatment of anti-PD-1 significantly reduced tumor volume in mice compared with animals that were not treated with anti-PD-1. Of note, in the anti-PD-1 treated animal groups, depletion of NK1.1^+^ cells was associated with an incremental increase in tumor volume, but this difference was not significant suggesting an antitumor role for type 1 ILCs (*SI Appendix,* Fig. S7*A* and Dataset S3). By contrast, using a similar experimental setup, depleting NK cells and ILCs (using Thy1 antibody) abolished any protective tumor effects rendered by PD-1 blocking in these murine recipients (Fig. 7 *D* and *E* and *SI Appendix,* Fig. S7*B*). It is worth noting that the depletion of NK cells and ILCs continued after the establishment of tumors as these cells may play a significant role at this phase of tumor progression.

We next determined the immune cell subsets that are enhanced within the melanoma TME in the absence of NK cells and adaptive immune cells. We found depleting NK cells significantly enhanced IL-7R^+^ (CD127^+^) ILCs within the TME (Fig. 7*F* and *SI Appendix,* Fig. S7*C*). On PD-1 blockade, the frequency of IL-7R^+^ ILCs within the TME was further enhanced (Fig. 7*F*). Within this experimental setup, no difference in the frequency of myeloid and dendritic cell (DC) compartment was observed (*SI Appendix,* Fig. S7 *C*–*F*). In contrast to the myeloid and DC compartment, a significant increase in NK1.1^+^ cells within the lineage^+^ gate was noted on PD-1 blockade (*SI Appendix,* Fig. S7*G*). These data suggest that blocking PD-1 enhances antitumor responses in both the presence and absence of NK1.1^+^ cells with minimal contribution from the myeloid compartment.

We next investigated the cytokine profile of ILCs within the melanoma TME and found that blocking PD-1 significantly increased the frequency of ILCs that could express dual effector cytokines such as IL-17 and IFNγ (Fig. 7*G* and *SI Appendix,* Fig. S7*H*). In line with our previous observations (32), we found that the IL-13^+^ ILCs were enriched in the population of IFNγ^+^ cells within the TME (*SI Appendix,* Fig. S7 *H* and *I*). Taken together, our data suggest that PD-1 regulates IFNγ production and type 1 ILC phenotype within the melanoma TME.

### PD-1 Regulates Human T-bet^+^ILC Subsets in Cutaneous Melanoma and cSCC.

**Fig. 8.**
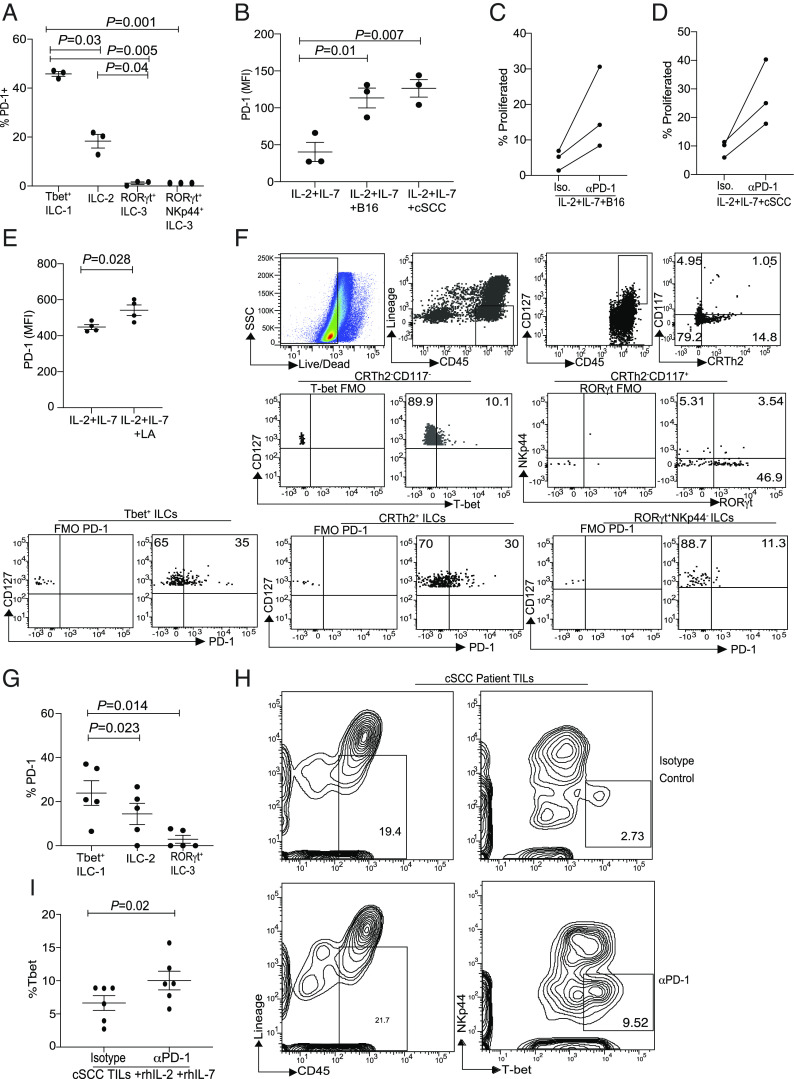
PD-1 regulates human Tbet^+^ ILC proliferation in human cSCC. Expression of PD-1 in various ILCs subsets from normal human donor peripheral blood mononuclear cells is shown *A*. Human melanoma and cutaneous squamous carcinoma cell lines were incubated at a 1:1 ratio in a 24-well transwell plate in the presence of normal donor PBMC. PD-1 protein expression within Tbet^+^ ILCs from the various culture conditions is shown in *B*. The rate of proliferation of Tbet^+^RORγt^−^ ILCs in the presence of melanoma either with isotype or anti-PD-1 antibody is shown in three donors (*C*), similarly the proliferation of Tbet^+^RORγt^−^ ILCs in the presence of cSCC either with isotype or anti-PD-1 antibody is shown in three donors (*D*). Human normal donor PBMC were stimulated with either IL-2 and IL-7 alone or in combination with LA and then PD-1 MFI in shown in Tbet^+^ILC (*E*). Tumor tissue were obtained from cSCC patients, and then, helper ILC subsets were characterized using flow cytometry. Representative flow plots showing ILC analysis in cSCC and summary of PD-1 expression is shown *F* and *G*. PD-1 expression was not done in RORγt^+^NKp44^+^ subset due to low frequency of the subset. TILs were expanded for 3 d in the presence of hIL-2, hIL-7 and either in the presence isotype antibody or anti-PD-1 antibody. Frequency of Tbet^+^ ILCs were measured by flow cytometry (*H* and *I*). Data shown are Mean ± SEM, each data point refers to an individual donor within the experiment. A one-way ANOVA was performed to measure difference between three or more cohorts and a paired *t* test for performed for data shown in *E* and *I*.

In recent years, blood and tumor ILC-1s have been defined in the literature based on transcript expression of *CD3D, CD3G, CD3E, CCL5, GMK, GZMM, GZMA, BCL11B, PRDM1, IKZF3, EOMES, and TBET* (41), along with protein expression consisting of IL-7R^pos^, c-KIT^neg^, and CRTH2^neg^ subsets (42). Within this definition, Qi et al. have identified an inhibitory ILC-1 population based on the expression of coinhibitory receptors, namely *TIGIT, CTLA4,* and *TNFRSF4* (42). Protein expression of TIGIT was then confirmed within the CD127^+^c-KIT^−^ CRTh2^−^ subset of ILC-1s, and this population of ILC-1s has been associated with poor CRC prognosis, but whether these TIGIT^+^ ILC-1 expressed T-BET protein was not verified. Similarly, in humans, we tested whether PD-1 played a similar role in regulating ILC populations. In humans, we classified the various ILC subsets as previously described (42–45). ILC-1s were classified as Lin^-^CD127^+^CD117^–^CRTH2^–^, ILC-2 as Lin^–^CD127^+^CRTH2^+^ and ILC-3 as Lin^–^CD127^+^CD117^+^CRTH2^-^RORγt^+^ and Lin^−^CD127^+^CD117^+^CRTH2^−^ RORγt^+^Nkp44^+^cells (*SI Appendix,* Fig. S8*A*). Next, consistent with our murine data, we excluded CD56 and CD5 in order to exclude any NK cells or ILC-3s or tumor-defined ILC-1s in cSCC (46) from our analysis. ILC-1 populations were further discriminated based on T-BET expression (*SI Appendix,* Fig. S8*B*) (41). Using this analysis, we found that PD-1 expression was significantly increased in the CD56^−^ CD5^−^CD127^+^CD117^−^CRTH2^-^T-BET^+^ subset of ILC (Fig. 8*A* and *SI Appendix,* Fig. S8*C*). We next investigated whether tumor supernatant can induce PD-1 within this T-BET^+^ ILC subset. For this experiment, we used human melanoma and cutaneous squamous cell carcinoma cell lines. Similar to murine experiments, PD-1 expression was significantly increased in the presence of melanoma and cSCC tumor supernatants (Fig. 8*B* and *SI Appendix,* Fig. S8*D*). Next, we investigated whether this increase had a functional impact on ILC proliferation in keeping with the murine observations. We found that similar to murine studies, an increase in T-BET^+^ ILC proliferation was noted on blocking PD-1 in normal human donors (Fig. 8 *C* and *D* and *SI Appendix,* Fig. S8*E*). The role of lactate in inducing PD-1 within human T-BET^+^ ILCs was then evaluated. We found that like murine T-bet^+^ ILCs, PD-1 was up-regulated by lactic acid within normal human T-BET^+^ ILCs (Fig. 8*E* and *SI Appendix,* Fig. S8*F*). We next tested whether our observation on human ILCs with cSCC cell lines was relevant in primary tumors of human cSCC. A comprehensive atlas of human cSCC has been published, along with the report that ILCs can populate cSCC tumors (46, 47). However, whether the PD-1^+^ T-BET^+^ ILCs exist and elicit functional antitumor responses within cSCC is unknown. Moreover, whether PD-1 restricts ILC antitumor responses in human cSCC is unclear. Our data confirm that helper ILCs are present within human cSCC TME (Fig. 8 *F* and *G*). Out of these various populations, high PD-1 expression was associated with T-BET^+^ ILCs with expression also noted in ILC-2s with minimal expression in RORγt^+^ ILCs within TILs and PBMC (Fig. 8*G* and *SI Appendix,* Fig. S8*G*). Next, to demonstrate functional significance of PD-1 on ILCs, we tested whether PD-1 blockade can enhance T-BET^+^ILC subset in primary cSCCs. Our data demonstrate that culturing TILs in the presence of PD-1 blocking antibody can significantly increase T-BET^+^ ILC proliferation (Fig. 8 *H* and *I*). Our data identify a functional role for PD-1 on T-BET^+^ ILCs in cSCC. Taken together, these data suggest that PD-1^+^ type 1 ILCs are likely to be involved in tumor immunity.

## Discussion

The efficacy of anti-PD-1-based therapies for multiple cancers, such as melanoma, bladder, lung cancers, and Hodgkin lymphoma, has been overwhelmingly demonstrated by clinical trials and experimental murine models. The central premise of anti-PD-1 based therapies suggests that these interventions rescue dampened CD8^+^ T cell function within the TME resulting in more robust antitumor responses. Recent literature demonstrates that PD-1 can also enhance innate immunity within TME, but the initial innate immune population that is activated by PD-1 in exerting antitumor effect is unclear with some studies suggesting NK cells while others indicate myeloid cells (48, 49). The ability of PD-1 to regulate innate immune cell function, including ILCs implicate a potential use for checkpoint inhibitors in harnessing tissue-mediated immunity within the TME that are not enriched in inflammatory infiltrates. Supporting this hypothesis are recent reports demonstrating that blocking antibodies to PD-1 can drive IL-33 primed ILC-2 function within the pancreatic and metastatic TME (25, 36).

The role of ILCs in promoting antitumor responses has been controversial since all helper like ILCs have been implicated in both pro- and anti-tumorigenic functions within the literature. However, whether these populations can be harnessed by using checkpoint inhibitors is not known. We have found roles for PD-1 on ILCs within TME, which include 1) identifying a subset of Tbet^+^NK1.1^−^ ILC population within TME that expressed PD-1, 2) determining that PD-1 selectively increases the frequency and function of Tbet^+^NK1.1^−^ ILCs within a number of solid cancers, and 3) demonstrating PD-1 regulates the proliferation of both murine and human T-bet^+^ ILCs by harnessing metabolic regulation. Our study demonstrates that Tbet^+^NK1.1^−^ ILCs occupy the TME and are regulated by PD-1. Our study demonstrates a type 1 subset and identifies a regulatory role for PD-1 within tumor resident Tbet^+^NK1.1^−^ ILCs.

In B16, the role of ILC-2s in driving antitumor responses has been extensively reported (22, 24, 50, 51). However, these studies consistently studied ILC-2 function within the context of exogenous IL-33 with no data on whether PD-1 can regulate ILCs in the absence of exogenous cytokines. In our experimental systems, using a single-cell approach, experimental murine models and ex vivo human immune cell cultures, we found that PD-1-deficient mice had significantly increased number of Tbet^+^NK1.1^−^ ILCs in various TMEs. In addition, this population was found in both WT and PD-1 KO mice in similar frequency within steady state. These data suggest that although healthy tissues are enriched in different ILC subsets, when perturbed with tumor, anti-PD-1 treatment can expand Tbet^+^NK1.1^−^ ILC in different tumor types and enhance production of IFNγ by this subset. These data drive the possibility of uniform treatment strategies for multiple solid cancers that are not enriched for inflammatory infiltrates whereby PD-1 blockade could be used to boost tissue resident Tbet^+^NK1.1^−^ ILC which possess an immune type 1 phenotype.

Our data also suggest that there is a functional consequence to increased NK1.1^−^Tbet^+^ ILC numbers within the TME. In line with previous reports, depleting NK cells in *Rag^−/−^* mice did not inhibit tumor progression (24) but combining NK depletion with PD-1 blocking antibody significantly increased survival of *Rag^−/−^* mice with B16 tumors and showed significant reduction in tumor volume. This antitumor response was primarily driven by ILCs with a type 1 phenotype. We did not find any differences within other innate immune cell compartment including macrophages as previously reported (49), and on depleting ILCs in *Rag^−/−^* mice, any protection against tumor was lost. The difference in our study versus Gordon et al., could be due to the timing of treatment of the tumor. In our experimental setup, we first allowed the tumors to be established and then deleted ILCs using antibody treatment and therefore were able to isolate the treatment effect of PD-1. We conclude that in the absence of adaptive immunity, myeloid and NK cells, Tbet^+^NK1.1^−^ ILCs play a predominant role in antitumor responses in the presence of anti-PD-1 blocking antibodies. We also propose that Tbet^+^NK1.1^−^ ILCs may drive robust adaptive immune responses in the presence of anti-PD-1 blocking antibodies.

In the study by Hsu et al. on the role of PD-1 in modifying NK cell function in a metastatic lung melanoma model, a significant decrease in tumor size was noted with PD-1 blockade, which the authors largely attributed to NK cells (48). However PD-1 expression in NK cells remains under investigation, as some groups suggest that the expression of PD-1 on NK cells may be coopted from other immune and tumor cells within the TME (52–54). In our experimental system, PD-1 blockade led to enhanced numbers of NK cells within the TME together with a measurable but not-significant reduction in tumor volume compared with mice depleted of NK1.1 cells treated with anti-PD-1. Of note, in the absence of NK cells, antitumor function is largely driven by ILCs in the presence of PD-1 blockade with no significant changes noted within the myeloid/DC compartment.

In contrast to our previous observations in host–pathogen responses (32), significantly reduced ILC-2 numbers within the tumor were noted in melanoma due to increased lactate production (24). This immune suppression mediated by melanoma can be overcome by administrating IL-33, (24) and furthermore, PD-1 blockade in combination with exogenous IL-33 can enhance ILC-2-mediated chemokine production. Increase in chemokine production recruited DCs which in turn enhanced CD8^+^ tumor immunity in pancreatic cancer (25). Herein lies the importance of our current study which specifically focuses on how PD-1 regulates Tbet^+^NK1.1^−^ ILCs in the absence of exogenous IL-33, within the TME. Our data uncouple the effect of exogenous IL-33 and PD-1 within the TME and demonstrate that PD-1 selectively regulates T-bet^+^ ILCs.

The molecular mechanism by which PD-1 can regulate T-bet^+^ILC populations within the TME is not known. We found a unique expression pattern for PD-1 on ILCs whereby it was restricted to the TME ILCs and not surrounding normal skin. Furthermore, within the TME, PD-1 expression is selectively increased on T-bet^+^ ILCs, suggesting a role for tumor-derived products activating the immune compartment in TME resulting in PD-1 expression (55). In line with work by Wagner et al. and Kumagai et al., we found that lactate, which is produced by tumor cells, is capable of inducing PD-1 on T-bet^+^ ILCs. Blocking PD-1 specifically enhanced proliferation of T-bet^+^ILC in both in vivo murine and in vitro human tumor models. These data demonstrate a new tumor-derived immune evasion program driven by lactate and PD-1 by which T-bet^+^ILC-driven antitumor responses are inhibited. We propose that consistent with tumor-derived Tregs, tumor-derived ILCs metabolize lactic acid into phosphoenolpyruvate, which in turn increases calcium ion concentration within the cytoplasm, thereby promoting NFAT translocation into the nucleus. In turn, nuclear NFAT may enhance the expression of PD-1 (55).

We next sought to investigate whether T-bet^+^ ILC proliferation was controlled by the metabolic regulation of ILCs by PD-1. Indeed, in line with ILC-2 biology (56), we found that within the TME, Tbet^+^NK1.1^−^ ILCs predominantly use fatty acid oxidation for their metabolic demands. However, when PD-1 was absent, fatty acid metabolism was significantly down-regulated with a concomitant increase in glycolysis similar to ILC-2s (40). Our work confirms recent observations with respect to PD-1 biology in T cells (38) and in ILC-2s. It was originally described by Wilhelm et al. that ILC-2s were predominantly dependent on fatty acid oxidation for their primary metabolic requirements and inhibition of fatty acid oxidation affects ILC-2 effector functions. This notion was then tested within ILC-2 biology in the absence of PD-1 in airway hyperreactivity. In airway hyperreactivity, Helou et al. demonstrated that in the absence of PD-1, ILC-2s activated by exogenous IL-33 exhibit enhanced aerobic glycolysis and up-regulated amino acid degradation including glutamine and methionine catabolism thereby enabling ILC proliferation. Similar to tumor data on ILC-2s (25, 36), the effect of PD-1 on ILC-2 metabolic switch is apparent only with exogenous IL-33 priming of animals and is not apparent in the absence of IL-33 as an alarmin. It also raises the possibility that IL-25 and TSLP (other ILC-2 alarmins) may not induce the same outcome in ILC-2s that are deficient in PD-1 and this metabolic mechanism remains to be elucidated under these different alarmins. The caveat with studies that include exogenous IL-33 as an alarmin is as follows: These studies may be restricted to a biological output primarily driven by IL-33/PD-1 axis and may not fully capture the function of PD-1 signaling that occurs in activated ILCs within the tumor or tissue microenvironment. Herein lies the importance of our work which demonstrates a specific functional role for PD-1 in TME in response to tumor-derived alarmins. In our study, we show that PD-1 intrinsically controls the metabolic switch in T-bet^+^ ILCs within the TME, and this control results in a functional output whereby an increase in mTOR pathway is noted. Hence, our work identifies an intrinsic mechanistic function for PD-1 in tumor-derived ILCs without exogenous alarmins introduced within the system. Taken together, our data suggest that PD-1 is induced on T-bet^+^ ILCs by tumor-derived lactate, which then selectively inhibits the proliferation of the T-bet^+^ ILC within the TME by modulating metabolic pathways.

Several immunotherapeutic strategies exist for the treatment of melanoma, but there is a single approved systemic immunotherapeutic regimen for advanced cSCC (57). cSCC is characterized by high mutational burden, is frequently seen in patients on immune suppressive drugs, and has been reported to have a dysfunctional peritumoral immune response (58–60) which suggests that it may respond well to immunotherapy. Recently, the use of PD-1 blocking antibodies has been tested in cSCC patients (57, 61, 62) and has shown significant efficacy in half of the patients treated but the underlying immunobiology is not yet understood. Our data demonstrating that PD-1 has a functional role in ILCs within human cSCC tumors suggest that ILCs may play a role in antitumor immunity in cSCC patients with PD-1 controlling this population.

The presence of ILC-1s in tumor has been previously reported in multiple studies and the expression pattern of coinhibitory receptors has been reported. Given the heterogeneity of this population, and due to a significant number of transcriptomic studies on this population it is difficult to discern whether ILC-1 is pro- or anti-tumorigenic. Several caveats remain in this field, the two most significant being the following: a) Transcriptomics definitions are rarely confirmed by protein expression (for example: T-bet protein which can define a type 1 function in both innate and adaptive immune cells), b) most ILC-1 protein definitions are restricted to surface receptor proteins such as CD49, c-kit, CRTh2, CD5, and CD127 in mice and humans, and c) although coinhibitory receptor expression is analyzed, none of the reports test whether the coinhibitory receptors are coexpressed with Tbet and second whether these coinhibitory receptors render functional changes to the ILC population. We have resolved these issues in our work by identifying ILCs based on existing literature but with further analysis of Tbet protein coexpression with PD-1. Here, we show that similar to our murine study, a type 1 ILC population exists within cSCC and can be characterized as CRTH2^−^CD117^−^CD127^+^Tbet^+^ and is controlled by PD-1. This population is characteristically different to the one described in cSCC by Luci et al. (46) based on CD56 and Tbet expression and is responsive to anti-PD-1 therapy. Taken together, we have defined a PD-1-dependent ILC population that participates in antitumor immunity.

In summary, we demonstrate that PD-1 regulates the frequency and function of Tbet^+^NK1.1^−^ helper ILCs in a tumor-dependent manner. It remains to be deciphered to what extent Tbet^+^NK1.1^−^ ILCs can be harnessed for boosting antitumor responses by using PD-1-based immunotherapies. However, the data presented in this study clearly highlights the critical need to understand the regulatory function of PD-1 on ILC subsets to fully harness the potential of PD-1 in modulating immune responses through ILCs.

## Materials and Methods (See *SI Appendix* for detailed methods)

### Animals.

Wild-type C57BL/6 (WT), B6.*Pdcd1^−/−^* (*Pdcd1*^−/−^)*, B6.Pdcd1^−/−^TbetZsGreen, B6.TBetZsGreen,* and *B6.Rag2^−/−^* littermates were bred and maintained in a pathogen-free facility at Newcastle University under a home office-approved project license. All experimental animals were 8 to 12 wk of age. All murine experimental procedures were performed at Newcastle University incorporating the NC3R guidelines for animal research, and the data were presented according to the ARRIVE guidelines.

### Tumor Models (See *SI Appendix*).

Tumor models used in the manuscript are expanded within the extended methods section of the *SI Appendix*.

### ILC Isolation from Tumor.

#### TIL isolation.

ILCs from the tumor tissue were isolated as previously described (63). The detailed methodology is expanded in *SI Appendix*.

### Flow Cytometry.

Single-cell suspensions were generated from indicated organs and stained with Live/Dead fixable dead cell stain kit as per the manufacturer’s instructions (Invitrogen). For murine ILC analysis, cells were incubated with biotin-labeled lineage cocktail (CD3^+^, CD5^+^, CD8^+^, CD11b^+^, CD11c^+^, CD19^+^, CD49b^+^, Ter119^+^, F4/80^+^, B220^+^, NK1.1^+^, and Gr1^+^) followed by streptavidin. Cells were then stained with a combination of markers including CD45, CD90.2, CD127, CD25, KLRG1, Nkp46, PD-1, PD-L1, PD-L2, and ST2.

Human PBMCs and TILs were washed with PBS prior to staining. Then, 1 × 10^6^ cells were stained with Live/Dead fixable dead stain kit as per the manufacturer’s instructions (Invitrogen). Cells were then incubated with cell surface antibodies: Lineage cocktail BV510 or FITC (CD3^+^, CD5^+^, CD11b^+^, CD11c^+^, CD14^+^, CD16^+^, CD19^+^, CD20^+^, CD56^+^, and TCRa/b^+^), CD45, CD127, CD161, CRTH2, c-Kit, Nkp44, and PD-1. Cells were then fixed and permeabilized (Fixation/Permeabilization kit; BD Bioscience) and stained for intracellular transcription factors as follows: Tbet and RORγt. Detailed methodology is expanded in *SI Appendix*.

### Statistical Analysis.

Statistical analysis was performed with GraphPad Prism. The specific tests are included in the figure legends.

## Supplementary Material

Appendix 01 (PDF)

Dataset S01 (XLSX)

Dataset S02 (XLSX)

Dataset S03 (XLSX)

## Data Availability

All study data are included in the article and *SI Appendix*. Data has been deposited in the Gene Expression Omnibus (https://www.ncbi.nlm.nih.gov/geo/query/acc.cgi?acc=GSE229288) (64).

## References

[1] H. Spits , Innate lymphoid cells–a proposal for uniform nomenclature. Nat. Rev. Immunol. **13**, 145–149 (2013). 23348417 10.1038/nri3365

[2] J. H. Bernink , Human type 1 innate lymphoid cells accumulate in inflamed mucosal tissues. Nat. Immunol. **14**, 221–229 (2013). 23334791 10.1038/ni.2534

[3] A. Fuchs , Intraepithelial type 1 innate lymphoid cells are a unique subset of IL-12- and IL-15-responsive IFN-gamma-producing cells. Immunity **38**, 769–781 (2013). 23453631 10.1016/j.immuni.2013.02.010 PMC3634355

[4] C. S. N. Klose , Differentiation of type 1 ILCs from a common progenitor to all helper-like innate lymphoid cell lineages. Cell **157**, 340–356 (2014). 24725403 10.1016/j.cell.2014.03.030

[5] C. Daussy , T-bet and Eomes instruct the development of two distinct natural killer cell lineages in the liver and in the bone marrow. J. Exp. Med. **211**, 563–577 (2014). 24516120 10.1084/jem.20131560 PMC3949572

[6] H. Peng , Liver-resident NK cells confer adaptive immunity in skin-contact inflammation. J. Clin. Invest. **123**, 1444–1456 (2013). 23524967 10.1172/JCI66381 PMC3613925

[7] J. M. Mjosberg , Human IL-25- and IL-33-responsive type 2 innate lymphoid cells are defined by expression of CRTH2 and CD161. Nat. Immunol. **12**, 1055–1062 (2011). 21909091 10.1038/ni.2104

[8] L. A. Monticelli , Innate lymphoid cells promote lung-tissue homeostasis after infection with influenza virus. Nat. Immunol. **12**, 1045–1054 (2011). 21946417 10.1031/ni.2131 PMC3320042

[9] K. Moro , Innate production of T(H)2 cytokines by adipose tissue-associated c-Kit(+)Sca-1(+) lymphoid cells. Nature **463**, 540–544 (2010). 20023630 10.1038/nature08636

[10] D. R. Neill , Nuocytes represent a new innate effector leukocyte that mediates type-2 immunity. Nature **464**, 1367–1370 (2010). 20200518 10.1038/nature08900 PMC2862165

[11] M. Cella , A human natural killer cell subset provides an innate source of IL-22 for mucosal immunity. Nature **457**, 722–725 (2009). 18978771 10.1038/nature07537 PMC3772687

[12] G. F. Sonnenberg, L. A. Monticelli, M. M. Elloso, L. A. Fouser, D. Artis, CD4(+) lymphoid tissue-inducer cells promote innate immunity in the gut. Immunity **34**, 122–134 (2011). 21194981 10.1016/j.immuni.2010.12.009 PMC3035987

[13] S. Sawa , Lineage relationship analysis of RORgammat+ innate lymphoid cells. Science **330**, 665–669 (2010). 20929731 10.1126/science.1194597

[14] N. Satoh-Takayama , Microbial flora drives interleukin 22 production in intestinal NKp46+ cells that provide innate mucosal immune defense. Immunity **29**, 958–970 (2008). 19084435 10.1016/j.immuni.2008.11.001

[15] S. Buonocore , Innate lymphoid cells drive interleukin-23-dependent innate intestinal pathology. Nature **464**, 1371–1375 (2010). 20393462 10.1038/nature08949 PMC3796764

[16] S. L. Sanos , RORgammat and commensal microflora are required for the differentiation of mucosal interleukin 22-producing NKp46+ cells. Nat. Immunol. **10**, 83–91 (2009). 19029903 10.1038/ni.1684 PMC4217274

[17] T. Cupedo , Human fetal lymphoid tissue-inducer cells are interleukin 17-producing precursors to RORC+ CD127+ natural killer-like cells. Nat. Immunol. **10**, 66–74 (2009). 19029905 10.1038/ni.1668

[18] N. K. Crellin , Regulation of cytokine secretion in human CD127(+) LTi-like innate lymphoid cells by Toll-like receptor 2. Immunity **33**, 752–764 (2010). 21055975 10.1016/j.immuni.2010.10.012

[19] Y. Gao , Tumor immunoevasion by the conversion of effector NK cells into type 1 innate lymphoid cells. Nat. Immunol. **18**, 1004–1015 (2017). 28759001 10.1038/ni.3800

[20] I. P. Jovanovic , Interleukin-33/ST2 axis promotes breast cancer growth and metastases by facilitating intratumoral accumulation of immunosuppressive and innate lymphoid cells. Int. J. Cancer **134**, 1669–1682 (2014). 24105680 10.1002/ijc.28481

[21] J. Li , Biliary repair and carcinogenesis are mediated by IL-33-dependent cholangiocyte proliferation. J. Clin. Invest. **124**, 3241–3251 (2014). 24892809 10.1172/JCI73742 PMC4071370

[22] M. Ikutani , Identification of innate IL-5-producing cells and their role in lung eosinophil regulation and antitumor immunity. J. Immunol. **188**, 703–713 (2012). 22174445 10.4049/jimmunol.1101270

[23] I. Saranchova , Type 2 innate lymphocytes actuate immunity against tumours and limit cancer metastasis. Sci. Rep. **8**, 2924 (2018). 29440650 10.1038/s41598-018-20608-6 PMC5811448

[24] M. Wagner , Tumor-derived lactic acid contributes to the paucity of intratumoral ILC-2s. Cell Rep. **30**, 2743–2757.e5 (2020). 32101749 10.1016/j.celrep.2020.01.103

[25] J. A. Moral , ILC-2s amplify PD-1 blockade by activating tissue-specific cancer immunity. Nature **579**, 130–135 (2020). 32076273 10.1038/s41586-020-2015-4 PMC7060130

[26] S. Kirchberger , Innate lymphoid cells sustain colon cancer through production of interleukin-22 in a mouse model. J. Exp. Med. **210**, 917–931 (2013). 23589566 10.1084/jem.20122308 PMC3646494

[27] I. H. Chan , Interleukin-23 is sufficient to induce rapid de novo gut tumorigenesis, independent of carcinogens, through activation of innate lymphoid cells. Mucosal. Immunol. **7**, 842–856 (2014). 24280935 10.1038/mi.2013.101

[28] M. Eisenring, J. vom Berg, G. Kristiansen, E. Saller, B. Becher, IL-12 initiates tumor rejection via lymphoid tissue-inducer cells bearing the natural cytotoxicity receptor NKp46. Nat. Immunol. **11**, 1030–1038 (2010). 20935648 10.1038/ni.1947

[29] K. Nussbaum , Tissue microenvironment dictates the fate and tumor-suppressive function of type 3 ILCs. J. Exp. Med. **214**, 2331–2347 (2017). 28698286 10.1084/jem.20162031 PMC5551572

[30] M. Salimi , Activated innate lymphoid cell populations accumulate in human tumour tissues. BMC Cancer **18**, 341 (2018). 29587679 10.1186/s12885-018-4262-4 PMC5870240

[31] H. Maazi , ICOS:ICOS-ligand interaction is required for type 2 innate lymphoid cell function, homeostasis, and induction of airway hyperreactivity. Immunity **42**, 538–551 (2015). 25769613 10.1016/j.immuni.2015.02.007 PMC4366271

[32] S. Taylor , PD-1 regulates KLRG1(+) group 2 innate lymphoid cells. J. Exp. Med. **214**, 1663–1678 (2017). 28490441 10.1084/jem.20161653 PMC5461001

[33] C. Schwartz , ILC-2s regulate adaptive Th2 cell functions via PD-L1 checkpoint control. J. Exp. Med. **214**, 2507–2521 (2017). 28747424 10.1084/jem.20170051 PMC5584124

[34] C. S. N. Klose, D. Artis, Innate lymphoid cells control signaling circuits to regulate tissue-specific immunity. Cell Res. **30**, 475–491 (2020). 32376911 10.1038/s41422-020-0323-8 PMC7264134

[35] N. Jacquelot , Immune checkpoints and innate lymphoid cells-new avenues for cancer immunotherapy. Cancers (Basel) **13**, 5967 (2021). 34885076 10.3390/cancers13235967 PMC8657134

[36] N. Jacquelot , Blockade of the co-inhibitory molecule PD-1 unleashes ILC-2-dependent antitumor immunity in melanoma. Nat. Immunol. **22**, 851–864 (2021). 34099918 10.1038/s41590-021-00943-z PMC7611091

[37] B. Heinrich , The tumour microenvironment shapes innate lymphoid cells in patients with hepatocellular carcinoma. Gut **71**, 1161–1175 (2022). 34340996 10.1136/gutjnl-2021-325288 PMC8807808

[38] N. Patsoukis , PD-1 alters T-cell metabolic reprogramming by inhibiting glycolysis and promoting lipolysis and fatty acid oxidation. Nat. Commun. **6**, 6692 (2015). 25809635 10.1038/ncomms7692 PMC4389235

[39] L. Strauss , Targeted deletion of PD-1 in myeloid cells induces antitumor immunity. Sci. Immunol. **5**, eaay1863 (2020). 31901074 10.1126/sciimmunol.aay1863 PMC7183328

[40] D. G. Helou , PD-1 pathway regulates ILC-2 metabolism and PD-1 agonist treatment ameliorates airway hyperreactivity. Nat. Commun. **11**, 3998 (2020). 32778730 10.1038/s41467-020-17813-1 PMC7417739

[41] L. Mazzurana , Tissue-specific transcriptional imprinting and heterogeneity in human innate lymphoid cells revealed by full-length single-cell RNA-sequencing. Cell Res. **31**, 554–568 (2021). 33420427 10.1038/s41422-020-00445-x PMC8089104

[42] J. Qi , Single-cell transcriptomic landscape reveals tumor specific innate lymphoid cells associated with colorectal cancer progression. Cell Rep. Med. **2**, 100353 (2021). 34467243 10.1016/j.xcrm.2021.100353 PMC8385246

[43] F. Vely , Evidence of innate lymphoid cell redundancy in humans. Nat. Immunol. **17**, 1291–1299 (2016). 27618553 10.1038/ni.3553 PMC5074366

[44] A. I. Lim , Systemic human ILC precursors provide a substrate for tissue ILC differentiation. Cell **168**, 1086–1100.e10 (2017). 28283063 10.1016/j.cell.2017.02.021

[45] M. Nagasawa , KLRG1 and NKp46 discriminate subpopulations of human CD117(+)CRTH2(-) ILCs biased toward ILC-2 or ILC-3. J. Exp. Med. **216**, 1762–1776 (2019). 31201208 10.1084/jem.20190490 PMC6683990

[46] C. Luci , Cutaneous squamous cell carcinoma development is associated with a temporal infiltration of ILC-1 and NK cells with immune dysfunctions. J. Invest. Dermatol. **141**, 2369–2379 (2021). 33831432 10.1016/j.jid.2021.03.018

[47] A. L. Ji , Multimodal analysis of composition and spatial architecture in human squamous cell carcinoma. Cell **182**, 497–514.e22 (2020). 32579974 10.1016/j.cell.2020.05.039 PMC7391009

[48] J. Hsu , Contribution of NK cells to immunotherapy mediated by PD-1/PD-L1 blockade. J. Clin. Invest. **128**, 4654–4668 (2018). 30198904 10.1172/JCI99317 PMC6159991

[49] S. R. Gordon , PD-1 expression by tumour-associated macrophages inhibits phagocytosis and tumour immunity. Nature **545**, 495–499 (2017). 28514441 10.1038/nature22396 PMC5931375

[50] J. Kim , Intratumorally establishing type 2 innate lymphoid cells blocks tumor growth. J. Immunol. **196**, 2410–2423 (2016). 26829987 10.4049/jimmunol.1501730

[51] A. Long , Type 2 innate lymphoid cells impede IL-33-mediated tumor suppression. J. Immunol. **201**, 3456–3464 (2018). 30373846 10.4049/jimmunol.1800173 PMC6264920

[52] M. S. Hasim , When killers become thieves: Trogocytosed PD-1 inhibits NK cells in cancer. Sci. Adv. **8**, eabj3286 (2022). 35417234 10.1126/sciadv.abj3286 PMC9007500

[53] M. M. Cho, A. E. Quamine, M. R. Olsen, C. M. Capitini, Programmed cell death protein 1 on natural killer cells: Fact or fiction? J. Clin. Invest. **130**, 2816–2819 (2020). 32391808 10.1172/JCI137051 PMC7260016

[54] S. J. Judge , Minimal PD-1 expression in mouse and human NK cells under diverse conditions. J. Clin. Invest. **130**, 3051–3068 (2020). 32134744 10.1172/JCI133353 PMC7260004

[55] S. Kumagai , Lactic acid promotes PD-1 expression in regulatory T cells in highly glycolytic tumor microenvironments. Cancer Cell **40**, 201–218.e9 (2022). 35090594 10.1016/j.ccell.2022.01.001

[56] C. Wilhelm , Critical role of fatty acid metabolism in ILC-2-mediated barrier protection during malnutrition and helminth infection. J. Exp. Med. **213**, 1409–1418 (2016). 27432938 10.1084/jem.20151448 PMC4986525

[57] M. R. Migden , PD-1 blockade with cemiplimab in advanced cutaneous squamous-cell carcinoma. N. Engl. J. Med. **379**, 341–351 (2018). 29863979 10.1056/NEJMoa1805131

[58] C. Lai , OX40+ regulatory T cells in cutaneous squamous cell carcinoma suppress effector T-cell responses and associate with metastatic potential. Clin. Cancer Res. **22**, 4236–4248 (2016). 27034329 10.1158/1078-0432.CCR-15-2614 PMC4987192

[59] A. Shapanis , Identification of proteins associated with development of metastasis from cutaneous squamous cell carcinomas (cSCCs) via proteomic analysis of primary cSCCs. Br. J. Dermatol. **184**, 709–721 (2021). 32794257 10.1111/bjd.19485

[60] C. Lai , CD8+CD103+ tissue-resident memory T cells convey reduced protective immunity in cutaneous squamous cell carcinoma. J. Immunother. Cancer **9**, e001807 (2021). 33479027 10.1136/jitc-2020-001807 PMC7825273

[61] M. L. M. van Baar, A. D. Guminski, P. M. Ferguson, L. K. Martin, Pembrolizumab for cutaneous squamous cell carcinoma: Report of a case of inoperable squamous cell carcinoma with complete response to pembrolizumab complicated by granulomatous inflammation. JAAD Case Rep. **5**, 491–494 (2019). 31193588 10.1016/j.jdcr.2019.04.006 PMC6536769

[62] G. S. Falchook , Responses of metastatic basal cell and cutaneous squamous cell carcinomas to anti-PD1 monoclonal antibody REGN2810. J. Immunother. Cancer **4**, 70 (2016). 27879972 10.1186/s40425-016-0176-3 PMC5109769

[63] G. Mallett, W. Patterson, M. Payne, S. Amarnath, Isolation and characterization of innate lymphoid cells within the murine tumor microenvironment. Methods Mol. Biol. **2121**, 153–164 (2020). 32147794 10.1007/978-1-0716-0338-3_14

[64] J. X. Lim , Data for “Programmed cell death-1 receptor mediated regulation of Tbet+NK1.1- Innate Lymphoid Cells Within the Tumor.” Gene Expression Omnibus. https://www.ncbi.nlm.nih.gov/geo/query/acc.cgi?acc=GSE229288. Deposited 10 April 2023. 10.1073/pnas.2216587120 PMC10161089 37098069

