# Supplementary material for: Programmed cell death-1 receptor-mediated regulation of Tbet+NK1.1− innate lymphoid cells within the tumor microenvironment
Source: Proc Natl Acad Sci U S A. 2023 Apr 25;120(18):e2216587120. doi: 10.1073/pnas.2216587120 (PMC10161089; doi:10.1073/pnas.2216587120)
Supplement: Supplementary file 1 — Appendix 01 (PDF) [file pnas.2216587120.sapp.pdf]

Programmed cell death-1 receptor mediated regulation of Tbet^{+} NK1.1^{-} Innate Lymphoid Cells within the Tumor Microenvironment

Jing Xuan Lim[1,2*], Chester Y. Lai[3,4*], Grace E. Mallett[1,2], David McDonald[1], Gillian Hulme[1], Stephanie Laba[1], Andrew Shapanis[3], Megan Payne[1], Warren Patterson[1], Michael Alexander[2,] Jonathan Coxhead[1], Andrew Filby[1], Ruth Plummer[1,5], Penny E. Lovat[1,5], Giuseppe Sciume[6], Eugene Healy[3,4] and Shoba Amarnath[1,2].

[1]Newcastle University Biosciences Institute, [2]Newcastle University Centre for Cancer, Medical School, Newcastle University, NE2 4HH. [3]Dermatopharmacology, Clinical and Experimental Sciences, Faculty of Medicine, University of Southampton, Southampton, SO16 6YD, UK. [4]Dermatology, University Hospital Southampton NHS Foundation Trust, Southampton, SO16 6YD, UK, [5]Newcastle University Translational and Clinical Research Institute, Medical School, Newcastle University, NE2 4HH. [6]Department of Molecular Medicine, Sapienza University of Rome Laboratory affiliation to Institute Pasteur Italia-Fondazione Cenci Bolognetti, Rome, Italy.

*Equal Contribution

Corresponding Author

Shoba Amarnath, PhD

Immune Regulation Laboratory

Newcastle University Centre for Cancer

The Medical School

Newcastle University,

NE24HH,

UK.

Email: shoba.amarnath@newcastle.ac.uk

Extended Description of Methods

Materials and Methods

Cell lines

B16F10 melanoma and MC38 adenocarcinoma colon cancer were kindly provided by Dr Arunakumar Gangaplara, NCI, NIH. Tumor cells were cultured in Dulbecco's Modified Eagle Medium (DMEM) supplemented with 10% FBS (Labtech), sodium pyruvate (1mM; Sigma) and penicillin-streptomycin (100 units/ml penicillin; 100µg/ml streptomycin; Gibco^{TM}) and maintained at 37°C with 5% CO_2. C8161 human melanoma cell line and MET1 human squamous cell carcinoma (SCC) cell line (Ximbio) were kindly donated by Professor Penny Lovat, Newcastle University. C8161 cells were cultured with DMEM media supplemented with 10% FBS (Labtech), sodium pyruvate (1mM; Sigma) and penicillin-streptomycin (100 units/ml penicillin; 100µg/ml streptomycin; Gibco^{TM}). MET1 cells were cultured with DMEM/F12 media (4:1) supplemented with 10% FBS (Labtech), penicillin streptomycin (100units/ml penicillin; 100µg/ml streptomycin), hydrocortisone (0.4µg/ml; Sigma), cholera toxin (8.5ng/ml; Sigma), tri-iodo-L-threonine (20pM; Sigma), Adenine (180µM; Sigma), Insulin (5µg/ml; Sigma), epithelial growth factor (EGF; 2pg/ml; Sigma) and transferrin (5µg/ml; Sigma).

Tumor Models

Subcutaneous tumor models of B16F10 and MC38

WT or B6.*Pdcd1*$^{-/-}$, B6.*Pdcd1*$^{-/-}$TbetZsGreen, B6.TbetZsGreen, B6.*Rag2*$^{-/-}$ mice were inoculated with 2 x 10^5 B16F10 melanoma cells or MC38 cells via subcutaneous injection into the flank as previously reported [1]. Tumor volume was recorded daily from day 5 post inoculation. Tumor volume was calculated by the following equation: Tumor volume = π/6 x 0.5 x length x width^2 as described previously [2]. In certain experiments, mice were treated with anti-PD-1 (200µg/mouse; clone: RPMI-40); anti-NK1.1 (200µg/mouse; clone:PK136) or isotype control (IgG2a, 200µg/mouse, clone: 2A3; IgG1a, 200µg/mouse; C1.18.24 respectively) via intraperitoneal injection at day 7, 9 and 11 unless otherwise indicated. Mice were euthanized at day 12, unless otherwise stated for tumor infiltrating lymphocyte (TIL) immunobiology assays. In some experiments, animals were injected with BODIPY (200µg/mouse) or vehicle before harvest. None of these experiments included exogenous IL-33 administration.

Metastatic melanoma

WT or *B6.TbetZsGreen or B6.Pdcd1$^{-/-}$* mice were inoculated with either 0.5 or 2 x 10^5 B16F10 melanoma cells in 200µl of PBS via intravenous tail-vein injection. Mice were monitored for clinical signs of weight loss and euthanized at >25% weight loss. In certain experiments mice were treated with anti-PD-1 (200µg/mouse; clone: RPMI-40) or isotype control (IgG2a, 200µg/mouse; clone: 2A3) at day 7, 9, 11. Tissue was harvested at day 12 and analyzed for the presence of ILCs unless stated otherwise. None of these experiments included exogenous IL-33 administration.

AOM-DSS induced colorectal cancer

The AOM-DSS model was set up as previously reported [3]. Briefly, mice were treated with one dose of Azoxymethane (AOM; 12mg/kg) at day 1 and then 3% dextran sodium sulphate (DSS) was added as drinking water from day 3 for a week. Mice were allowed to recover for 3 weeks and then a 2nd cycle of DSS was started. After the 3rd cycle, animals were euthanized and immunobiology studied. None of these experiments included exogenous IL-33 administration.

ILC isolation from tumor and normal tissue

TIL isolation

ILCs from the tumor tissue were isolated as previously described [4]. Briefly, tumor tissue was incubated at 37°C for 30minutes in FBS free DMEM media containing liberase™ TL (0.25mg/ml; Roche) and DNAse I (0.5mg/ml; Roche). Single-cell suspensions were prepared by mechanically disrupting tissue through a 100µm nylon cell strainer into FBS. Lymphocytes were isolated using lymphocyte separation media (LSM; Promocell) and washed twice with complete media (DMEM supplemented with 10% FBS, glutamine (2mM), non-essential amino acids (0.1mM), sodium pyruvate (1mM), 2-mercaptoethanol (50µM) and penicillin and streptomycin (100 units/ml penicillin; 100µg/ml streptomycin; Gibco™) in order to remove traces of LSM. Cells were then analyzed by flow cytometry or stimulated to induce cytokine production. Ethical approval for experiments conducted on human tissue was provided by the South Central Hampshire B NRES Committee (reference number 07/H0504/187). Fresh tissue samples of cSCC and non-lesional skin were obtained from patients during surgery at the Dermatology Department, University Hospital Southampton NHS Foundation Trust. For lymphocyte isolation, samples of tumor and separately, non-lesional skin, were finely

disaggregated with scalpels, incubated at 37°C for 1.5 hours in RPMI media containing collagenase I-A (1mg/ml; Sigma-Aldrich) and DNAse I (10µg/ml; Sigma-Aldrich). The resulting suspension was then passed through a 70µm cell strainer and centrifuged over an Optiprep (Sigma-Aldrich) density gradient. Lymphocytes were then extracted and washed with PBS before use in experiments. In certain experiments, TILs were incubated for 60 mins with 2NBDG (200µg/ml) or PBS and then ILCs analyzed by flow cytometry.

ILC isolation from spleen

Single cell-suspension of splenocytes was generated by mechanically disrupting tissue through a 40µm filter into complete media. Cells were incubated with red blood cell (RBC) lysis buffer (Biolegend) for 3 minutes at room temperature and were then washed in complete media once before analysis by flow cytometry.

ILC isolation from tumor draining lymph nodes

Tumor draining lymph nodes (TDLN) were isolated from the inguinal draining lymph nodes. Single cell-suspension was generated by mechanically disrupting TDLNs through a 40µm filter into complete media. Cells were then washed and analyzed by flow cytometry.

ILC isolation from lungs

Lungs were perfused with PBS *in situ* via the pulmonary artery prior to isolation. Tissue was incubated at 37°C for 15 minutes in FBS free DMEM media containing Liberase™ TL (0.25mg/ml; Roche) and DNAse I (0.5mg/ml; Roche). Single cell suspension was generated by mechanically disrupting tissues through a 100µm filter into FBS followed by a Percoll gradient centrifugation (40% Percoll, GE Healthcare; FBS free media containing 0.5mg/ml DNase, Roche). Cells were then washed with complete media and analyzed by flow cytometry.

ILC isolation from small intestine

Small intestine tissue was harvested into complete media. Fecal matter was removed, and tissue was washed in buffer (PBS containing 5% FBS, Hepes (1mM; Sigma), 50µM 2-mercaptoethanol (50µM; Sigma) and penicillin and streptomycin (100 units/ml penicillin; 100µg/ml streptomycin; Gibco™)). This was followed by a wash with PBS to remove traces of FBS. Tissue was incubated at 37°C for 30 minutes in FBS free DMEM media containing Liberase™ TL (0.25mg/ml; Roche) and DNAse I (0.5mg/ml; Roche). Single cell suspension

was generated by mechanically disrupting tissues through a 100μm filter into FBS. This was followed by percoll gradient centrifugation (40% Percoll (GE Healthcare), containing 0.5mg/ml DNase (Roche)). Cells were washed with complete media and analyzed by flow cytometry.

ILC isolation from skin

Mouse normal dorsal skin was harvested, finely chopped and incubated for 2 hours in FBS free DMEM media containing Liberase^{TM} TL (0.25mg/ml; Roche) and DNAse I (0.5mg/ml; Roche) at 37°C. Single-cell suspensions were prepared by mechanically crushing tissue slurry through a 100μm nylon mesh into FBS. Cells were then subsequently filtered through 70- and 40-μm nylon filters. Cells were then analyzed via flow cytometry.

Antibodies

All the antibodies used to characterize murine and human ILCs were purchased from BioLegend or eBioscience unless otherwise stated. For analysis of murine cell surface markers, the following antibodies were used: Lineage consisted of CD3 (clone: 1452C11), CD5 (clone: 53-7.3), CD8 (clone: 53-6.7), CD11b (clone: M1170), CD11c (clone: N418), CD19 (clone: 1D3/CD19), CD49b (clone: HMα2), Ter119 (clone: TER-119), Gr1 (clone: RB8-9C5), F4/80 (clone:BM8), Nk1.1 (clone:PK136), B220 (clone:RA3-8B2). ILCs were stained for CD45 (clone:30-F11), CD90.2 (clone: 30-H12), CD127 (clone: A7R34), CD25 (clone: PC61), KLRG1 (clone:2F1/KLRG1), Nkp46 (clone:29A1.4), CD49a (clone: HMα1), PD-1 (clone: RPMI-30), PDL-1 (clone:10F.9G2), PDL-2 (clone: Ty25). ST2 (clone:DJ8) was purchased from MD Bioproducts. For ILC analysis in human peripheral blood mononuclear cells (PBMC), the following fluorochrome-conjugated antibodies were used: Lineage consisting of CD3 (clone: OKT3), CD5 (clone: L17F12), CD14 (clone: M5E2), CD16 (HIB19), CD19 (HIB19), CD20 (2H7), CD56 (clone HCD56), CD11b (clone:ICRF44), CD11c (clone: 3.9) and TCRα/β (clone: IP26), CD45 (clone:H130), CD127 (clone:A019D5), CD161 (clone:HP-3G10), c-kit (CD117;clone:104D2), CRTH2 (clone:BM16), PD-1 (EH12.2H7) and Nkp44 (clone:P44-8). For analysis of murine cytokine production, the following fluorochrome-conjugated antibodies were used: IL-5 (clone: TRFK5), IL-10 (clone: JES5-16ES), TNFα (clone: MP6-XT22), IFNγ (clone: XMG1.1), RORγt (clone: B2D), EOMES (clone: Dan11mag), IL-17 (clone: eBio17B7), IL-22 (clone: IL-22JOP), IL-13 (clone: eBio13A) and

Ki67 (clone: So1A15). Human PBMCs were stained with the following intracellular fluorochrome-conjugated antibodies; Tbet (clone: 4B10), IFNγ (clone: 4S.B3), IL-17 (clone: BL168), IL-5 (clone: TRFK5), TNFα (clone: Mab11), RORγt (clone: B2D) and IL-13 (clone: JES10-5A2).

Flow cytometry

Single cell suspensions were generated from indicated organs and stained with Live/Dead fixable dead cell stain kit as per manufacturer's instructions (Invitrogen). For murine ILC analysis, cells were incubated with biotin labeled lineage cocktail ($CD3^+$, $CD5^+$, $CD8^+$, $CD11b^+$, $CD11c^+$, $CD19^+$, $CD49b^+$, $Ter119^+$, $F4/80^+$, $B220^+$, $NK1.1^+$ and $Gr1^+$) followed by streptavidin. Cells were then stained with a combination of markers including CD45, CD90.2, CD127, CD25, KLRG1, NKp46, PD-1, PDL-1, PDL-2 and ST2. For analysis of NK and myeloid immune subsets, TILs were stained with NK1.1, CD49a, CD49b, F4/80, Gr1, CD11b and CD11c. Cells were then fixed and permeabilized for intracellular markers as follows: Tbet, RORγ, Ki67 and EOMES. In order to measure murine intracellular cytokine (IC) production, TILs were stimulated with cytokine stimulation cocktail (Invitrogen; Thermo Fisher Scientific) for 4 hours at 37°C. Cells were then fixed and permeabilized (Fixation/Permeabilization kit; BD Bioscience). ILCs were then stained with: IL-5, IL-13, IL-17, IL-22, IFN-γ and TNF-α.

Human PBMCs and TILs were washed with PBS prior to staining. 1 x 10^6 cells were stained with Live/Dead fixable dead stain kit as per manufactures instructions (Invitrogen). Cells were then incubated with cell surface antibodies: Lineage cocktail BV510 or FITC ($CD3^+$, $CD5^+$, $CD11b^+$, $CD11c^+$, $CD14^+$, $CD16^+$, $CD19^+$, $CD20^+$, $CD56^+$ and $TCR\alpha/\beta^+$), CD45, CD127, CD161, CRTH2, c-Kit, Nkp44 and PD-1. Cells were then fixed and permeabilized (Fixation/Permeabilization kit; BD Bioscience) and stained for intracellular transcription factors as follows: Tbet and RORγt.

ILCs were defined by the following gating strategies: Murine ILCs were defined as Lin^- $Thy1^+$; ILC2s were defined as $CD127^+CD25^+KLRG1^{+/-}ST2^{+/-}$; NCR^+ ILC3s were defined as $ROR\gamma t^+NKp46^+$ and NCR^- ILC3s were defined as $ROR\gamma t^+Nkp46^-$, murine ILC subset regulated by PD-1 was defined as Lin^- $Thy1^+$ $NK1.1^-Tbet^+NKp46^-$. Human ILCs were defined as follows: $Lin^-CD45^+CD161^+CD127^+$. ILC1s were further defined as $CRTH2^-CD117^-$; ILC2s were defined as $CRTH2^+CD117^-$; ILC3s were defined as $CRTH2^-CD117^+$. Cells were analyzed

using BD LSR Fortessa X20 with FACs DIVA software (BD Bioscience) and analysis was performed with FCS Express (De Novo) or FlowJo 10.1 software (Tree Star).

Rhapsody Single Cell Sequencing

TILs were isolated as previously described from tumours and then cells were stained with lineage markers (lineage gate included CD3$^+$, CD4$^+$, CD5$^+$, CD8$^+$, CD11b$^+$, CD11c$^+$, CD19$^+$, CD49b$^+$, Ter119$^+$, F4/80$^+$, B220$^+$ and Gr1$^+$), Thy1, and Abseq antibodies and sample Tags. Abseq antibody-oligos used were as follows: CD25, CD103, CD119, CD37, CD223, CD272, CD273, CD274, CD278, CD279, IL17Rb, IL-23R, IL33R, CD335, CD3 and NK1.1. Cells were incubated for 20 minutes at 4C and then washed three times with Miltenyi buffer. TILs were then stained with DAPI and flow sorted for Lineage$^-$Thy1$^+$ population. Samples were then pooled and loaded on to rhapsody cartridges and then experiment were performed as per manufacturer's instructions. Data analysis was performed using the SeqGeq software.

Transwell assays

Splenocytes were RBC lysed and then plated at a concentration of 0.5 x 10^6 cells per ml. Transwell inserts (0.4 micron; ThermoFisher Scientific) were seeded with B16F10 melanoma cells at a 1:1 ratio with splenocytes (unless otherwise stated) and were incubated for indicated time points at 37°C prior to flow cytometry analysis. For proliferation assays, transwell inserts were removed after 6 hours. For human experiments, PBMCs were acquired from healthy donors and were cultured at a concentration of 0.5 x 10^6 per ml. Transwell inserts were seeded with either C8161 human melanoma cell line or human cSCC cell line at a 1:1 ratio with PBMCs. For human experiments, transwell inserts were removed after 16 hours. Plates were incubated at 37°C for indicated time points and were then analyzed by flow cytometry.

***In-vitro* Proliferation Assays**

For cell trace violet experiments, murine splenocytes isolated from B6.TbetZsGreen mice were stained in PBS with Cell Trace Violet (Invitrogen) as per manufactures instructions. Cells were cultured with IL-2 (40ng/ml), IL-7 (40ng/ml), αPD-1 (20µg/ml; clone: RMP1-14) or Isotype IgG2a (20µg/ml; clone: 2A3) as indicated for 5 days in cell culture media (DMEM supplemented with 10% FBS, glutamine (2mM), non-essential amino acids (0.1mM), sodium pyruvate (1mM), 2-mercaptoethanol (50µM), penicillin and streptomycin (100 U/M)). Cytokines were replenished on day 2 and day 4. Proliferation was measured on day 5 by flow

cytometry. For human proliferation assays, human PBMCs acquired from healthy donors were stained in PBS with Cell Trace Violet (Invitrogen) as per manufacturer's instructions. Cells were cultured with IL-2 (40ng/ml), IL-7 (40ng/ml), αPD-1 (20μg/ml; clone:EH12.2H7) or Isotype IgG1 (20μg/ml; clone: MG1-45) as indicated for 7 days in cell culture media (RPMI supplemented with 10% FBS, glutamine (2mM), non-essential amino acids (0.1mM), sodium pyruvate (1mM), 2-mercaptoethanol (50μM), penicillin and streptomycin ((100 units/ml penicillin; 100μg/ml streptomycin; Gibco^{TM})). Cytokines were replenished on day 2 and day 4. Proliferation was measured on day 7 by flow cytometry.

Lactate Assays

WT or *Pdcd1*$^{-/-}$ splenocytes were incubated with IL-2 (100 ng/ml) plus IL-7 (100 ng/ml) alone or in combination with lactic acid (20 mM) for 24 hrs and then PD-1 expression was measured by flow cytometry. For human studies, $1x10^6$ PBMCs were incubated with IL-2 (100 ng/ml) plus IL-7 (100 ng/ml) alone or in combination with lactic acid (20 mM) for 24 hrs and then PD-1 expression was measured by flow cytometry within the Lineage^{neg}CD45$^+$CD127$^+$CRTh2$^-$ CD117$^-$Tbet$^+$ subset. B16F10 tumor cells were expanded and then supernatant was tested for lactic acid production as per the manufacturer's instructions (Abcam). Briefly, $2x10^6$ cells were seeded in 24 well plates and then supernatant harvested after 4hrs or 24 hrs. The amount of lactic acid was determined using a lactic acid fluorometry kit

Phospho-P70S6Kinase Measurement

Tumors were resected when they reached >600mm^3 and then TILs were isolated. TILs were stimulated with IL-2 (80ng/ml) and IL-7 (40ng/ml) for 15 minutes. TILs were washed once with PBS and then stained for phosphoP70S6Kinase antibody and then analyzed by flow cytometry. In some experiments, TILs were enriched using CD90.2 microbeads as per manufacturer's instructions and then cultured for 3 days with IL-2 plus IL-7 alone or in combination with isotype (20μg/ml) or anti-PD-1 antibody (20μg/ml). At day 3 post cultures, TILs were washed with complete media once and then restimulated with IL-2 and IL-7 for 15mins. Following stimulation, phosphorylation of P70S6Kinase was measured.

Statistical Analysis

Statistical analysis was performed with GraphPad Prism using an unpaired student two-tailed T test for groups of two and a ONE-WAY ANOVA for multiple groups. Results are expressed as mean± standard error of the mean (SEM) and P-values ≤0.05 were considered significant. Survival curve analysis was performed using a Kaplan-Meier survival curve and a log rank test.

Supplementary Figure Legends

Fig.S1

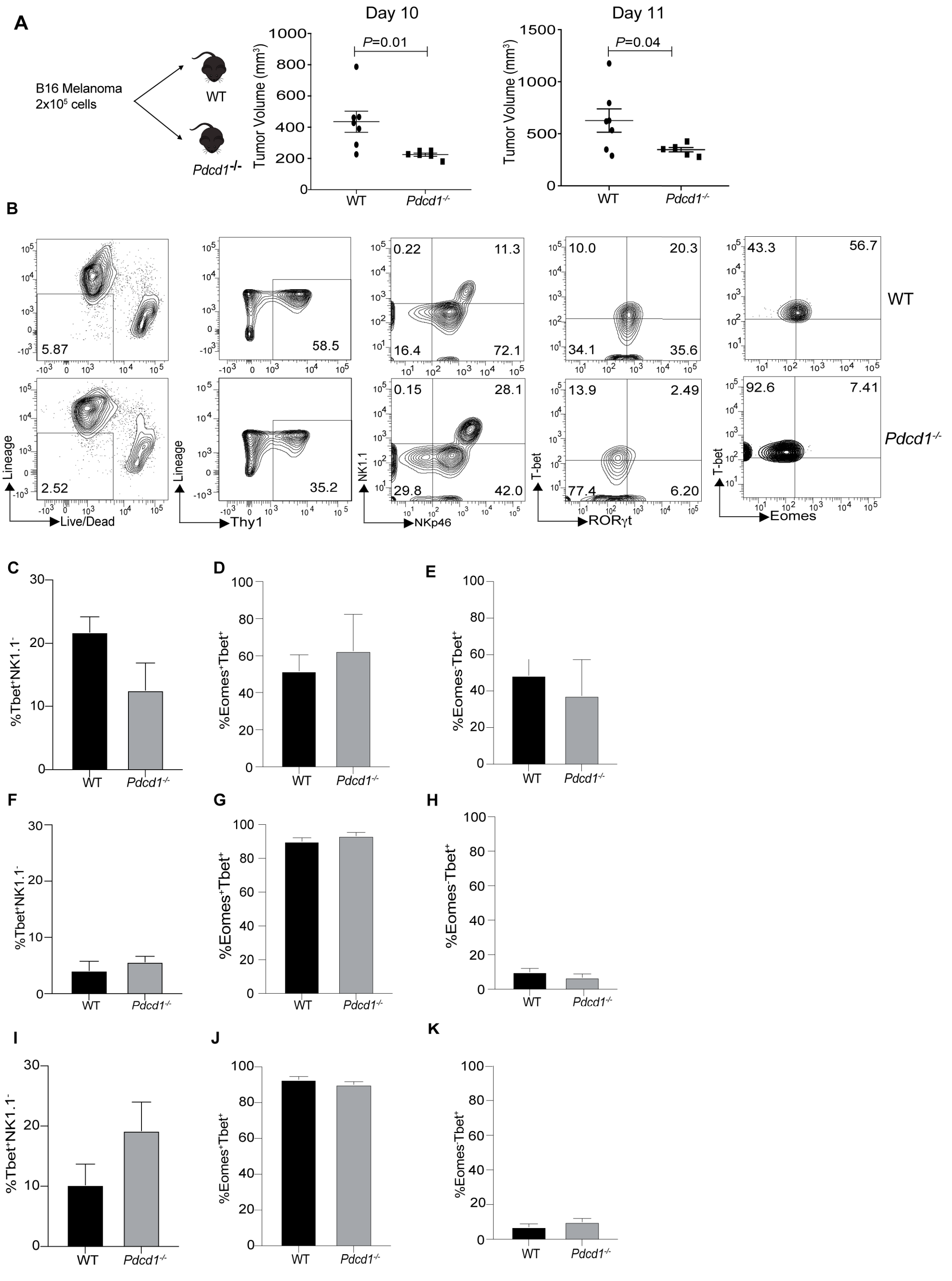

Figure S1: Single Cell Sequencing analysis reveals a novel Tbet^{+}NK1.1^{-} ILC subset within the tumor microenvironment and in normal WT mice.

C57BL6 WT and *pdcd1*$^{-/-}$ mice were reconstituted with B16F10 melanoma cells via subcutaneous injection. Tumor volume was measured at day 10 and day 11 **A**. At day 12, tumors were resected and tumor infiltrating lymphocytes were isolated and then subjected to single cell analysis. WT and PD1 Ko mice liver, bone marrow and spleen were harvested. Tbet^{+}NK1.1- ILCs were characterized as shown in **B**. Frequency of Tbet^{+}NK1.1^{-} ILCs in liver **C**, bone marrow **F** and Spleen **I** is shown. Frequency of Eomes^{+}Tbet^{+} ILCs in liver **D**, bonemarrow **G** and Spleen **J** is shown. Frequency of Eomes^{-}Tbet^{+} ILCs in liver **E**, bonemarrow **H** and Spleen **K** is shown. Data shown are Mean$\pm$SEM of n=4 mice, statistical significance was performed using an unpaired t test. Immunobiology experiments were repeated twice.

Figure S2

Functional Gene Enrichment Analysis of Clusters

Cluster_1

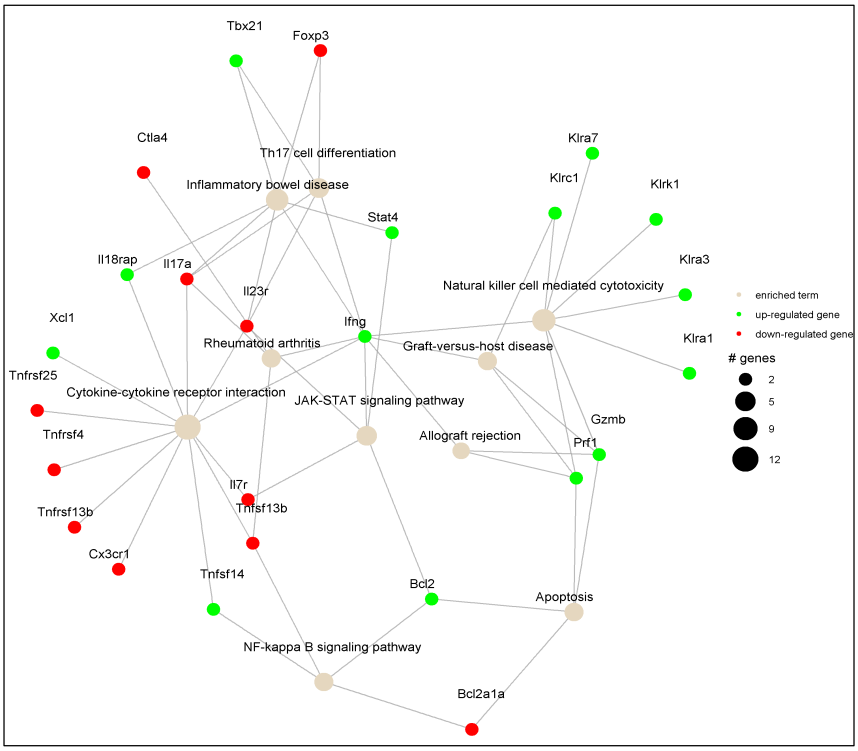

Cluster_2

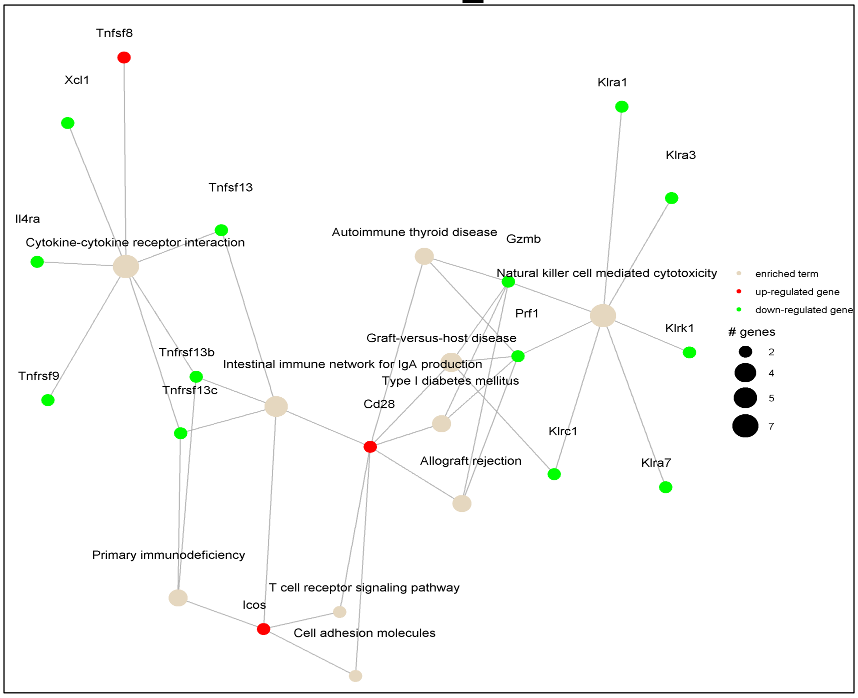

Cluster_3

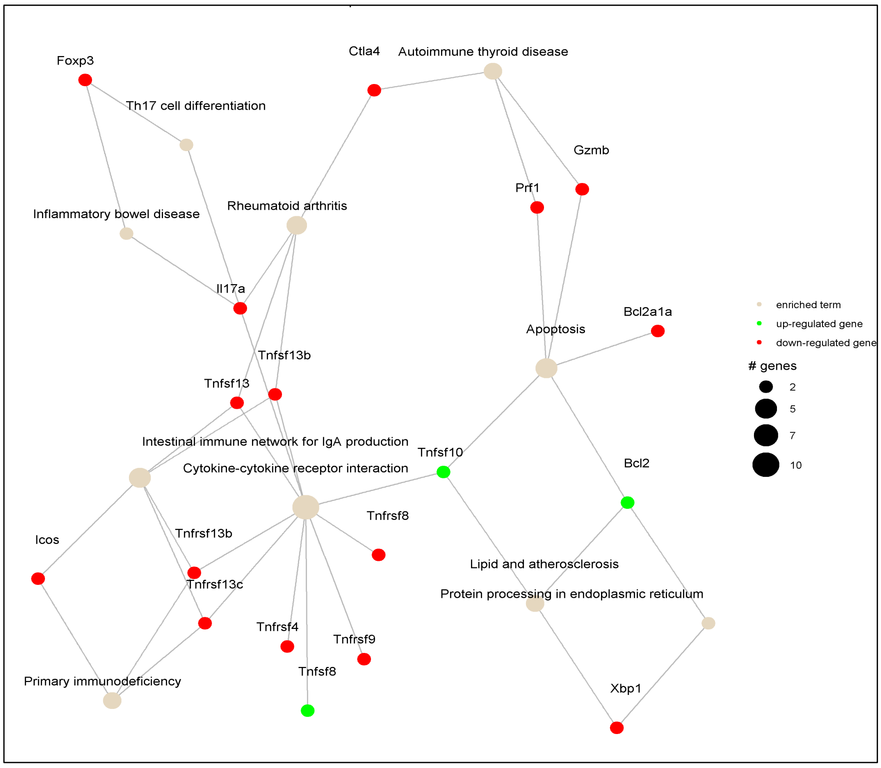

Cluster_4

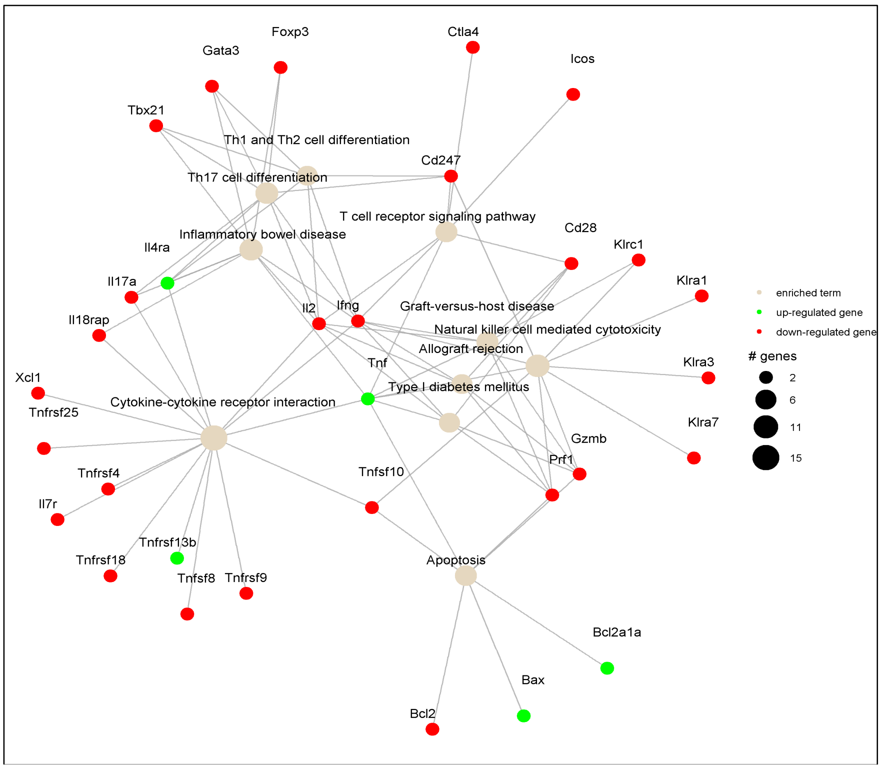

Cluster_5

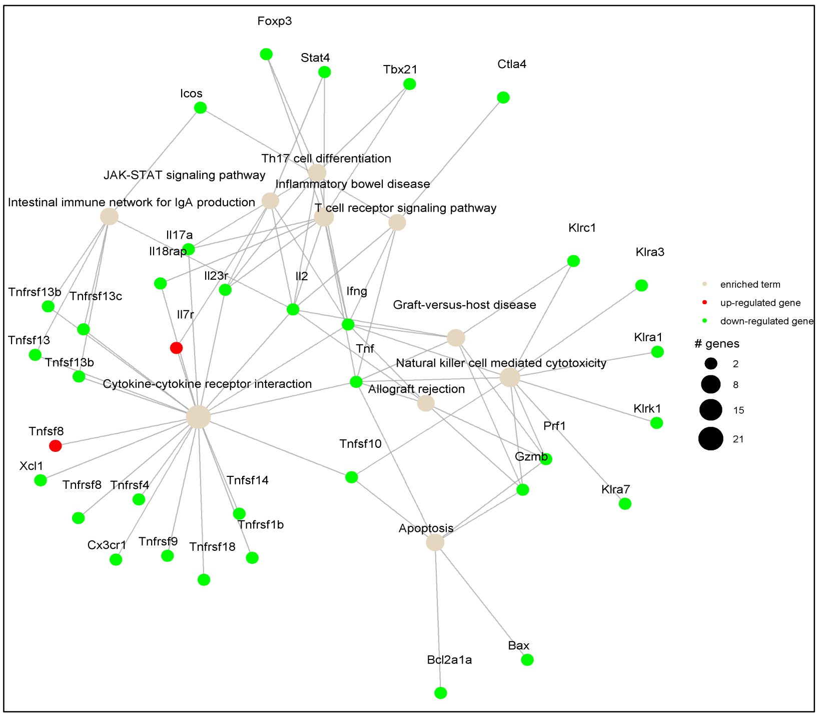

Cluster_6

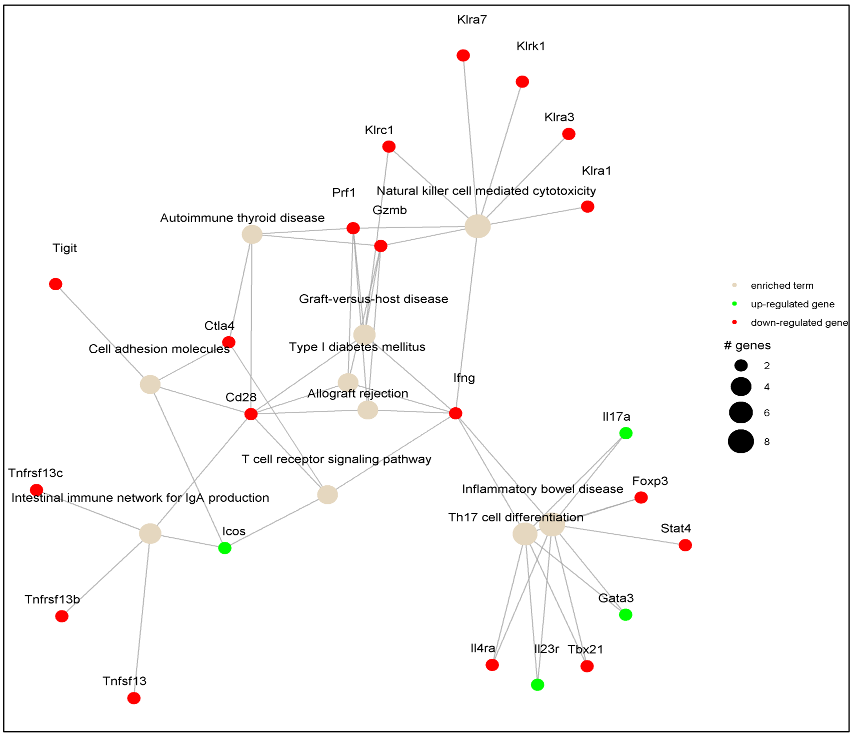

Cluster_7

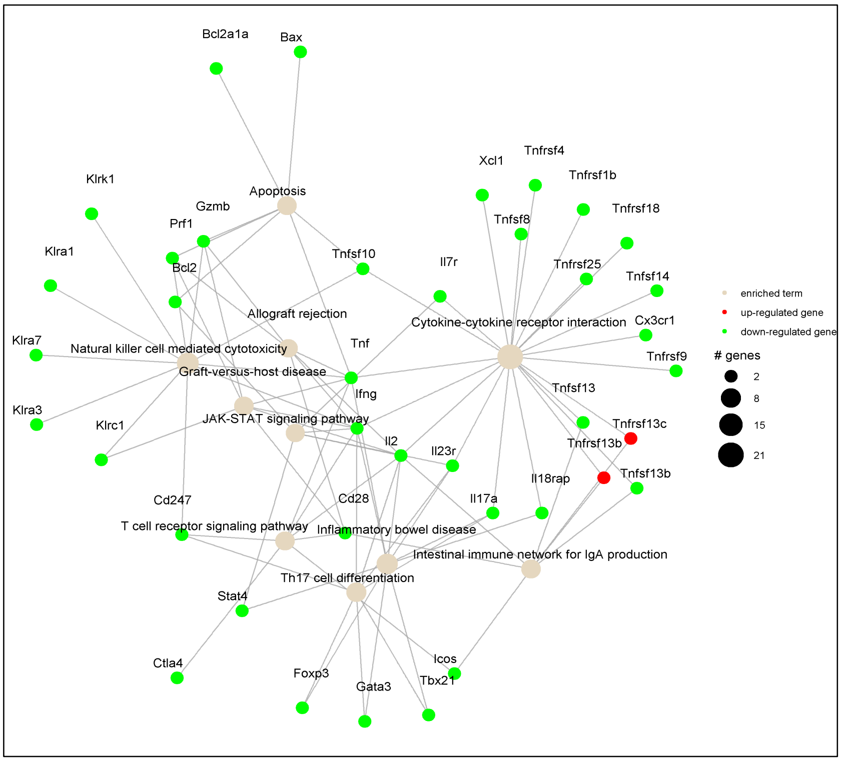

Cluster_8

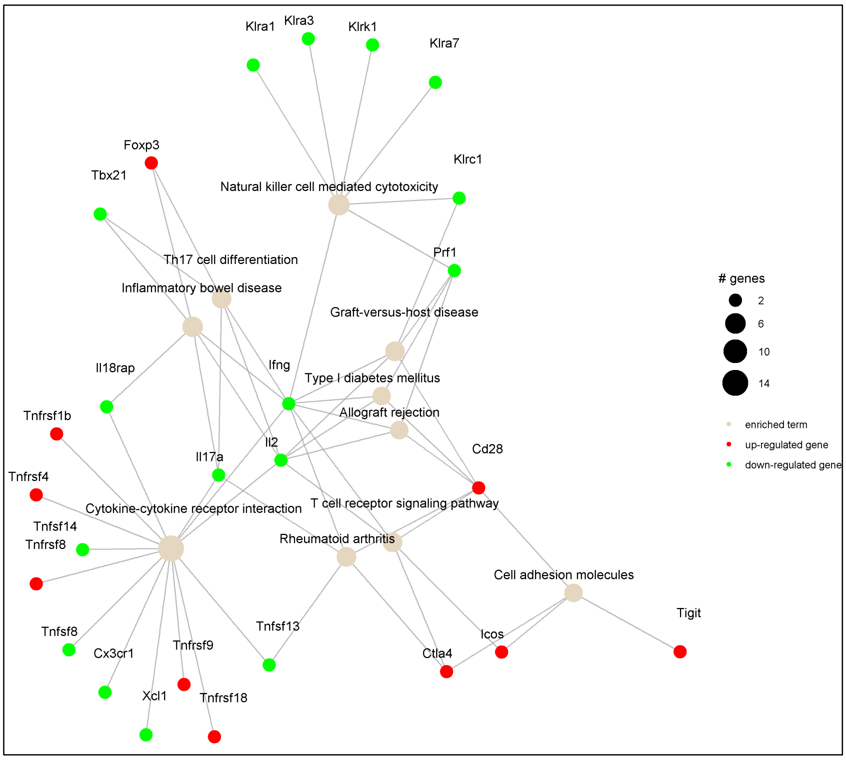

Cluster_9

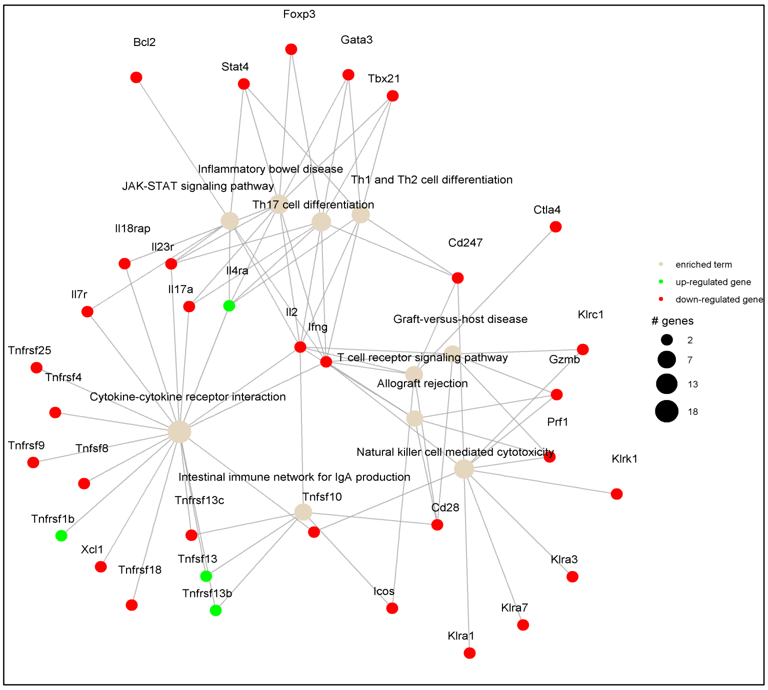

Cluster_10

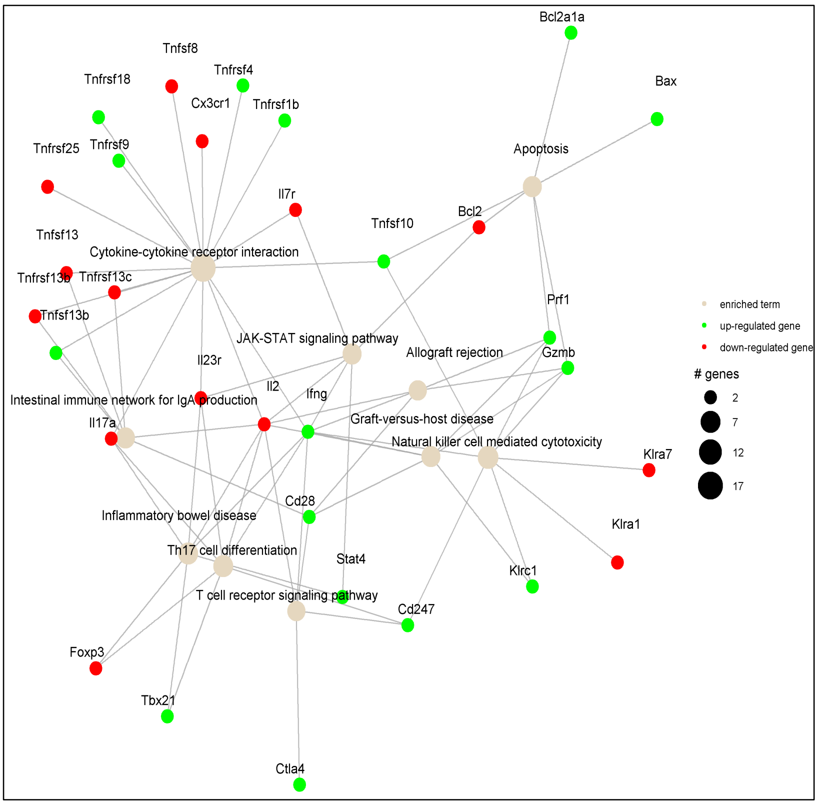

Cluster_11

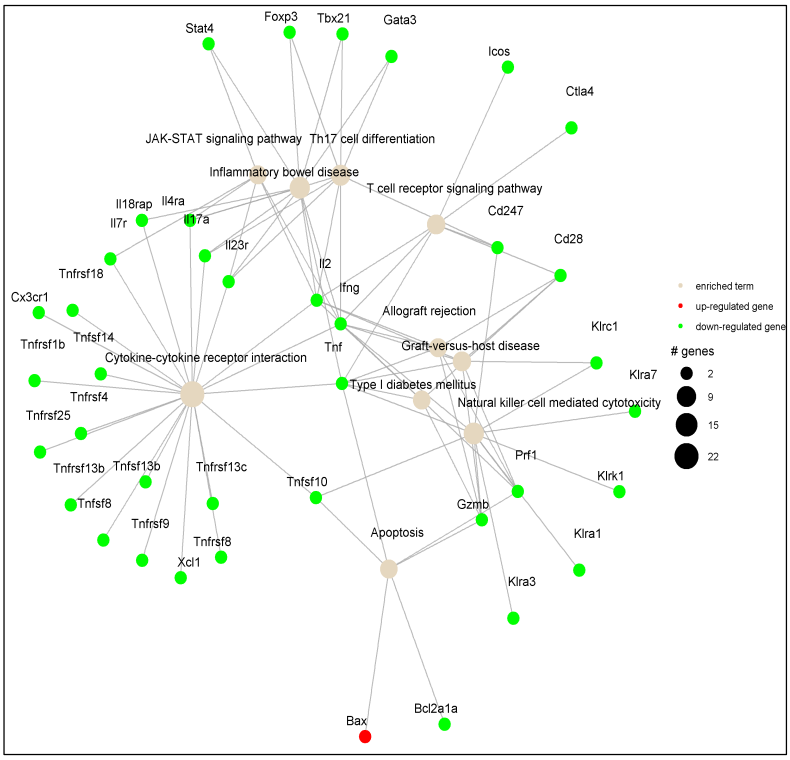

Cluster_12

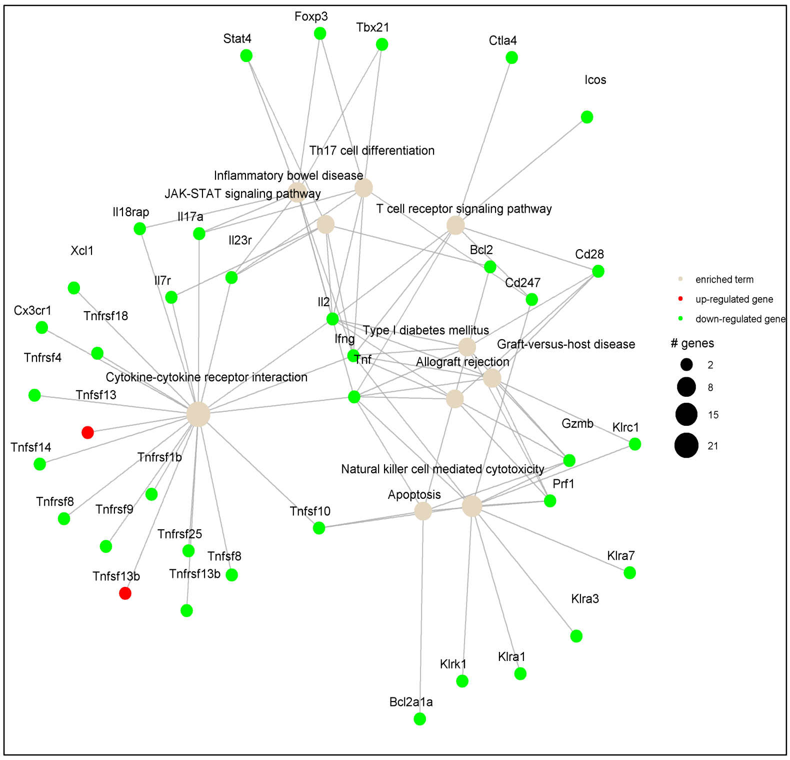

Cluster_13

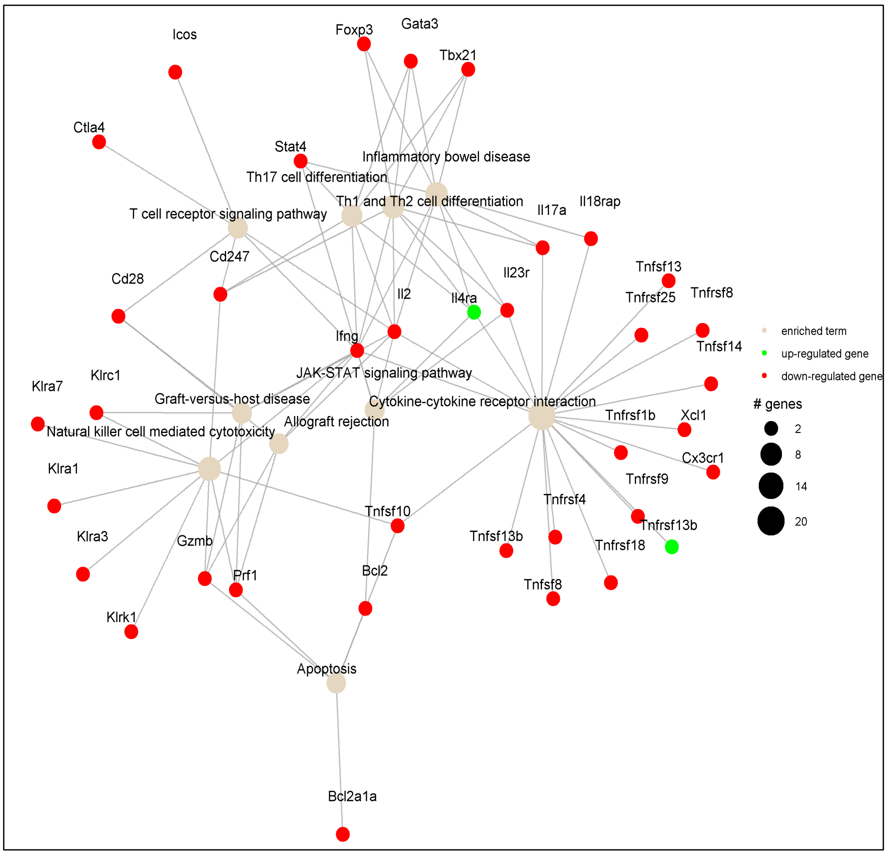

Figure S2: Functional Gene Enrichment analysis of immune populations within the tumor microenvironment

C57BL6 WT and *pdcd1*$^{-/-}$ mice were reconstituted with B16F10 melanoma cells via subcutaneous injection. At day 12, tumors were resected and tumor infiltrating lymphocytes were isolated and then subjected to single cell analysis. Functional Gene Enrichment analysis was performed in all the 13 clusters identified through single cell analysis.

Figure S3

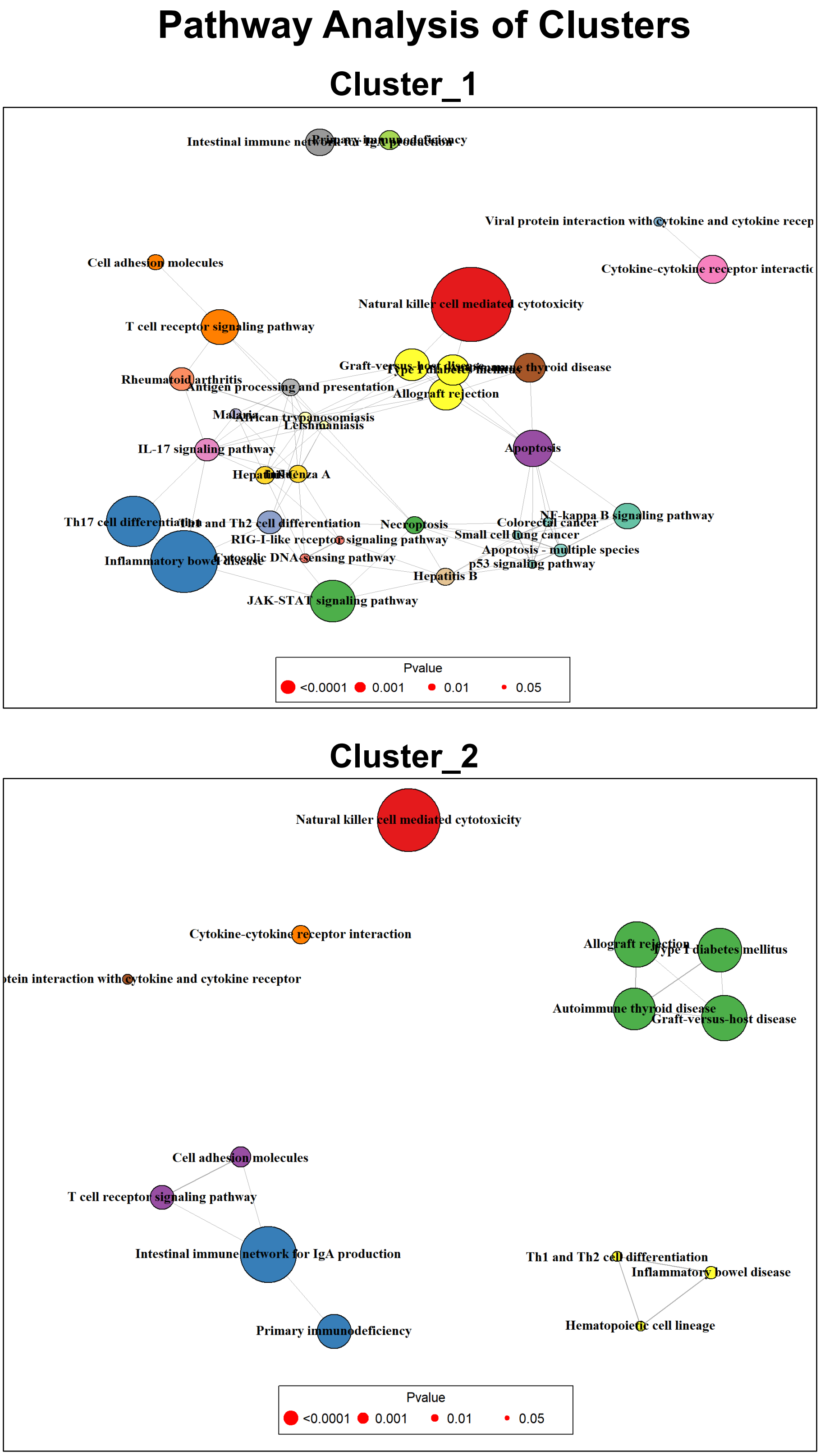

Pathway Analysis of Clusters
Cluster_1
Intestinal immune network for IgA production
Primary immunodeficiency
Viral protein interaction with cytokine and cytokine recep
Cell adhesion molecules
Cytokine-cytokine receptor interactio
Natural killer cell mediated cytotoxicity
T cell receptor signaling pathway
Graft-versus-host disease
Type I diabetes mellitus
Autoimmune thyroid disease
Rheumatoid arthritis
Antigen processing and presentation
Allograft rejection
Malaria
African trypanosomiasis
Leishmaniasis
IL-17 signaling pathway
Apoptosis
Hepatitis C
Influenza A
Th17 cell differentiation
Th1 and Th2 cell differentiation
Necroptosis
Colorectal cancer
NF-kappa B signaling pathway
RIG-I-like receptor signaling pathway
Small cell lung cancer
Apoptosis - multiple species
Inflammatory bowel disease
Cytosolic DNA-sensing pathway
p53 signaling pathway
Hepatitis B
JAK-STAT signaling pathway
Pvalue
<0.0001 0.001 0.01 0.05
Cluster_2
Natural killer cell mediated cytotoxicity
Cytokine-cytokine receptor interaction
Allograft rejection
Type I diabetes mellitus
otein interaction with cytokine and cytokine receptor
Autoimmune thyroid disease
Graft-versus-host disease
Cell adhesion molecules
T cell receptor signaling pathway
Intestinal immune network for IgA production
Th1 and Th2 cell differentiation
Inflammatory bowel disease
Hematopoietic cell lineage
Primary immunodeficiency
Pvalue
<0.0001 0.001 0.01 0.05

Cluster_3

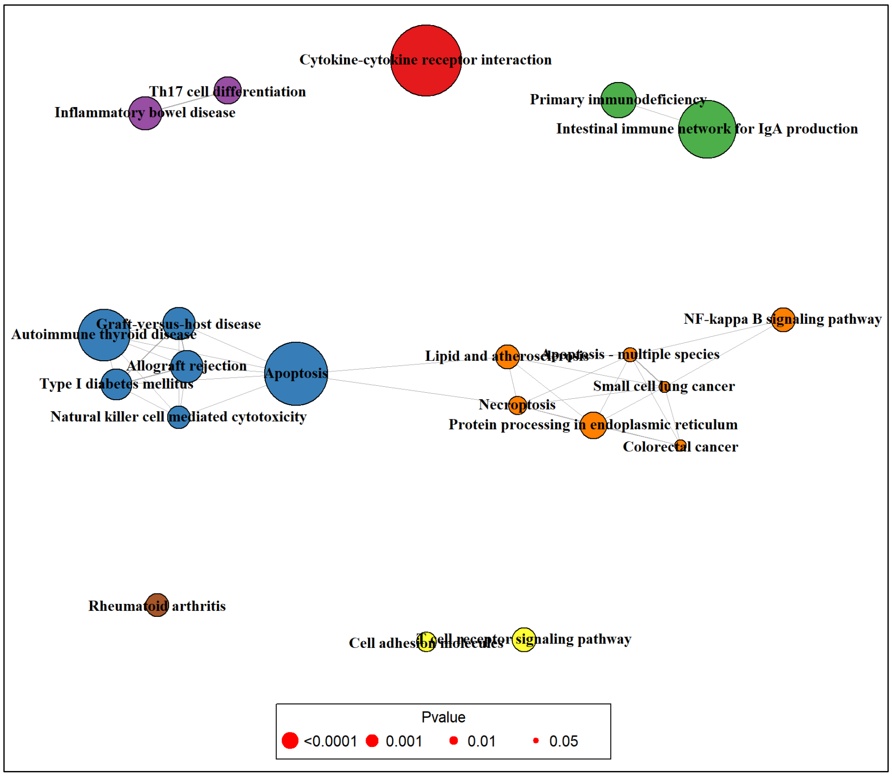

Cluster_4

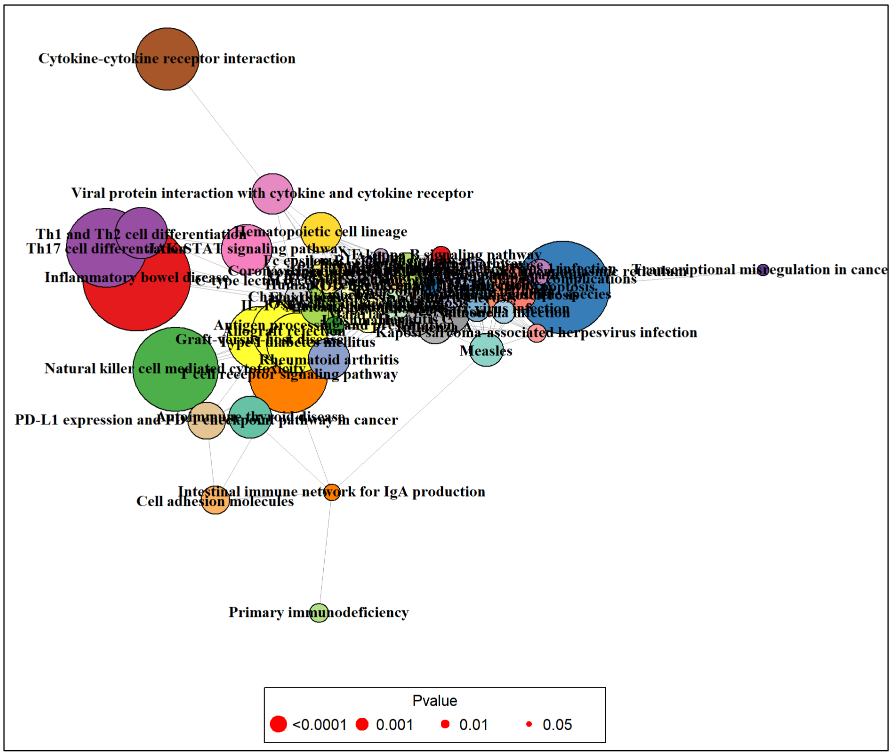

Cluster_5

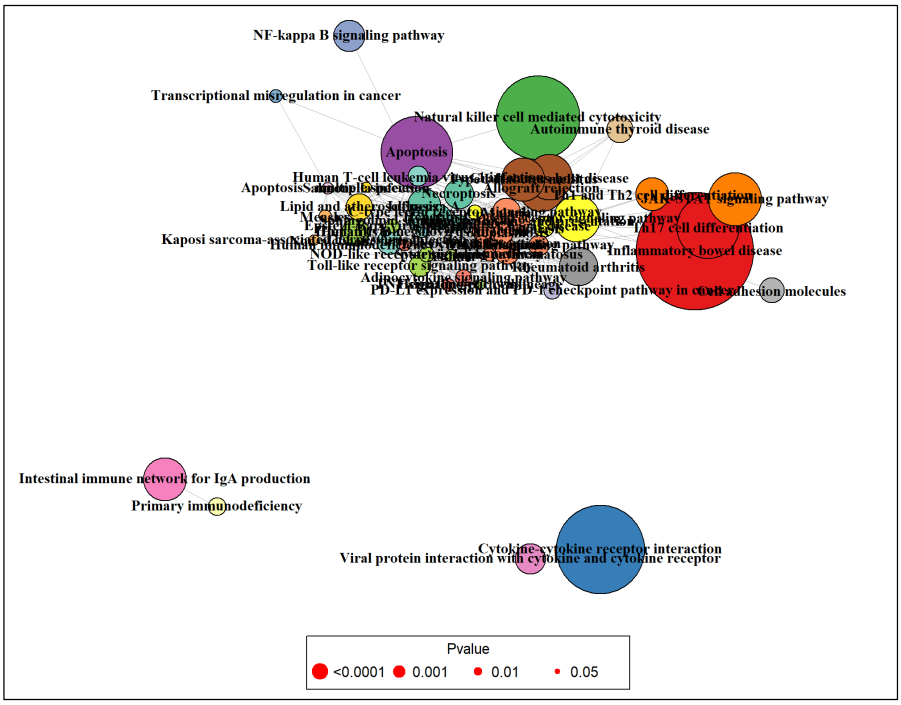

Cluster_6

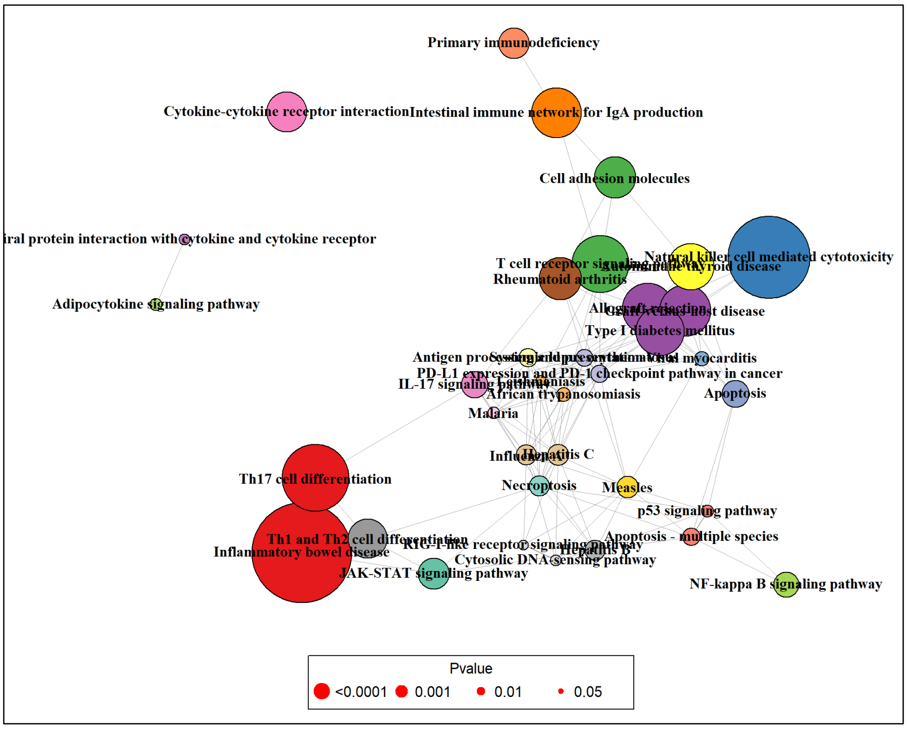

Cluster_7

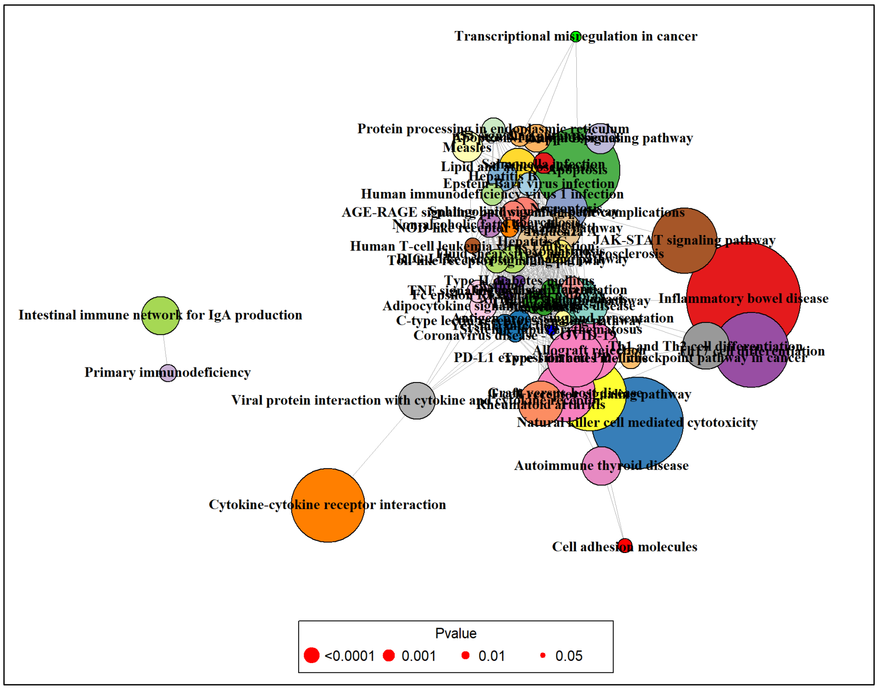

Cluster_8

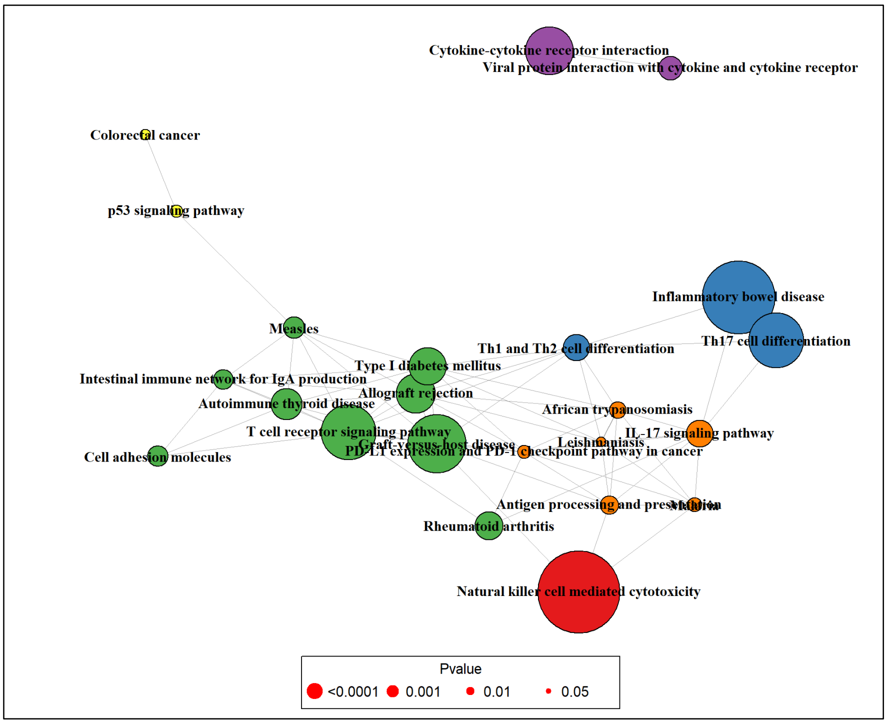

Cluster_9

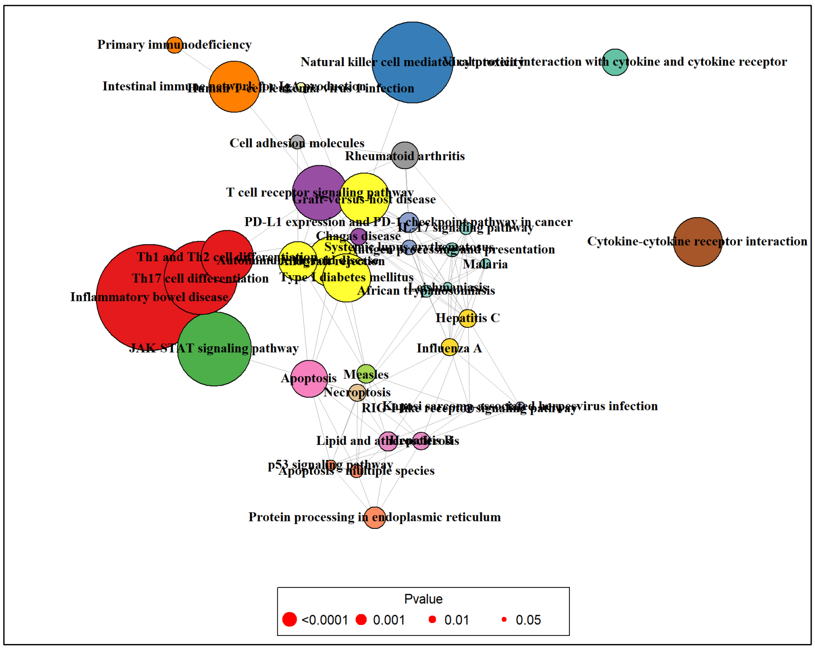

Cluster_10

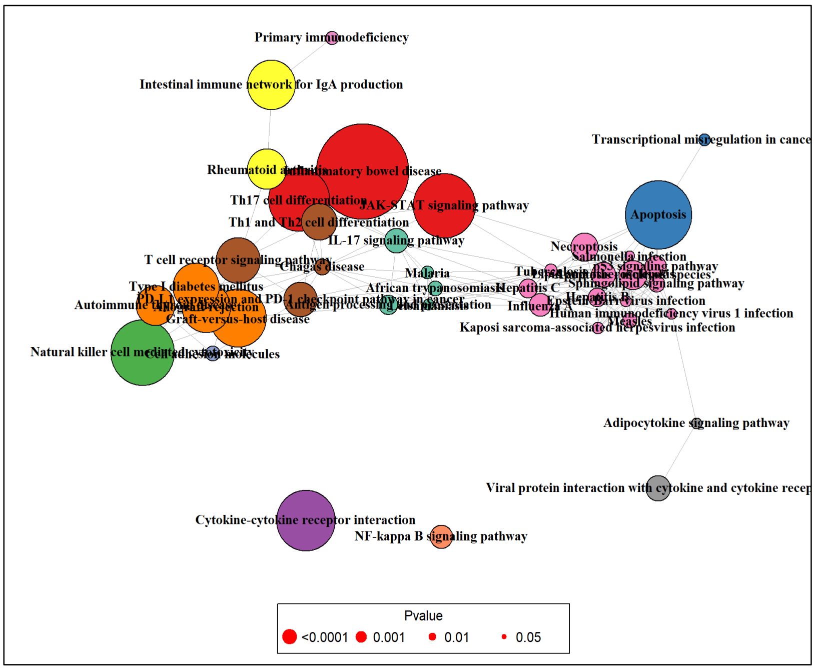

Cluster_11

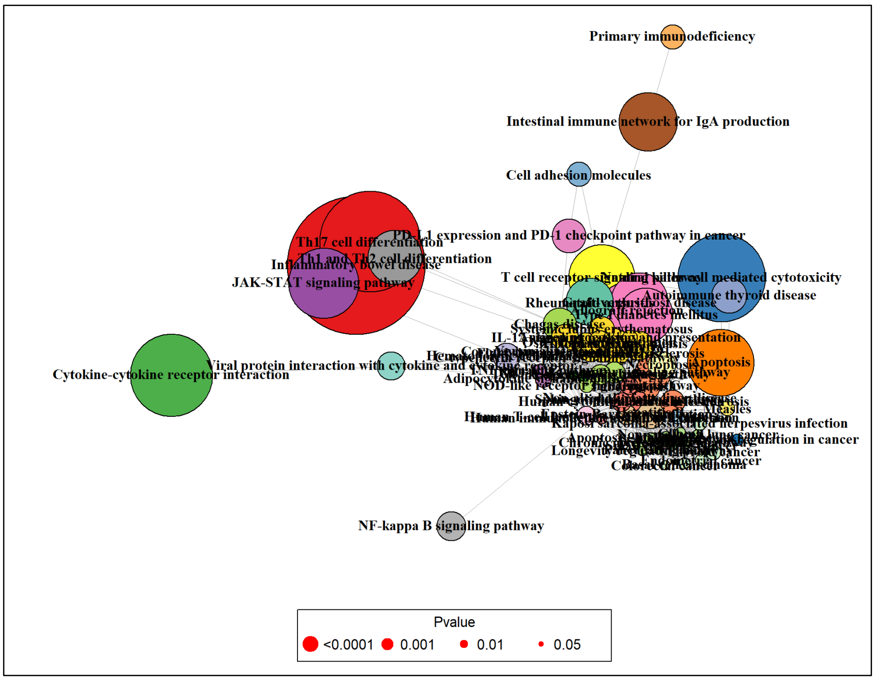

Cluster_12

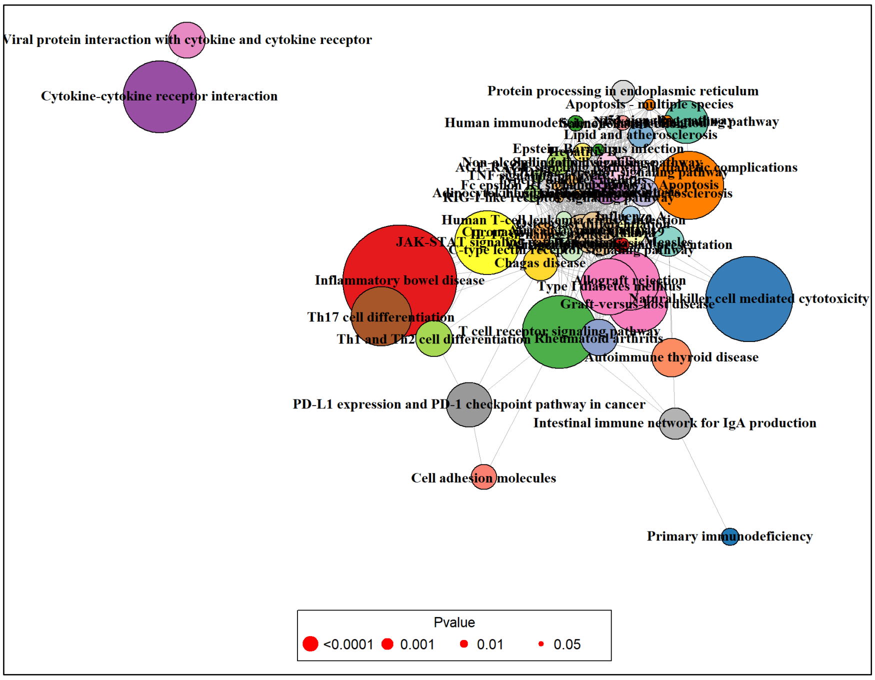

Cluster_13

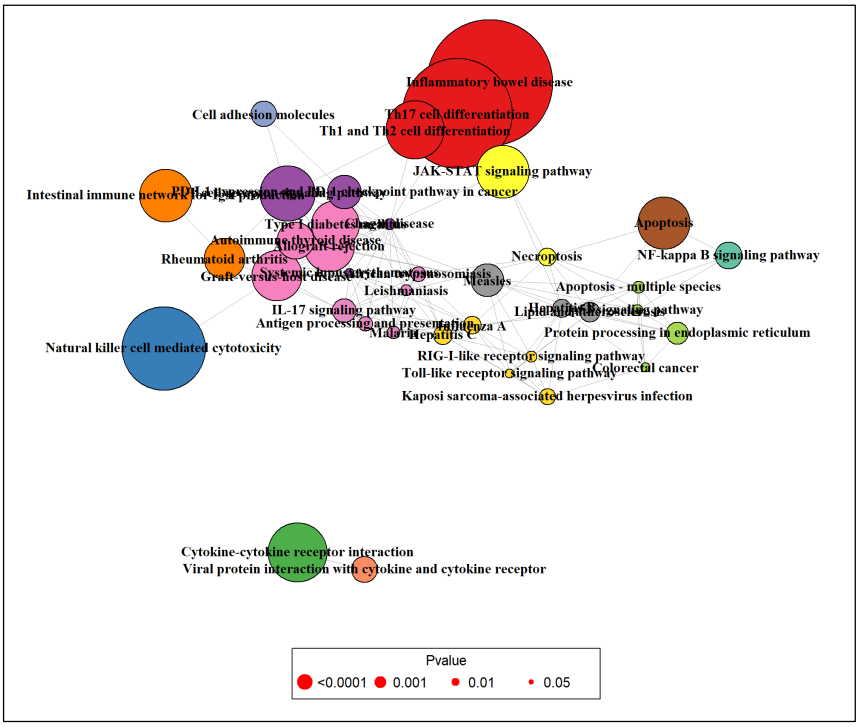

Figure S3: Functional Pathway analysis of immune populations within the tumor microenvironment

C57BL6 WT and *pdcd1*$^{-/-}$ mice were reconstituted with B16F10 melanoma cells via subcutaneous injection. At day 12, tumors were resected and tumor infiltrating lymphocytes were isolated and then subjected to single cell analysis. Functional pathway analysis using KEGG pathways was performed in all the 13 clusters identified through single cell analysis.

Figure S4

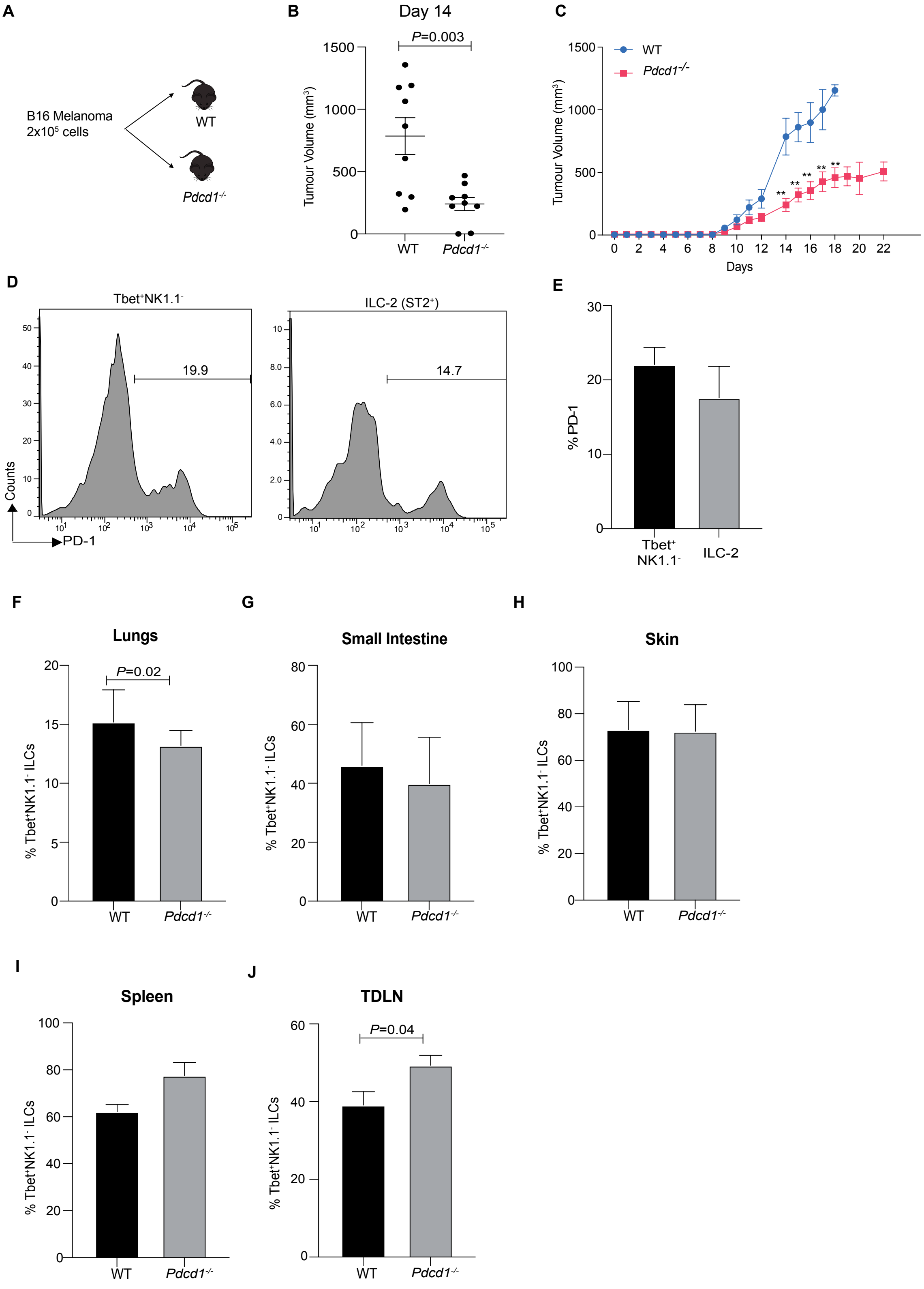

Figure S4: Frequency of Tbet^{+}NK1.1^{-} ILCs in WT and PD1$^{-/-}$ mice within TME

C57BL6 WT or *Pdcd1*$^{-/-}$ mice were reconstituted with B16F10 melanoma cells via subcutaneous injection. Tumor volume was measured in the two cohorts at indicated time

points **A-C**. At day 14, tumors were resected and tumor infiltrating lymphocytes were isolated and characterized by flow cytometry. ILCs were characterized as $Lineage^{-}Thy1^{+}$, lineage gate included antibodies to CD3, CD4, NK1.1, CD49b, CD5, CD8, CD11b, CD11c, CD19, Ter119, F4/80, B220, and Gr1. Representative flow cytometry plot showing PD-1 expression in $Tbet^{+}NK1.1^{-}$ ILCs and ILC2s ($ST2^{+}$) within the TME **D-E**. C57BL6 WT *TbetZsGreen* mice (WT TBG) or *Pdcd1*$^{-/-}$*TbetZsGreen* mice ($PD1^{-/-}$ TBG) were reconstituted with B16 melanoma cells via subcutaneous injection. At day 14, tissues namely spleen, tumor draining lymph nodes (TDLN), small intestine, skin and lungs were resected and lymphocytes were isolated and characterized by flow cytometry. ILCs were characterized as $Lineage^{-}CD45^{+}$, lineage gate included antibodies to CD3, CD4, NK1.1, CD49b, CD5, CD8, CD11b, CD11c, CD19, Ter119, F4/80, B220, and Gr1. Frequency of $Tbet^{+}NK1.1^{-}$ ILC in lungs **F**, Small intestine **G**, skin **H**, spleen **I** and tumor derived lymph nodes **J** is shown. Data shown are Mean$\pm$SEM of n=5 mice, statistical significance was performed using an unpaired t test. Experiment were repeated twice.

Figure S5

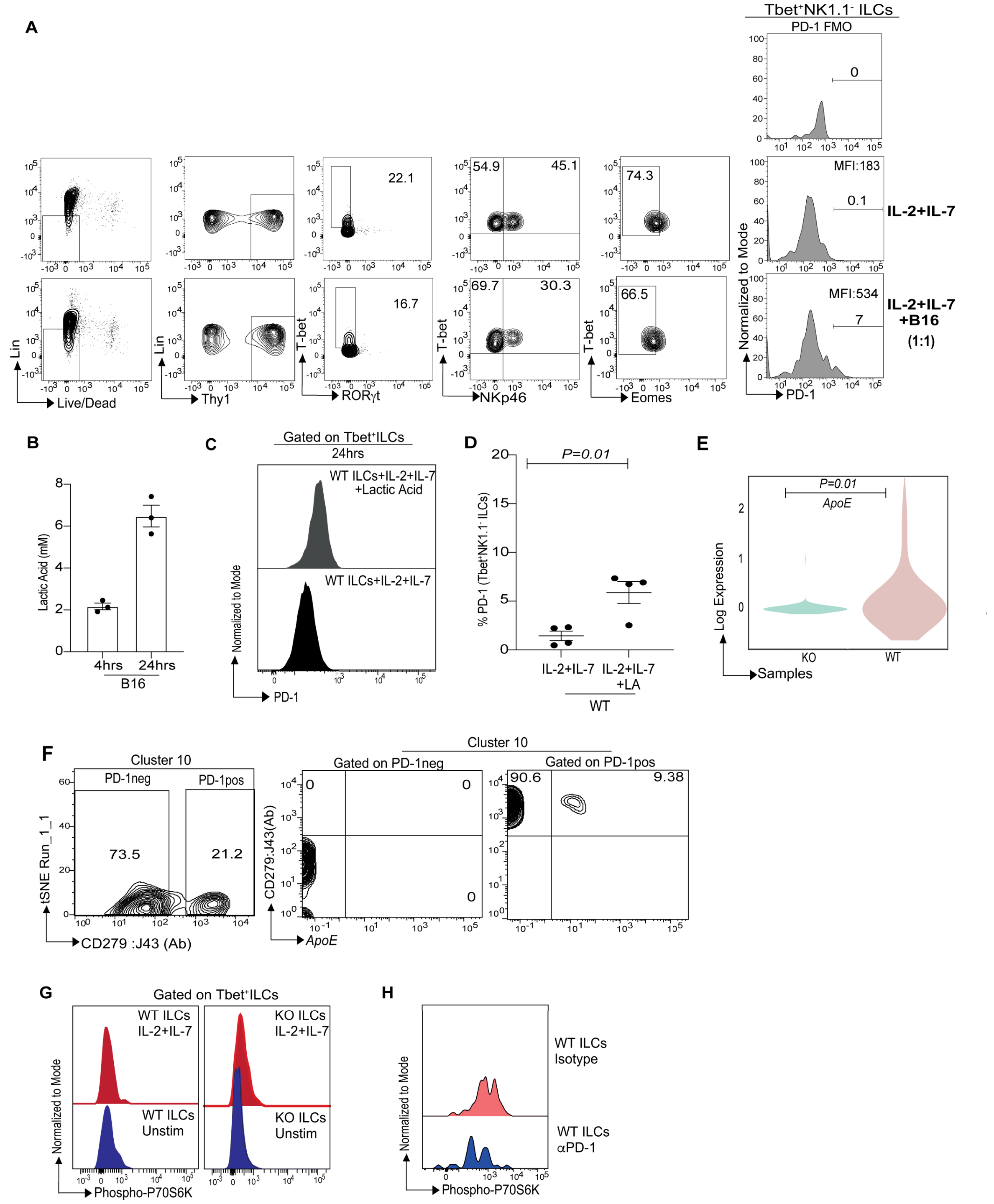

Figure S5: PD-1 mediated molecular mechanism in Tbet^{+}ILC TILs

C57BL6 WT *TbetZsGreen* mice (WT TBG) or *Pdcd1*$^{-/-}$*TbetZsGreen* mice (PD1$^{-/-}$ TBG) were reconstituted with B16 melanoma cells via subcutaneous injection. At day 14, skin and TILs were harvested, and PD-1 expression was measured using flow cytometry **A.** Transwell

experiments were done with B16 melanoma cells at various ratios and then PD-1 expression was monitored in cultures stimulated with either cytokines alone (IL2, IL7) or in the presence of B16 melanoma cell line at 1:1, 1:10 (1 melanoma cell:10 lymphocytes) and 1:100 ratio after 6hrs. ILC characterization and PD-1 protein expression on $Tbet^{+}NK1.1^{-}$ ILCs at 1:1 ratio is shown **B**. B16 melanoma cells were expanded in media for 4hrs or 24 hrs and then supernatant collected. Lactic acid was measured in the supernatant **C**. Splenocytes were incubated for 24hrs with IL2 and IL7 along with lactate. PD-1 expression and frequency on $Tbet^{+}$ILCs were measured **D-E**. *Apo-E* gene transcript in WT and KO $Tbet^{+}$ILCs within TME is shown **F**. TILs were harvested and stimulated with IL2 and IL7 for 15mins and then phosphoP70S6Kinase was measured (**G**, n=4). WT TILs were stimulated for 3 days with either isotype control or anti-PD1 antibody. At day 3, TILs were stimulated for 15 mins with IL2 and IL7 and then phosphoP70S6Kinase was measured (**H**, n=3). Data shown are Mean±SEM, each data point refers to the number of mice per cohort used per experiment, or individual number of *in-vitro* experiments performed. Statistical test was performed using an unpaired t test. *In-vivo* experiments were repeated at-least twice and *in-vitro* experiments were repeated three times.

Figure S6

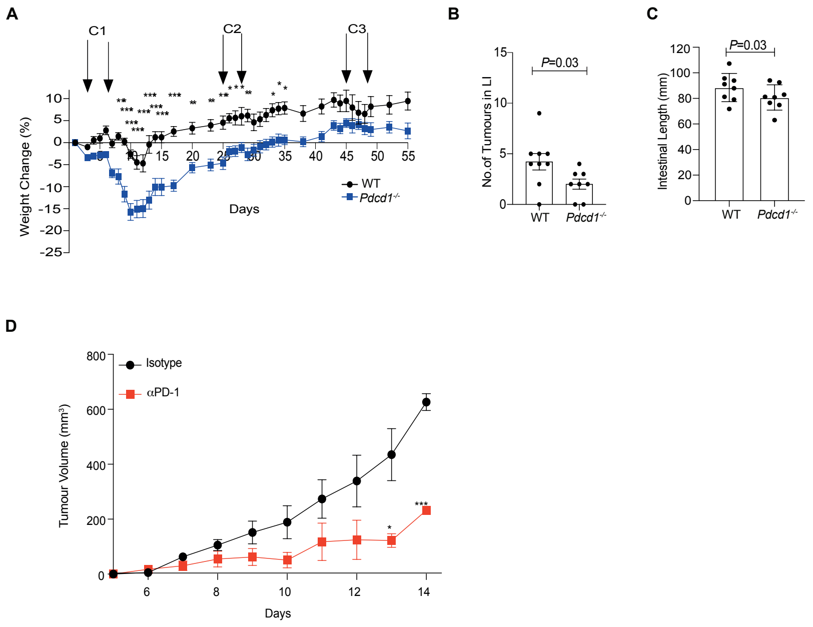

Figure S6: PD-1 deficiency and blockade enhances tumor responses in inducible CRC, subcutaneous melanoma and metastatic melanoma

WT TBG or *Pdcd1*$^{-/-}$TBG were treated with one dose of AOM followed by three cycles of DSS. Weight loss was monitored in the cohorts **A**. Animals were harvested at day 55 and then number of tumors were counted within the intestine **B** and the intestinal length was measured **C**. WT TBG mice were reconstituted with B16F10 melanoma cells via subcutaneous injection. Mice were treated with either isotype control or anti-PD-1 therapy on days 7, 9, 11 and 13. Tumor growth was monitored **D.** Data shown are Mean±SEM, each data point refers to the number of mice per cohort used per experiment. Statistical test was performed using an unpaired t test for comparison of two groups. An ANOVA was performed to determine statistical significance for clinical weight loss.

Figure S7

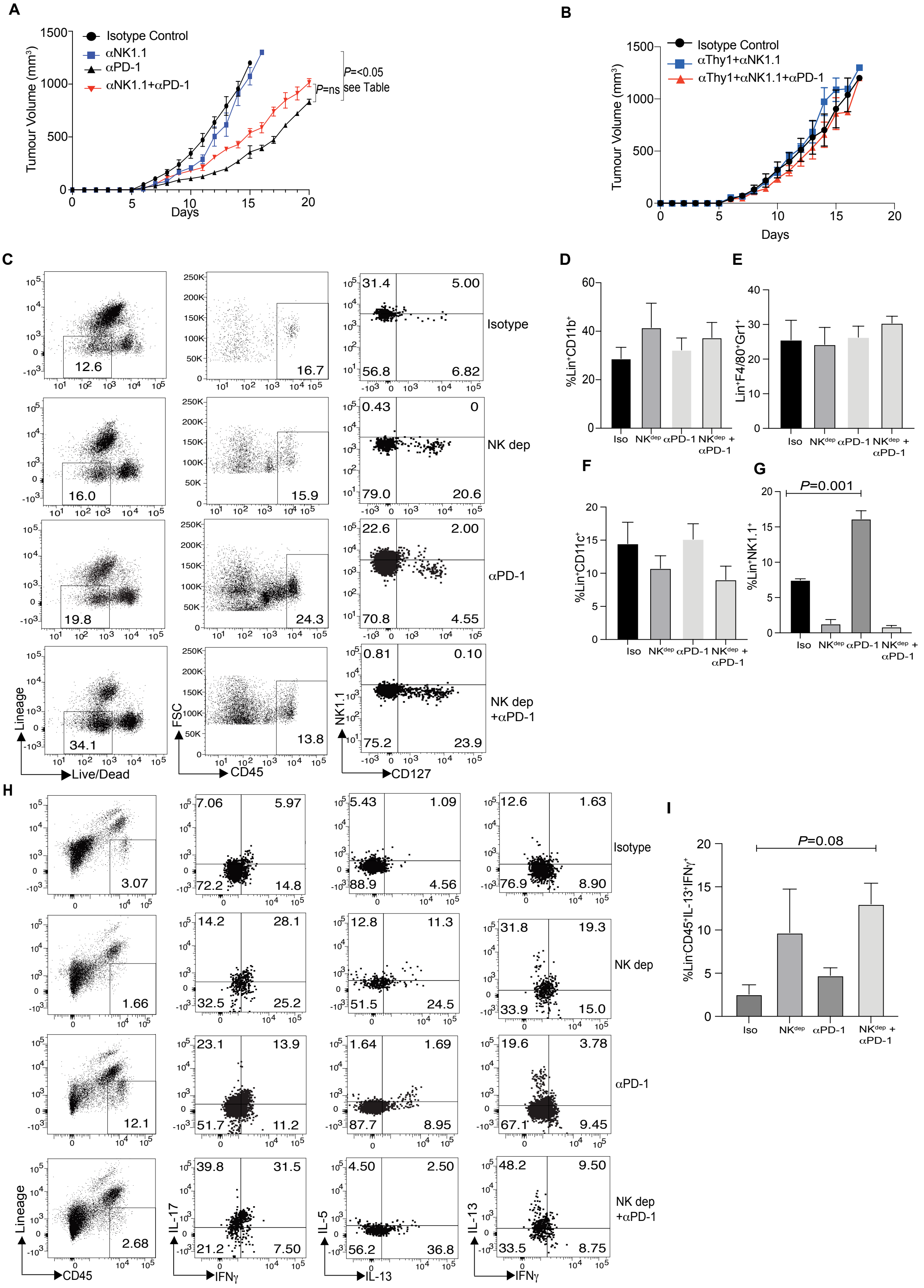

Figure S7: PD-1 blockade significantly enhances the frequency and IFNγ cytokine of helper ILCs within the B16 TME in *Rag* mice

B6 *Rag*$^{-/-}$ mice were reconstituted with B16F10 cells via subcutaneous injection. Mice were treated with either isotype control, NK depleting antibody alone (αNK1.1), anti-PD1 antibody alone (αPD-1) or in combination with anti-PD1 antibody ((αNK1.1plus αPD1). In a second experiment, Thy1 depletion was performed to deplete ILCs and is denoted as αThy1 (αThy1 plus αNK1.1 and αThy1+αNK1.1+αPD1). Tumour growth curves from both experiments is shown in **A-B**. At day 12, tumors were resected and tumor infiltrating lymphocytes were isolated and characterized by flow cytometry. ILCs were characterized as Lineage^{-} CD45^{+}CD127^{+}, lineage gate included antibodies to CD3, CD5, CD8, CD11b, CD11c, CD19, Ter119, F4/80, B220, and Gr1^{+}. Representative flow cytometry plot showing ILC characterization in various cohorts is shown **C**, Frequency of monocytes **D**, macrophages **E**, granulocytes **F** and DCs **G** are shown. In some experiments, tumor infiltrating lymphocytes were stimulated with cytokine stimulation cocktail for 4hrs. Cells were then subjected to intracellular flow cytometry in order to measure effector cytokines. Representative flow cytometry of cytokine profile within helper ILCs are shown **H**. Summary of IL13^{+}IFNγ^{+} helper ILCs is shown **I**. Data shown are Mean+SEM, n=4-5 mice per cohort used per experiment and was repeated twice. Statistical test was performed using a One-way ANOVA analysis for tumor volume and immunobiology analysis. See supplementary Table 3 for statistical significance for cohorts shown in **A**.

Figure S8

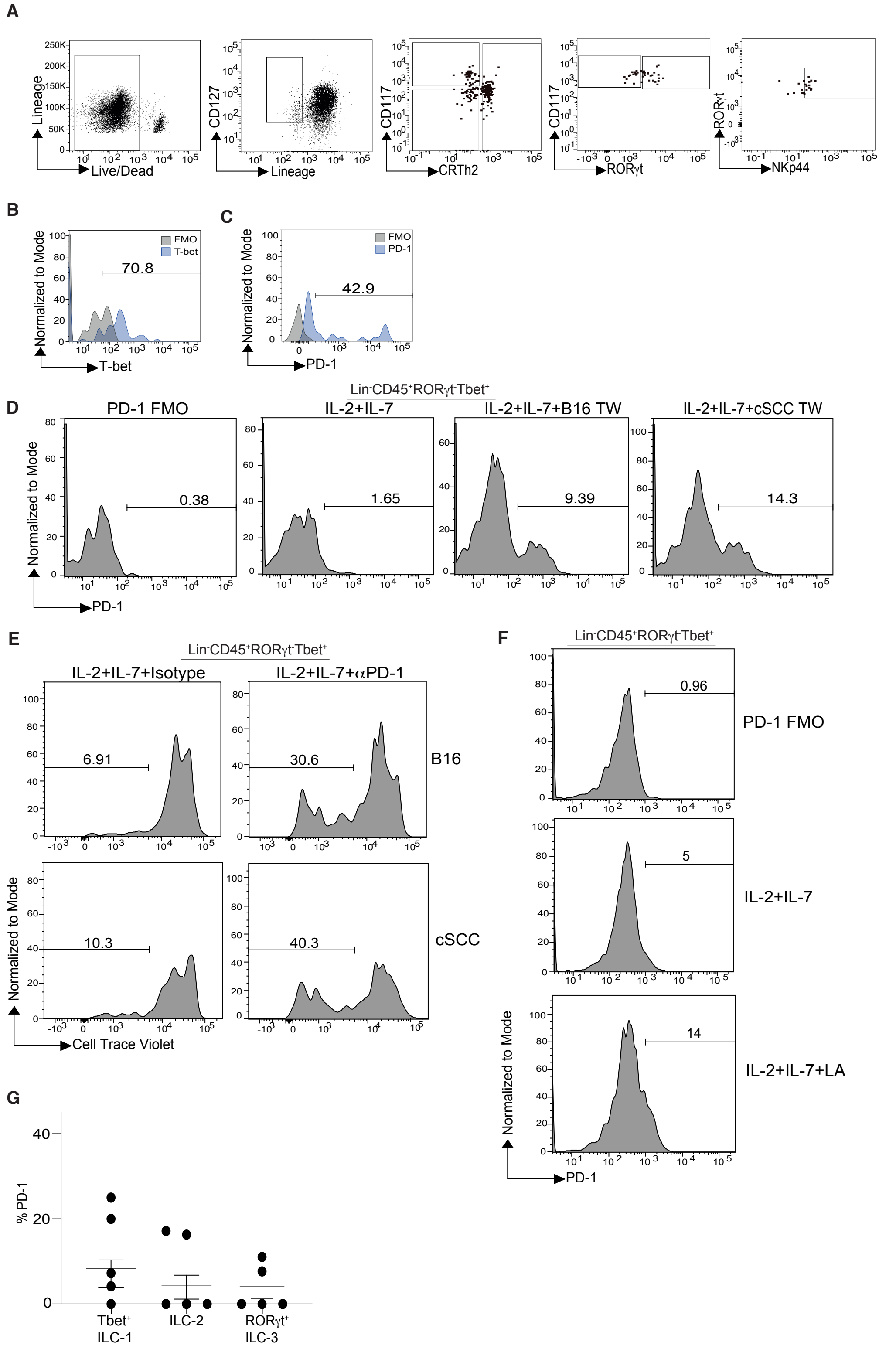

Figure S8: PD-1 regulates human Tbet+ ILC proliferation in the presence of human melanoma and cutaneous squamous cell carcinoma

Human ILC characterization by flow cytometry is shown in **A**. Expression of of Tbet and PD-1 in Tbet^{+} RORγt^{-} ILCs in normal human PBMC is shown **B-C**. PD-1 expression in Tbet^{+} RORγt^{-} ILCs in the presence of human melanoma cell lines and human cSCC cell lines with isotype or anti-PD1 antibody is shown **D**. The rate of proliferation of Tbet^{+} RORγt^{-} ILCs in the presence of human melanoma cell lines and human cSCC cell lines with isotype or anti-PD1 antibody is shown **E**. Representative flow plot showing PD-1 expression in T-BET^{+}ILCs in normal human PBMC samples post LA treatment **F.** Tumor tissue were obtained from cSCC patients and then helper ILC subsets were characterized using flow cytometry. Summary of PD-1 expression in helper ILCs within PBMC is shown **G**. Data shown are Mean$\pm$SEM, n=5 donors.

Dataset S1: Downregulated Genes in Clusters 1-13

Cluster-1

[GeneSet]		
$SchemaVersion	1	
$GeneSetName	Down_in_Phenograph_7FX1_1_RunID:_SAAT	
$GeneSetDescription	Generated in the iCellR Pipeline. Fold-Change < 0.5; q-Val (FDR) < 0.05	
[Genes]	Fold-Change	q-Value
CD103:M290 (Ab)	0.07007233	2.34E-25
CD223 (Ab)	0.2434008	5.94E-20
CD25:PC61 (Ab)	0.21940795	7.96E-24
CD272 (Ab)	0.08804559	2.91E-98
CD278 (Ab)	0.17683366	1.68E-59
CD279:J43 (Ab)	0.22565645	2.01E-34
CD3:145-2C11 (Ab)	0.10955095	6.15E-180
IL-33R (Ab)	0.14195942	1.29E-26
Bcl11a	0	2.61E-17
Bcl2a1a	0.43290813	7.06E-18
Bcl6	0.48718699	3.01E-05
Btla	0.0492828	1.90E-60
Ctla4	0.24090366	5.46E-18
Cx3cr1	0	0.02956251
Foxp3	0.00437864	7.06E-27
Gzmk	0.21265897	2.35E-18
Icos	0.36255038	7.04E-22
Il17a	0	0.00182044
Il23r	0	4.76E-06
Il7r	0.25386826	1.10E-60
Irf7	0.43877988	1.46E-22
Klra17	0	1.06E-12
Lag3	0.16211942	2.89E-22
Tnfrsf13b	0.03786466	3.55E-64
Tnfrsf25	0.43612744	4.96E-05
Tnfrsf4	0.09875554	1.58E-14
Tnfsf13b	0.05020331	4.20E-08

Cluster-2

[GeneSet]		
$SchemaVersion	1	
$GeneSetName	Down_in_Phenograph_7FX1_2_RunID:_SAAT	
$GeneSetDescription	Generated in the iCellR Pipeline. Fold-Change < 0.5; q-Val (FDR) < 0.05	
[Genes]	Fold-Change	q-Value
CD103:M290 (Ab)	0.09605119	1.49E-23
CD272 (Ab)	0.38927845	6.33E-27
CD335 (Ab)	0.22777852	2.23E-61
NK-1.1 (Ab)	0.23304153	1.35E-93
Bach2	0.3484498	1.25E-11
Bcl11a	0	3.86E-17
Bcl6	0.17491572	3.69E-18
Btla	0.46705473	6.14E-08
Cd244	0.01639307	2.63E-50
Eomes	0.36090263	2.51E-18
Gzma	0.30745181	1.10E-20
Gzmb	0.33693947	1.72E-19
Il4ra	0.23845267	8.86E-56
Klra1	0.09846115	5.61E-40
Klra17	0	1.62E-12
Klra21	0.06880302	3.86E-27
Klra3	0.14595118	6.17E-14
Klra5	0.0725078	0.00042933
Klra6	0.10309702	1.51E-22
Klra7	0.0367339	3.39E-97
Klrb1	0	2.42E-07
Klrc1	0.35468583	1.92E-31
Klrc3	0.1488318	0.00813105
Klrk1	0.31343968	1.74E-48
Lag3	0.4755276	5.90E-05
Prf1	0.20039557	1.69E-12
Tnfrsf13b	0.33042276	2.03E-15
Tnfrsf13c	0.21476993	0.00061221
Tnfrsf9	0.39742112	9.12E-12
Tnfsf13	0	2.39E-40
Xbp1	0.29662281	2.31E-63
Xcl1	0.03574471	2.96E-44

Cluster-3

[GeneSet]		
$SchemaVersion	1	
$GeneSetName	Down_in_Phenograph_7FX1_3_RunID:_SAAT	
$GeneSetDescription	Generated in the iCellR Pipeline. Fold-Change < 0.5; q-Val (FDR) < 0.05	
[Genes]	Fold-Change	q-Value
CD223 (Ab)	0.38309673	3.31E-09
CD25:PC61 (Ab)	0.15348238	1.11E-32
CD272 (Ab)	0.31021108	3.96E-33
CD278 (Ab)	0.44307499	6.03E-14
IL-33R (Ab)	0.19912909	2.79E-24
Bcl11a	0.12243611	4.17E-08
Bcl2a1a	0.28782455	6.09E-23
Bcl6	0.39662402	0.00063561
Btla	0.34841717	2.39E-11
Cd244	0.31903669	3.10E-09
Ctla4	0.15538369	1.20E-26
Eomes	0.44925001	4.21E-13
Foxp3	0.04253823	1.23E-21
Gzma	0.04566709	6.71E-95
Gzmb	0.26176409	7.52E-26
Gzmk	0.3077629	1.10E-07
Icos	0.40574321	2.94E-13
Il17a	0	0.0032532
Klra17	0.03502526	8.94E-11
Klrg1	0.04711332	4.39E-34
Lag3	0.18683854	1.04E-17
Prf1	0.05394695	9.56E-25
Tigit	0.29409059	1.66E-27
Tnfrsf13b	0.20459931	6.19E-26
Tnfrsf13c	0.17600191	0.00021826
Tnfrsf4	0.07425495	1.24E-25
Tnfrsf8	0	4.76E-05
Tnfrsf9	0.25801327	1.50E-27
Tnfsf13	0.16122312	4.26E-18
Tnfsf13b	0	4.66E-10
Xbp1	0.27621141	2.98E-63

Cluster 4

[GeneSet]		
$SchemaVersion	1	
$GeneSetName	Down_in_Phenograph_7FX1_4_RunID:_SAAT	
$GeneSetDescription	Generated in the iCellR Pipeline. Fold-Change < 0.5; q-Val (FDR) < 0.05	
[Genes]	Fold-Change	q-Value
CD278 (Ab)	0.48645043	5.38E-15
CD335 (Ab)	0.35463452	2.64E-26
CD3:145-2C11 (Ab)	0.4250569	1.75E-29
NK-1.1 (Ab)	0.37881293	1.27E-31
Bcl2	0.32383875	3.33E-19
Cd247	0.04977086	2.36E-165
Cd28	0.02250884	1.43E-113
Ctla4	0.05026954	8.11E-37
Eomes	0.07005331	2.82E-57
Foxp3	0	3.55E-27
Gata3	0.14295089	1.83E-08
Gzma	0.00857654	1.90E-111
Gzmb	0.01077397	1.10E-66
Gzmk	0.03998416	4.68E-34
Gzmm	0.20443515	0.006822
Icos	0.07778333	4.69E-57
Ifng	0.03488953	1.60E-59
Il17a	0	0.00230209
Il18rap	0.29600251	6.38E-37
Il2	0	0.0131992
Il7r	0.42223122	3.27E-12
Klra1	0.0260981	2.15E-74
Klra21	0.02290659	4.26E-34
Klra3	0.12109853	1.44E-14
Klra6	0.04887524	1.78E-28
Klra7	0.03790176	3.63E-79
Klrb1	0	1.89E-07
Klrc1	0.04047273	1.92E-158
Klrc3	0	2.16E-07
Klrg1	0.06361725	3.79E-28
Prf1	0.07284471	1.36E-20
Tbx21	0.14509233	8.50E-55
Tcf7	0.27414706	6.46E-32

Tigit	0.02941285	7.14E-89
Tnfrsf18	0.35438395	2.07E-26
Tnfrsf25	0.18566864	1.80E-11
Tnfrsf4	0.32241083	2.34E-07
Tnfrsf9	0.29706983	4.65E-15
Tnfsf10	0.43210156	0.02880545
Tnfsf8	0.21605078	2.32E-08
Xcl1	0.06979505	6.88E-28

Cluster-5

[GeneSet]		
$SchemaVersion	1	
$GeneSetName	Down_in_Phenograph_7FX1_5_RunID:_SAAT	
$GeneSetDescription	Generated in the iCellR Pipeline. Fold-Change < 0.5; q-Val (FDR) < 0.05	
[Genes]	Fold-Change	q-Value
CD119 (Ab)	0.48255359	1.72E-51
CD223 (Ab)	0.18085805	1.25E-26
CD25:PC61 (Ab)	0.0957435	5.55E-38
CD274 (Ab)	0.49107948	9.81E-41
CD279:J43 (Ab)	0.2084539	7.87E-38
CD335 (Ab)	0.25329199	1.40E-45
IL-33R (Ab)	0.21157824	1.76E-20
NK-1.1 (Ab)	0.13490557	9.44E-114
Bax	0.45641447	5.51E-20
Bcl11a	0	2.62E-17
Bcl2a1a	0.05860396	7.12E-81
Bcl6	0.28297469	5.27E-07
Cd244	0	1.84E-55
Cd274	0.33220854	1.21E-13
Ctla4	0.13263731	1.24E-25
Cx3cr1	0	0.02180848
Eomes	0.08728454	7.77E-70
Foxp3	0.07156805	7.48E-19
Gzma	0.00320382	2.40E-113
Gzmb	0	6.84E-69
Gzmk	0	2.60E-41
Gzmm	0.25472069	0.02767491
Icos	0.42682927	5.84E-10

Ifng	0.02894553	7.44E-60
Il17a	0	0.0015880 5
Il18rap	0.03449188	3.86E-209
Il2	0	0.0089767 4
Il23r	0	3.94E-06
Irf7	0.41012236	2.63E-13
Klra1	0	2.38E-83
Klra17	0	1.00E-12
Klra21	0	3.43E-39
Klra3	0	3.85E-28
Klra5	0	6.89E-06
Klra6	0	2.67E-38
Klra7	0.01471734	4.44E-106
Klrb1	0	1.46E-07
Klrc1	0.01005135	7.86E-196
Klrc3	0	1.65E-07
Klrg1	0	3.83E-42
Klrk1	0	2.14E-255
Lag3	0.0122614	9.32E-37
Prf1	0.09076255	5.09E-17
Stat4	0.42716684	3.51E-26
Tbx21	0	8.43E-178
Tigit	0.02929997	2.97E-87
Tnf	0.44528241	0.0022791 6
Tnfrsf13b	0.40897386	2.33E-08
Tnfrsf13c	0	2.75E-13
Tnfrsf18	0.25123012	1.17E-58
Tnfrsf1b	0.11084437	1.26E-94
Tnfrsf4	0.11892081	5.54E-20
Tnfrsf8	0	2.54E-05
Tnfrsf9	0.02998611	5.19E-66
Tnfsf10	0.28772094	6.42E-06
Tnfsf13	0	2.20E-40
Tnfsf13b	0	2.93E-10
Tnfsf14	0.12567122	5.84E-16
Xbp1	0.17055517	3.10E-86
Xcl1	0.0073517	1.28E-48

Cluster-6

[GeneSet]		
$SchemaVersion	1	
$GeneSetName	Down_in_Phenograph_7FX1_6_RunID:_SAAT	
$GeneSetDescription	Generated in the iCellR Pipeline. Fold-Change < 0.5; q-Val (FDR) < 0.05	
[Genes]	Fold-Change	q-Value
CD223 (Ab)	0.477621385	1.77E-05
CD272 (Ab)	0.369822338	2.47E-14
CD335 (Ab)	0.251945122	1.67E-33
NK-1.1 (Ab)	0.23074987	1.18E-83
Bach2	0.445230969	0.002260724
Bcl11a	0	3.96E-17
Bcl2	0.323520374	2.18E-12
Bcl6	0.252510393	2.63E-05
Btla	0.230815838	6.23E-16
Cd244	0.352618064	0.000631347
Cd28	0.484491486	0.000376448
Ctla4	0.320208316	4.61E-10
Cx3cr1	0	0.031157409
Eomes	0.021828737	1.81E-102
Foxp3	0.158250064	1.31E-09
Gzma	0.00537353	1.85E-112
Gzmb	0.282123263	0.004279979
Gzmk	0	3.42E-41
Gzmm	0	1.72E-08
Ifng	0.143004286	1.85E-26
Il4ra	0.34793895	4.47E-21
Irf7	0.435062834	3.00E-11
Klra1	0.020422362	3.79E-74
Klra17	0	1.57E-12
Klra21	0.144641876	8.72E-06
Klra3	0.189784882	1.66E-06
Klra5	0	1.02E-05
Klra6	0.192980741	3.48E-06
Klra7	0.014805578	7.25E-105
Klrb1	0	2.19E-07
Klrc1	0.104307145	8.43E-90
Klrc3	0	2.51E-07
Klrk1	0.318053966	1.67E-28
Lag3	0.046363423	8.97E-29

Prf1	0	3.59E-34
Stat4	0.454725293	8.46E-18
Tbx21	0.184756759	8.12E-34
Tigit	0.055349027	3.63E-63
Tnfrsf13b	0.493969271	0.000268192
Tnfrsf13c	0	4.32E-13
Tnfrsf1b	0.468110114	1.46E-10
Tnfrsf4	0.340831263	1.62E-05
Tnfrsf8	0	3.39E-05
Tnfrsf9	0.147223943	1.68E-33
Tnfsf13	0	2.96E-40
Xcl1	0.018505536	1.17E-46

Cluster-7

[GeneSet]		
$SchemaVersion	1	
$GeneSetName	Down_in_Phenograph_7FX1_7_RunID:_SAAT	
$GeneSetDescription	Generated in the iCellR Pipeline. Fold-Change < 0.5; q-Val (FDR) < 0.05	
[Genes]	Fold-Change	q-Value
CD103:M290 (Ab)	0.05357655	3.01E-28
CD119 (Ab)	0.35309506	3.26E-55
CD223 (Ab)	0.21895448	2.28E-21
CD25:PC61 (Ab)	0.10684951	4.71E-35
CD278 (Ab)	0.2243117	2.95E-43
CD335 (Ab)	0.176686	2.89E-80
CD3:145-2C11 (Ab)	0.32543753	1.62E-22
IL-33R (Ab)	0.22293394	3.86E-20
NK-1.1 (Ab)	0.16195724	1.18E-83
Bax	0.35476775	8.18E-19
Bcl2	0.30624476	4.13E-11
Bcl2a1a	0.24019952	5.75E-18
Cd244	0	2.21E-55
Cd247	0.04358135	9.77E-135
Cd28	0.11238673	1.01E-38
Ctla4	0.02810892	3.64E-39
Cx3cr1	0	0.02492794
Eomes	0.04459922	3.03E-77
Foxp3	0	3.09E-27
Gata3	0	4.07E-26

Gzma	0.00639937	3.36E-112
Gzmb	0.0095695	5.71E-67
Gzmk	0.02125553	1.94E-36
Icos	0.03532714	3.27E-69
Ifng	0.0247471	1.63E-58
Il17a	0	0.0015093
Il18rap	0.0946454	6.54E-60
Il2	0	0.0095070<br>9
Il23r	0	3.50E-06
Il7r	0.19904567	2.61E-16
Irf7	0.2161969	2.76E-27
Klra1	0.05566995	3.19E-56
Klra17	0	8.71E-13
Klra21	0.14729621	8.39E-09
Klra3	0	3.91E-28
Klra6	0.10425593	2.95E-10
Klra7	0.08084835	1.85E-49
Klrc1	0.06208539	3.20E-85
Klrg1	0.01927545	8.14E-38
Klrk1	0.07262443	1.68E-80
Lag3	0.02099149	2.29E-34
Prf1	0.0773961	1.23E-14
Stat4	0.33995654	1.79E-22
Tbx21	0.0679702	1.76E-84
Tcf7	0.1547922	1.56E-47
Tigit	0.02504976	1.14E-83
Tnf	0.12319428	9.67E-24
Tnfrsf18	0.09031359	1.27E-92
Tnfrsf1b	0.25381215	1.09E-26
Tnfrsf25	0	1.13E-39
Tnfrsf4	0.05051763	1.09E-26
Tnfrsf9	0.00640283	3.90E-74
Tnfsf10	0.08046737	2.21E-15
Tnfsf13	0.05056802	3.29E-24
Tnfsf13b	0	2.36E-10
Tnfsf14	0	5.86E-51
Tnfsf8	0.12957047	1.91E-10
Xbp1	0.23542791	4.59E-36
Xcl1	0.02676415	1.68E-44

Cluster 8

[GeneSet]		
$SchemaVersion	1	
$GeneSetName	Down_in_Phenograph_7FX1_8_RunID:_SAAT	
$GeneSetDescription	Generated in the iCellR Pipeline. Fold-Change < 0.5; q-Val (FDR) < 0.05	
[Genes]	Fold-Change	q-Value
CD272 (Ab)	0.47104535	6.47E-16
CD335 (Ab)	0.31640429	1.44E-33
NK-1.1 (Ab)	0.25066293	1.02E-42
Bach2	0.0468517	1.72E-36
Bcl11a	0	3.92E-17
Bcl2	0.1569582	1.07E-26
Bcl6	0.35496116	0.00640618
Cd244	0	2.58E-55
Cx3cr1	0	0.04518221
Eomes	0.01533141	2.43E-103
Gzma	0.00755373	1.68E-111
Gzmk	0.11022478	2.87E-19
Gzmm	0	1.83E-08
Ifng	0.32666269	1.23E-05
Il17a	0	0.00262147
Il18rap	0.22122579	4.75E-43
Il2	0	0.0163735
Klra1	0	3.57E-83
Klra17	0	1.52E-12
Klra21	0	4.80E-39
Klra3	0	5.33E-28
Klra5	0	1.16E-05
Klra6	0	3.73E-38
Klra7	0.00519928	1.37E-109
Klrb1	0	2.24E-07
Klrc1	0.05340711	2.15E-51
Klrc3	0	2.57E-07
Klrk1	0.01068233	2.02E-216
Prf1	0.16048052	0.00015471
Tbx21	0.48925985	5.37E-05
Tcf7	0.1824445	5.76E-27

Tnfsf13	0	3.08E-40
Tnfsf14	0.11051561	9.56E-10
Tnfsf8	0.2013722	1.93E-06
Xcl1	0.10280432	2.12E-13

Cluster-9

[GeneSet]		
$SchemaVersion	1	
$GeneSetName	Down_in_Phenograph_7FX1_9_RunID:_SAAT	
$GeneSetDescription	Generated in the iCellR Pipeline. Fold-Change < 0.5; q-Val (FDR) < 0.05	
[Genes]	Fold-Change	q-Value
CD103:M290 (Ab)	0.19467315	1.17E-14
CD223 (Ab)	0.45794289	1.35E-06
CD25:PC61 (Ab)	0.19836913	9.94E-26
CD274 (Ab)	0.43836219	6.65E-10
CD335 (Ab)	0.36585416	3.05E-21
NK-1.1 (Ab)	0.42384659	2.60E-16
Bach2	0.40208333	0.00514583
Bcl2	0.35485587	5.48E-07
Cd247	0.17976512	9.38E-31
Cd28	0.26197792	4.17E-05
Ctla4	0.02105673	1.91E-39
Eomes	0	5.85E-121
Foxp3	0	4.03E-27
Gata3	0	5.47E-26
Gzma	0.02056664	3.15E-89
Gzmb	0.09113686	1.66E-22
Gzmk	0.02655267	2.55E-34
Gzmm	0	1.53E-08
Icos	0.05887565	1.37E-47
Ifng	0.07432152	5.90E-35
Il17a	0	0.00214506
Il18rap	0.26007444	9.19E-27
Il2	0	0.01214877
Il23r	0	4.82E-06
Il7r	0.21730239	2.63E-18
Klra1	0.01735017	9.42E-72
Klra21	0	4.81E-39

Klra3	0	4.98E-28
Klra5	0	8.56E-06
Klra6	0	3.68E-38
Klra7	0.08832814	2.13E-24
Klrb1	0	1.95E-07
Klrc1	0.06459856	9.04E-31
Klrc3	0	2.23E-07
Klrg1	0	5.22E-42
Klrk1	0.28073293	3.49E-16
Lag3	0	1.60E-38
Prf1	0.09668416	9.86E-11
Stat4	0.14781749	1.41E-56
Tbx21	0.11333893	4.52E-34
Tcf7	0.12341657	1.91E-42
Tigit	0.03129246	1.35E-75
Tnfrsf13c	0	3.45E-13
Tnfrsf18	0.10089767	8.24E-45
Tnfrsf25	0.09742788	3.99E-11
Tnfrsf4	0.03782666	3.33E-28
Tnfrsf9	0.04004308	3.11E-60
Tnfsf10	0.303975	0.00656804
Tnfsf8	0.16186102	3.01E-07
Xcl1	0.11245181	3.95E-14

Cluster-10

[GeneSet]		
$SchemaVersion	1	
$GeneSetName	Down_in_Phenograph_7FX1_10_RunID:_SAAT	
$GeneSetDescription	Generated in the iCellR Pipeline. Fold-Change < 0.5; q-Val (FDR) < 0.05	
[Genes]	Fold-Change	q-Value
CD103:M290 (Ab)	0.22339902	1.58E-08
CD272 (Ab)	0.44196436	3.14E-17
CD335 (Ab)	0.36337259	2.28E-22
NK-1.1 (Ab)	0.21591154	1.06E-67
Bach2	0.12063077	8.66E-15
Bcl11a	0	4.44E-17
Bcl2	0.46530875	0.00097614
Bcl6	0.18054136	1.67E-06

Cd274	0.4885804	0.00729198
Cx3cr1	0	0.04051013
Foxp3	0.12172245	7.78E-10
Gzma	0.15363446	1.14E-21
Il17a	0.06113787	0.01040734
Il2	0	0.01531826
Il23r	0	6.41E-06
Il7r	0.1423895	4.04E-35
Irf7	0.46792418	6.41E-06
Klra1	0	4.07E-83
Klra17	0	1.75E-12
Klra21	0.19540433	3.07E-07
Klra5	0	1.10E-05
Klra7	0.00667842	2.82E-108
Klrc3	0	2.97E-07
Tcf7	0.18467274	4.45E-14
Tnfrsf13b	0.10399741	5.75E-24
Tnfrsf13c	0	4.73E-13
Tnfrsf25	0.10346409	5.63E-10
Tnfsf13	0.20226062	7.71E-07
Tnfsf8	0.34598923	0.02060442

Cluster-11

[GeneSet]		
$SchemaVersion	1	
$GeneSetName	Down_in_Phenograph_7FX1_11_RunID:_SAAT	
$GeneSetDescription	Generated in the iCellR Pipeline. Fold-Change < 0.5; q-Val (FDR) < 0.05	
[Genes]	Fold-Change	q-Value
CD103:M290 (Ab)	0.16228877	8.60E-11
CD119 (Ab)	0.18512812	4.38E-38
CD25:PC61 (Ab)	0.15456637	1.89E-29
CD272 (Ab)	0.22113267	3.20E-28
CD279:J43 (Ab)	0.47785407	6.91E-06
CD335 (Ab)	0.249641	7.11E-40
CD3:145-2C11 (Ab)	0.3693929	2.87E-09
NK-1.1 (Ab)	0.18353251	9.38E-68

Bach2	0.08191515	2.04E-16
Bcl11a	0	2.42E-17
Bcl2a1a	0.0240526	5.66E-91
Btla	0	2.83E-72
Cd244	0.10613354	2.58E-10
Cd247	0	1.49E-255
Cd274	0.231992	3.43E-08
Cd28	0	4.80E-139
Ctla4	0.01014015	1.44E-41
Cx3cr1	0	0.0249304 2
Eomes	0	6.58E-121
Foxp3	0	2.70E-27
Gata3	0	3.68E-26
Gzma	0.00660225	8.07E-112
Gzmb	0.0138226	4.53E-63
Gzmk	0.0768438	1.24E-21
Icos	0.04250269	2.16E-40
Ifng	0.00892857	7.82E-65
Il17a	0	0.0015097 1
Il18rap	0.04257683	3.26E-46
Il2	0	0.0089802 8
Il23r	0	3.94E-06
Il4ra	0.05706401	2.53E-45
Il7r	0.01483633	1.33E-143
Irf7	0.30893971	0.0025184 9
Klra1	0.0250795	2.01E-61
Klra17	0	9.87E-13
Klra21	0	3.56E-39
Klra3	0	3.52E-28
Klra5	0	6.91E-06
Klra6	0	2.69E-38
Klra7	0.00909039	1.20E-105
Klrb1	0	1.42E-07
Klrc1	0.01242276	1.85E-165
Klrc3	0	1.60E-07
Klrg1	0	4.13E-42
Klrk1	0.00933607	1.43E-220
Lag3	0.03790484	4.59E-28
Prf1	0	2.50E-34

Stat4	0	1.54E-304
Tbx21	0	9.24E-177
Tcf7	0.14481562	1.95E-46
Tigit	0	3.26E-101
Tnf	0	1.93E-57
Tnfrsf13b	0	1.05E-72
Tnfrsf13c	0	2.71E-13
Tnfrsf18	0.02565348	3.77E-138
Tnfrsf1b	0	1.97E-193
Tnfrsf25	0	1.06E-39
Tnfrsf4	0.0364339	1.06E-27
Tnfrsf8	0	2.34E-05
Tnfrsf9	0.06948021	8.55E-39
Tnfsf10	0.14530187	8.37E-06
Tnfsf13b	0	2.70E-10
Tnfsf14	0	6.01E-51
Tnfsf8	0	1.92E-30
Xcl1	0.00567911	1.03E-48

Cluster 12

[GeneSet]		
$SchemaVersion	1	
$GeneSetName	Down_in_Phenograph_7FX1_12_RunID:_SAAT	
$GeneSetDescription	Generated in the iCellR Pipeline. Fold-Change < 0.5; q-Val (FDR) < 0.05	
[Genes]	Fold-Change	q-Value
CD103:M290 (Ab)	0.07835584	1.20E-26
CD119 (Ab)	0.09775582	9.01E-112
CD223 (Ab)	0.37237117	2.24E-08
CD25:PC61 (Ab)	0.13376934	1.20E-32
CD272 (Ab)	0.15148707	2.12E-84
CD274 (Ab)	0.10370085	4.39E-88
CD279:J43 (Ab)	0.4411461	9.90E-14
CD335 (Ab)	0.25349769	1.20E-49
CD3:145-2C11 (Ab)	0.26287611	4.13E-41
NK-1.1 (Ab)	0.21439896	7.06E-83
Bach2	0.09201342	2.02E-13
Bcl2	0.30825482	1.64E-06
Bcl2a1a	0.04053992	1.99E-77
Btla	0	2.67E-72
Cd244	0	2.14E-55

Cd247	0	1.95E-255
Cd274	0.43616119	0.0299848<br>6
Cd28	0.06487382	9.34E-39
Ctla4	0.0113902	2.86E-41
Cx3cr1	0	0.0264889<br>3
Eomes	0	7.35E-121
Foxp3	0	2.84E-27
Gzma	0.00185379	3.45E-113
Gzmb	0	8.71E-69
Gzmk	0.04311321	4.61E-27
Icos	0.04774231	2.83E-49
Ifng	0.02006341	4.23E-61
Il17a	0	0.0016686<br>9
Il18rap	0.05979936	2.71E-65
Il2	0	0.0105652<br>6
Il23r	0	4.24E-06
Il7r	0	1.19E-186
Klra1	0	3.78E-83
Klra17	0	1.04E-12
Klra21	0	3.44E-39
Klra3	0	3.53E-28
Klra5	0	7.74E-06
Klra6	0	2.60E-38
Klra7	0	5.82E-112
Klrb1	0	1.61E-07
Klrc1	0.0139542	1.17E-154
Klrc3	0	1.83E-07
Klrg1	0	3.94E-42
Klrk1	0	4.52E-253
Lag3	0.29990625	8.74E-05
Prf1	0	2.46E-34
Stat4	0.02513291	1.52E-90
Tbx21	0.02291365	4.78E-97
Tcf7	0.00623276	9.43E-291
Tigit	0	3.53E-101
Tnf	0.24987849	1.71E-05
Tnfrsf13b	0	9.95E-73
Tnfrsf18	0.03843027	2.06E-93
Tnfrsf1b	0.31103648	6.44E-08

Tnfrsf25	0	1.02E-39
Tnfrsf4	0.04092537	9.24E-27
Tnfrsf8	0	2.76E-05
Tnfrsf9	0	4.55E-76
Tnfsf10	0.16321429	0.00014247
Tnfsf14	0	5.68E-51
Tnfsf8	0	1.85E-30
Xcl1	0.00318936	4.65E-49

Cluster-13

[GeneSet]		
$SchemaVersion	1	
$GeneSetName	Down_in_Phenograph_7FX1_13_RunID:_SAAT	
$GeneSetDescription	Generated in the iCellR Pipeline. Fold-Change < 0.5; q-Val (FDR) < 0.05	
[Genes]	Fold-Change	q-Value
CD103:M290 (Ab)	0.05830517	6.00E-28
CD119 (Ab)	0.20002624	5.13E-68
CD25:PC61 (Ab)	0.13585293	2.05E-29
CD274 (Ab)	0.48992229	7.18E-09
CD278 (Ab)	0.44763696	1.37E-05
CD335 (Ab)	0.18500315	1.60E-63
CD3:145-2C11 (Ab)	0.23352234	1.69E-24
IL-33R (Ab)	0.33895978	4.38E-06
NK-1.1 (Ab)	0.29137097	1.16E-22
Bcl2	0.06118499	5.31E-24
Bcl2a1a	0.03147996	1.09E-84
Cd247	0.05291066	1.33E-42
Cd274	0.24241892	1.82E-06
Cd28	0.22732762	5.49E-07
Ctla4	0	3.47E-43
Cx3cr1	0	0.02804798
Eomes	0	8.82E-121
Foxp3	0	3.52E-27
Gata3	0	4.73E-26
Gzma	0.0051847	7.09E-112
Gzmb	0	1.10E-68
Gzmk	0	3.20E-41
Gzmm	0	1.39E-08
Icos	0.03336072	1.01E-48

Ifng	0.01402436	1.09E-61
Il17a	0	0.0017483
Il18rap	0.10037328	1.24E-24
Il2	0	0.0110940 5
Il23r	0	4.38E-06
Klra1	0	4.62E-83
Klra21	0	4.25E-39
Klra3	0	4.44E-28
Klra5	0	7.74E-06
Klra6	0	3.29E-38
Klra7	0.01427852	1.69E-96
Klrb1	0	1.71E-07
Klrc1	0	4.52E-201
Klrc3	0	1.95E-07
Klrg1	0	4.74E-42
Klrk1	0.20598476	1.48E-15
Prf1	0	3.12E-34
Stat4	0.15848898	5.71E-27
Tbx21	0.09623071	3.08E-12
Tcf7	0.02615457	6.01E-74
Tigit	0.17795139	5.35E-09
Tnfrsf18	0.09410875	8.26E-50
Tnfrsf1b	0.33200735	2.68E-08
Tnfrsf25	0	1.26E-39
Tnfrsf4	0	1.05E-32
Tnfrsf8	0	2.55E-05
Tnfrsf9	0	5.67E-76
Tnfsf10	0	2.09E-43
Tnfsf13	0.14342616	1.37E-05
Tnfsf13b	0	3.50E-10
Tnfsf14	0	7.11E-51
Tnfsf8	0	2.36E-30
Xcl1	0.00445982	9.99E-49

Dataset S2: Upregulated Genes in Clusters 1-13

Cluster-1

[GeneSet]		
$SchemaVersion	1	
$GeneSetName	Up_in_Phenograph_7FX1_1_RunID:_SAAT	
$GeneSetDescription	Generated in the iCellR Pipeline. Fold-Change > 2; q-Val (FDR) < 0.05	
[Genes]	Fold-Change	q-Value
CD335 (Ab)	8.53919245	2.34E-102
NK-1.1 (Ab)	6.11663685	1.02E-119
Bcl2	2.52470855	1.41E-21
Cd244	2.10054648	1.30E-06
Eomes	9.7647038	2.05E-81
Gzma	40.5359829	4.32E-121
Gzmb	3.72726915	3.57E-20
Ifng	2.27681391	7.86E-12
Il18rap	3.35976002	8.83E-87
Klra1	7.33911915	7.84E-46
Klra21	21.3143175	9.61E-32
Klra3	9.60104394	1.38E-16
Klra6	2.92672428	5.38E-08
Klra7	14.065936	3.01E-87
Klrb1	12.0631383	5.38E-05
Klrc1	6.20224063	2.83E-121
Klrc3	19.8001857	1.04E-05
Klrg1	2.48630128	5.41E-06
Klrk1	4.7035147	1.18E-119
Prf1	4.45168202	1.23E-13
Stat4	2.20964668	2.85E-47
Tbx21	3.61702004	2.46E-52
Tnfsf14	2.15392834	5.57E-05
Xcl1	11.9962594	3.91E-33

Cluster-2

[GeneSet]		
$SchemaVersion	1	
$GeneSetName	Up_in_Phenograph_7FX1_2_RunID:_SAAT	
$GeneSetDescription	Generated in the iCellR Pipeline. Fold-Change > 2; q-Val (FDR) < 0.05	
[Genes]	Fold-Change	q-Value
Cd28	2.1600854	4.63E-12
Gzmk	4.20042435	7.14E-13
Icos	2.34105094	1.86E-11
Tnfsf8	2.05658481	0.03776015

Cluster-3

[GeneSet]		
$SchemaVersion	1	
$GeneSetName	Up_in_Phenograph_7FX1_3_RunID:_SAAT	
$GeneSetDescription	Generated in the iCellR Pipeline. Fold-Change > 2; q-Val (FDR) < 0.05	
[Genes]	Fold-Change	q-Value
Bcl2	2.26429421	3.49E-10
Klra1	2.45720116	4.29E-09
Klra5	11.4987913	0.02573689
Klra6	5.13987874	4.90E-10
Tcf7	2.22872554	6.55E-34
Tnfsf10	2.00123997	0.01789803
Tnfsf8	2.64387146	7.75E-06

Cluster-4

[GeneSet]		
$SchemaVersion	1	
$GeneSetName	Up_in_Phenograph_7FX1_4_RunID:_SAAT	
$GeneSetDescription	Generated in the iCellR Pipeline. Fold-Change > 2; q-Val (FDR) < 0.05	
[Genes]	Fold-Change	q-Value
CD103:M290 (Ab)	2.87714435	0.02880545

CD25:PC61 (Ab)	2.63984709	0.0183926
CD272 (Ab)	4.15923246	8.61E-31
CD274 (Ab)	2.24224221	4.82E-05
Bax	2.16098776	8.26E-15
Bcl11a	8.43294988	3.65E-07
Bcl2a1a	3.85893749	4.38E-10
Bcl6	7.5708334	3.71E-15
Btla	7.8018338	1.87E-20
Cd244	4.02939154	1.71E-08
Cd274	3.30768552	6.34E-06
Il4ra	4.37862918	1.04E-27
Irf7	4.65012442	6.68E-19
Klra17	4.20990381	0.01085304
Tnf	2.64062067	0.00312671
Tnfrsf13b	5.60673826	8.95E-20
Xbp1	3.55399054	2.40E-36

Cluster-5

[GeneSet]		
$SchemaVersion	1	
$GeneSetName	Up_in_Phenograph_7FX1_5_RunID:_SAAT	
$GeneSetDescription	Generated in the iCellR Pipeline. Fold-Change > 2; q-Val (FDR) < 0.05	
[Genes]	Fold-Change	q-Value
CD3:145-2C11 (Ab)	4.96555237	1.17E-65
Bach2	2.15638876	0.00139794
Il7r	2.78485279	2.36E-19
Tcf7	4.05092914	8.96E-64
Tnfsf8	2.12935051	0.01620748

Cluster-6

[GeneSet]		
$SchemaVersion	1	
$GeneSetName	Up_in_Phenograph_7FX1_6_RunID:_SAAT	
$GeneSetDescription	Generated in the iCellR Pipeline. Fold-Change > 2; q-Val (FDR) < 0.05	

[Genes]	Fold-Change	q-Value
CD103:M290 (Ab)	6.081260739	5.86E-06
CD25:PC61 (Ab)	4.618370346	7.80E-11
CD274 (Ab)	2.124195715	9.37E-13
CD278 (Ab)	5.250804262	1.31E-09
CD279:J43 (Ab)	3.782755031	6.11E-06
CD3:145-2C11 (Ab)	2.219223761	5.71E-10
IL-33R (Ab)	16.72080406	5.09E-14
Gata3	3.38593482	0.047278296
Icos	2.584366577	1.15E-05
Il17a	297.9622642	0.003325309
Il23r	72.36226415	6.64E-05
Il7r	3.447742907	7.14E-20
Klrg1	2.689009222	0.013461375
Tnfrsf25	6.824833983	1.03E-09
Tnfsf14	2.784694057	0.000573781

Cluster-7

[GeneSet]		
$SchemaVersion	1	
$GeneSetName	Up_in_Phenograph_7FX1_7_RunID:_SAAT	
$GeneSetDescription	Generated in the iCellR Pipeline. Fold-Change > 2; q-Val (FDR) < 0.05	
[Genes]	Fold-Change	q-Value
CD272 (Ab)	11.8873569	4.74E-40
Bach2	3.19623887	8.67E-05
Btla	4.84297442	5.66E-14
Tnfrsf13b	2.38562092	0.00025818
Tnfrsf13c	28.627451	5.11E-07

Cluster-8

[GeneSet]		
$SchemaVersion	1	
$GeneSetName	Up_in_Phenograph_7FX1_8_RunID:_SAAT	
$GeneSetDescription	Generated in the iCellR Pipeline. Fold-Change > 2; q-Val (FDR) < 0.05	
[Genes]	Fold-Change	q-Value
CD103:M290 (Ab)	4.882860022	3.30E-06

CD137 (Ab)	2.276633851	4.43E-09
CD25:PC61 (Ab)	8.920750404	5.77E-14
CD278 (Ab)	4.631623801	5.70E-10
IL-33R (Ab)	2.368694887	0.00063472
Cd28	3.170836167	1.39E-07
Ctla4	23.81768368	1.21E-16
Foxp3	101.4067982	9.18E-30
Icos	8.238367495	1.26E-12
Klrg1	4.434359417	0.000722255
Tigit	5.978885805	1.55E-13
Tnfrsf18	7.348434544	1.41E-24
Tnfrsf1b	3.546313027	3.39E-11
Tnfrsf4	30.07028097	7.82E-18
Tnfrsf8	28.27265625	0.026883718
Tnfrsf9	9.46357031	1.91E-15

Cluster-9

[GeneSet]		
$SchemaVersion	1	
$GeneSetName	Up_in_Phenograph_7FX1_9_RunID:_SAAT	
$GeneSetDescription	Generated in the iCellR Pipeline. Fold-Change > 2; q-Val (FDR) < 0.05	
[Genes]	Fold-Change	q-Value
IL-17RB (Ab)	2.06558097	6.49E-06
Bcl6	3.56371198	0.00214506
Cd244	3.31127451	0.0150442
Il4ra	3.11292589	3.72E-07
Irf7	4.27572545	4.40E-09
Tnfrsf13b	2.8588125	0.00033216
Tnfrsf1b	4.53615305	2.96E-14
Tnfsf13	22.0322965	3.97E-14
Xbp1	4.88192318	8.80E-25

Cluster-10

[GeneSet]		
$SchemaVersion	1	
$GeneSetName	Up_in_Phenograph_7FX1_10_RunID:_SAAT	
$GeneSetDescription	Generated in the iCellR Pipeline. Fold-Change > 2; q-Val (FDR) < 0.05	

[Genes]	Fold-Change	q-Value
CD223 (Ab)	11.6215481	7.09E-06
CD279:J43 (Ab)	4.11880124	0.00153621
Bax	2.6277137	1.19E-11
Bcl2a1a	6.4650662	3.46E-23
Cd247	4.72893486	5.33E-21
Cd28	3.44022679	5.25E-08
Ctla4	4.18225267	2.25E-06
Eomes	2.68357371	7.01E-06
Gzmb	8.49934021	2.28E-13
Gzmk	21.9398937	3.19E-16
Ifng	9.48130826	4.08E-07
Klrc1	2.75040416	1.33E-06
Lag3	24.1051032	2.30E-17
Prf1	12.8458219	5.45E-08
Stat4	2.23282613	7.61E-08
Tbx21	2.84908492	1.18E-07
Tigit	10.2108046	4.19E-22
Tnfrsf18	2.38524673	2.23E-06
Tnfrsf1b	3.53390318	4.30E-14
Tnfrsf4	3.81677955	0.00080416
Tnfrsf9	6.90389864	4.88E-16
Tnfsf10	4.46278268	0.00153621
Tnfsf13b	11.8100468	0.01040734

Cluster-11

[GeneSet]		
$SchemaVersion	1	
$GeneSetName	Up_in_Phenograph_7FX1_11_RunID:_SAAT	
$GeneSetDescription	Generated in the iCellR Pipeline. Fold-Change > 2; q-Val (FDR) < 0.05	
[Genes]	Fold-Change	q-Value
Bax	5.98915033	5.32E-06

Cluster-12

[GeneSet]		
$SchemaVersion	1	
$GeneSetName	Up_in_Phenograph_7FX1_12_RunID:_SAAT	
$GeneSetDescription	Generated in the iCellR Pipeline. Fold-Change > 2; q-Val (FDR) < 0.05	
[Genes]	Fold-Change	q-Value
CD137 (Ab)	2.03857608	0.00023802
Tnfsf13	2.96136	0.02030858
Tnfsf13b	21.0923077	0.01056526
Xbp1	2.74288338	9.78E-08

Cluster-13

[GeneSet]		
$SchemaVersion	1	
$GeneSetName	Up_in_Phenograph_7FX1_13_RunID:_SAAT	
$GeneSetDescription	Generated in the iCellR Pipeline. Fold-Change > 2; q-Val (FDR) < 0.05	
[Genes]	Fold-Change	q-Value
CD223 (Ab)	9.70663996	2.51E-06
Bcl11a	33.4202327	7.74E-06
Btla	3.37820199	0.01964221
Il4ra	2.00665742	0.02189119
Irf7	3.71991458	0.00153129
Klra17	118.513468	3.50E-10
Lag3	10.145394	1.82E-09
Tnfrsf13b	14.1714222	1.33E-10
Xbp1	5.41687321	5.58E-11

Dataset S3

One-Way Anova Analysis with Turkeys Multiple Comparison Test for tumor volume.		
**Day 9**		
**Cohorts**	P value	Summary
Isotype Control vs anti-NK1.1	0.2765	ns
Isotype Control vs anti-PD-1	0.0062	**
Isotype Control vs anti-NK1.1+anti-PD-1	0.0953	ns
anti-NK1.1 vs anti-PD1	0.2826	ns
anti-NK1.1 vs anti-NK1.1+ anti-PD-1	0.9723	ns
anti-PD-1 vs anti-NK1.1+anti PD1	0.4211	ns
**Day 10**		
**Cohorts**	P value	
Isotype Control vs anti-NK1.1	0.0217	*
Isotype Control vs anti-PD-1	0.0001	***
Isotype Control vs anti-NK1.1+anti-PD-1	0.0024	**
anti-NK1.1 vs anti-PD1	0.1253	ns
anti-NK1.1 vs anti-NK1.1+ anti-PD-1	0.8993	ns
anti-PD-1 vs anti-NK1.1+anti PD1	0.2905	ns
**Day 11**		
**Cohorts**	P value	
Isotype Control vs anti-NK1.1	0.029	*
Isotype Control vs anti-PD-1	<0.0001	****
Isotype Control vs anti-NK1.1+anti-PD-1	0.0007	***
anti-NK1.1 vs anti-PD1	0.0688	ns
anti-NK1.1 vs anti-NK1.1+ anti-PD-1	0.5274	ns
anti-PD-1 vs anti-NK1.1+anti PD1	0.4652	ns
**Day 12**		
**Cohorts**	P value	
Isotype Control vs anti-NK1.1	0.2936	ns
Isotype Control vs anti-PD-1	<0.0001	****
Isotype Control vs anti-NK1.1+anti-PD-1	0.0005	***
anti-NK1.1 vs anti-PD1	0.0015	**
anti-NK1.1 vs anti-NK1.1+ anti-PD-1	0.0556	ns
anti-PD-1 vs anti-NK1.1+anti PD1	0.2145	ns
**Day 13**		
**Cohorts**	P value	

Isotype Control vs anti-NK1.1	0.2592	ns
Isotype Control vs anti-PD-1	<0.0001	****
Isotype Control vs anti-NK1.1+anti-PD-1	0.0012	**
anti-NK1.1 vs anti-PD1	0.0035	**
anti-NK1.1 vs anti-NK1.1+ anti-PD-1	0.1219	ns
anti-PD-1 vs anti-NK1.1+anti PD1	0.2141	ns
**Day 14**		
**Cohorts**	P value	
Isotype Control vs anti-NK1.1	0.8068	ns
Isotype Control vs anti-PD-1	<0.0001	****
Isotype Control vs anti-NK1.1+anti-PD-1	<0.0001	****
anti-NK1.1 vs anti-PD1	<0.0001	****
anti-NK1.1 vs anti-NK1.1+ anti-PD-1	0.0002	***
anti-PD-1 vs anti-NK1.1+anti PD1	0.3304	ns
Day 15		
**Cohorts**	P value	
Isotype Control vs anti-NK1.1	0.4476	ns
Isotype Control vs anti-PD-1	<0.0001	****
Isotype Control vs anti-NK1.1+anti-PD-1	<0.0001	****
anti-NK1.1 vs anti-PD1	<0.0001	****
anti-NK1.1 vs anti-NK1.1+ anti-PD-1	<0.0001	****
anti-PD-1 vs anti-NK1.1+anti PD1	0.075	ns

SI References

1. Stathopoulou, C. *et al.* PD-1 Inhibitory Receptor Downregulates Asparaginyl Endopeptidase and Maintains Foxp3 Transcription Factor Stability in Induced Regulatory T Cells. *Immunity* **49**, 247-263 e247 (2018).

2. Schatton, T. *et al.* Identification of cells initiating human melanomas. *Nature* **451**, 345-349 (2008).

3. Parang, B., Barrett, C.W. & Williams, C.S. AOM/DSS Model of Colitis-Associated Cancer. *Methods Mol Biol* **1422**, 297-307 (2016).

4. Mallett, G., Patterson, W., Payne, M. & Amarnath, S. Isolation and Characterization of Innate Lymphoid Cells within the Murine Tumor Microenvironment. *Methods Mol Biol* **2121**, 153-164 (2020).
